# A Quantitative Tandem Mass Spectrometry and Scaled-Down QuEChERS Approach for Simultaneous Analysis of Pesticide Multiresidues in Human Urine

**DOI:** 10.3390/molecules24071330

**Published:** 2019-04-04

**Authors:** Yongho Shin, Jiho Lee, Eunyoung Park, Junghak Lee, Hye Suk Lee, Jeong-Han Kim

**Affiliations:** 1 Pesticide Chemistry and Toxicology Laboratory, Department of Agricultural Biotechnology and Research Institute of Agriculture and Life Sciences, Seoul National University, Seoul 08826, Korea; driger6103@catholic.ac.kr (Y.S.); micai1@snu.ac.kr (J.L.); pey4321@snu.ac.kr (E.P.); crane245@snu.ac.kr (J.L.); 2 Drug Metabolism and Bioanalysis Laboratory, College of Pharmacy, The Catholic University of Korea, Bucheon-si, Gyeonggi-do 14662, Korea

**Keywords:** pesticide, multiresidue, forensic, urine, QuEChERS, LC-MS/MS

## Abstract

Multiresidual pesticide determination in a biological sample is essential for an immediate decision and response related to various pesticide intoxications. A rapid and simultaneous analytical method for 260 pesticides in human urine was developed and validated using liquid chromatography-tandem mass spectrometry (LC-MS/MS). High speed positive/negative switching electrospray ionization (ESI) mode was used, and scheduled multiple reaction monitoring (MRM) was optimized. Three versions of scaled-down QuEChERS procedures were evaluated, and the procedure using non-buffer reagents (magnesium sulfate and sodium chloride) and excluding cleanup steps was selected for optimum pesticide extraction. The limit of quantitation (LOQ) in this methodology was 10 ng/mL for each target pesticide, and correlation coefficient (r^2^) values of calibration curves were ≥0.988 (linearity range; 10–250 ng/mL). In accuracy and precision tests, the relative error ranges were −18.4% to 19.5%, with relative standard deviation (RSD) 2.1%–19.9% at an LOQ level (10 ng/mL), and −14.7% to 14.9% (RSD; 0.6%–14.9%) at higher concentrations (50, 150, and 250 ng/mL). Recovery range was 54.2%–113.9% (RSD; 0.3%–20.0%), and the soft matrix effect (range; −20% to 20%) was observed in 75.4% of target pesticides. The established bioanalytical methods are sufficient for application to biomonitoring in agricultural exposures and applicable in the forensic and clinic.

## 1. Introduction

Pesticides have been widely applied on farms for control of problematic weeds or harmful pests such as certain insects and fungi. The use of pesticides has contributed to the improvement of crop/livestock yields and quality, increased shelf life of produce, and prevention of harmful organisms from interfering in human activities and structures, from which secondary benefits such as national agricultural economic development, reduced maintenance costs, or quality of life improvement have followed [1]. Despite the considerable advantages of pesticides, unwanted side effects have also followed. Pesticide intoxication resulting from intentional intake or misuse during cultivation is a major social problem. Gunnell and coworkers investigated the global distribution of suicide by pesticide and estimated that there are 258234 (plausible range from 233997 to 325907) suicides from pesticide poisoning each year, representing 30% (27% to 37%) of all suicides worldwide [2]. In the United States, 234 deaths by pesticide poisoning were identified over a 10-year span (1999 to 2008) according to the Centers for Disease Control and Prevention’s Wide-ranging Online Data for Epidemiologic Research (CDC WONDER) report, and an average of 20116 people were exposed to pesticides annually, accounting for 17.8% of treatment in health care facilities from 2006 to 2010 [3]. In the Republic of Korea, 16161 reports of mortality and 45291 reports of inpatient and outpatient treatment related to pesticide intoxication were reported during 5 years (2006 to 2010) [4].

Biological monitoring of acute or chronic pesticide poisoning is useful for identifying evidence of health problems in the environment/ecotoxicology, agricultural and forensic fields, or for detoxification in a medical institution. Human biological samples such as urine [5,6,7,8,9], blood [10,11,12], hair [13,14], and saliva [15] have been the primary sources for determination of pesticides. Among them, urine has several advantages over other samples. Urine is easier to obtain than invasive samples such as blood, and larger amounts of urine are available compared with blood, hair, and saliva. Because urine is a homogeneous biological fluid composed of 95% water [6], complex preparation steps for purification of target pesticides are not needed.

Although some pesticides are metabolized rapidly in the body and excreted in urine within 48 h [16], various chemical groups of pesticides still remain intact and present in urine [17,18,19,20]. It is easier and less costly to obtain analytical standards of pesticides rather than those of metabolites. Detecting as many pesticides as possible is also needed because there have been deaths resulting from various chemical groups of pesticides [21], some of them (e.g., benzoximate and etofenprox) showing very low acute toxicity (LD_50_ > 10,000 mg/kg; oral acute for rat) [22].

For multiresidual pesticide analysis, mass spectrometry coupled with chromatography is widely utilized. A single quadrupole (SQ) mass filter has a high scan speed technique using selected ion monitoring (SIM), therefore it can determine more than one hundred of target compounds simultaneously. However, the SQ is not able to distinguish an analyte from other analytes or interferences with overlapped retention time (t_R_) and the same mass to charge ratio (*m/z*). A high-resolution mass spectrometry has an excellent selectivity useful for screening target and non-target compounds, whereas it has limitations on targeted multiresidue determination due to its low scan speed. With the introduction of triple quadrupole (TQ) mass filters, analysis of more than 200 target compounds at once became feasible [23]. The multiple reaction monitoring (MRM) mode of the TQ can select and detect target pesticides with high-throughput and selectivity.

The aqueous characteristic of a urine and advanced separation techniques such as LC-MS/MS make urine preparation relatively convenient and easy. Direct injection [6] or dilute-and-shoot [24,25] procedures are the simplest ways to identify pesticides in urine. Nevertheless, these processes have major problems in that urinary salts or macromolecules may decrease the sensitivity of an instrument or cause severe clogging on the injection syringe or ESI probe. Solid phase extraction (SPE) [5,26,27] and liquid-liquid extraction (LLE) [28] have been popular preparation methods for pesticide extraction. However, these procedures also have disadvantages. SPE is not useful for multiresidue analysis due to the difficulty of finding optimum washing/elution conditions covering various chemical properties. LLE is simpler and easier than SPE, but interferences from urine may remain in the extract and cause a serious matrix effect or lead to low extraction efficiency. The Quick, Easy, Cheap, Effective, Rugged, and Safe (QuEChERS) method introduced by Anastassiades and coworkers is one of the most effective methodologies overcoming the limits or drawbacks of other preparations [29]. The original QuEChERS optimized for crop samples has been modified according to analytical situations [30,31,32,33] and extended for matrix materials such as biological samples [34,35,36].

The aim of this study is to develop and validate a reliable quantitative method for 260 pesticides in human urine utilizing liquid chromatography-tandem mass spectrometry (LC-MS/MS). The high speed switchable positive/negative ESI mode of LC-MS/MS is used, and scheduled MRM has been established for each pesticide to analyze within a short time of 15 min. For effective and rugged multiresidue extraction, three different versions of QuEChERS were tried and the optimum procedure was established. This analytical method would be applicable for the monitoring of pesticide multiresidues in urinary samples in environmental, agricultural and forensic science facilities or in hospitals for clinical purposes.

## 2. Results and Discussion

### 2.1. Recovery of Three Versions of Urine Preparation Methods

Urine is a liquid sample, so it is appropriate to prepare the sample using simple QuEChERS methods for high extraction efficiency (recovery) of pesticide multiresidues. Since the first QuEChERS method for crops was developed using GC-MS in 2003 [29], there have been preparation procedure improvements for LC-amenable pesticides or lower recovery rate compounds such as pH-dependent pesticides [37,38]. In this study, optimization of the final preparation step was established by comparing the scaled-down methods from three representative QuEChERS procedures [29,30,31]. During the extraction procedure, the sample was cooled in an ice bath to prevent pesticide degradation by the heat generated from the reaction between MgSO_4_ and water. Because the volume of urine in the preparation was very small (100 μL), the extraction solvent volume and the quantity of QuEChERS partitioning reagents were also reduced (Table 1).

To verify the recoveries for methods A, B, and C, each peak area of a pre- and a post-spiked (matrix-matched) samples at 250 ng/mL was compared. In accordance with SANTE/11813/2017 guidelines [39], the number of pesticides satisfying the recovery range of 70%–120% and relative standard deviation (RSD) ≤ 20% was verified for the 260 compounds. The results showed that 99.2% (A), 98.5% (B), and 99.2% (C) of total pesticides satisfied the criteria, respectively (Table 1 and Table S1). Method A and C showed slightly more compounds satisfying the criteria than method B. Method A showed consistent repeatability (RSD ≤ 20%) in all compounds. In method C, however, cartap was not recovered at all (Table S1). Average recovery of method A was also superior (92.3%) to those of methods B (87.3%) and C (84.9%).

### 2.2. Relative Peak Intensity of Three Versions of Urine Preparation Methods

The relative peak intensity of each method was verified to compare a post-spiked sample and a solvent (non-matrix contained) standard at 10 and 250 ng/mL (Table S2). When the area value of each pesticide peak in the solvent standard is converted to 100, a relative peak intensity under/above 100 means that the signal is suppressed/enhanced by urine matrices. Relative area range distribution showed that method A had the largest numbers of compounds (72.7% and 74.6% of 260 compounds) within 80–120 than method B (57.7% and 60.4%) and C (47.3% and 48.5%) at both concentrations (Table 2). The results indicate that matrix effects (especially signal suppression) were much reduced in method A than B and C. The first reason is that method A used only two types of reagents (MgSO_4_ and NaCl) and no acids or buffers (Table 1), so matrix effect from the reagents themselves could be minimized. The other reason is that acidic or buffer reagents in method B and C extracted different kinds of matrices or excessive unnecessary matrices than those in method A, thus these matrices may increase matrix effects.

From the recovery and the intensity results, method A was selected as the final procedure for extraction and partitioning. Cleanup procedures such as dispersive SPE (dSPE) were excluded to prevent the loss of labile target compounds and to minimize the preparation time.

**Figure 1:**
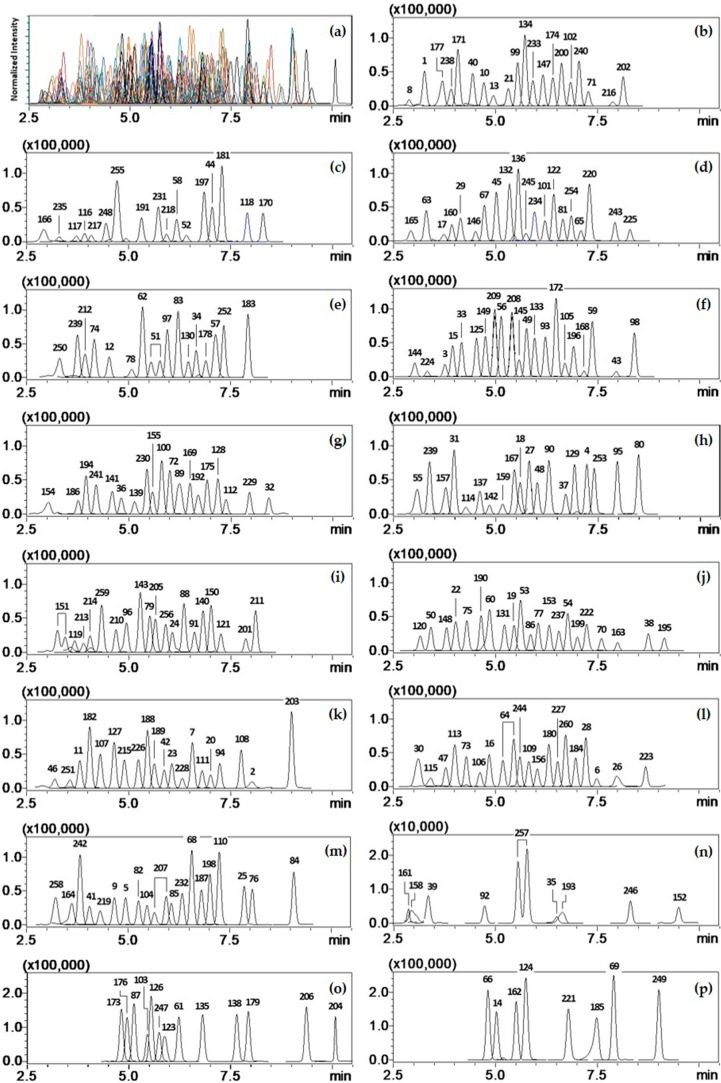
TIC obtained by MRM mode of LC-MS/MS at LOQ (10 ng/mL). (**a**) Chromatograms for 260 pesticides in urine sample (4 µL injection); (**b**) to (**p**) Individual chromatograms separated from (**a**). Pesticide numbers assigned on the peaks and their MRM transitions (quantifier) are designated in Table 3.

Analytical conditions (retention times, MRM transitions, and ratio) were shown in Table 3, and total ion chromatograms (TICs) of 260 pesticides in urine are given in Figure 1.

### 2.3. Method Validation

Analytical method validation was conducted for the 260 multiresidual pesticides in urine. The validation parameters to be determined were limit of quantitation (LOQ), linearity of calibration, accuracy/precision, recovery, and matrix effect (Table 4).

#### 2.3.1. Limit of Quantitation (LOQ) and Linearity of Calibration

One of the methods for determining LOQ is to find the minimum concentration with a peak S/N greater than 10 on the chromatogram [41]. It is easy but does not guarantee reproducibility in an analytical method. Therefore, relative error (RE, %) and RSD in accuracy and precision study (Table 4) should be supplemented for the rugged LOQ validation. At 10 ng/mL, target analytes were found to have an S/N > 10 as well as reasonable RE (−18.4% to 19.5%) and RSD (2.1% to 19.7%) ranges. Those compounds with this concentration have sufficiently low detectability for various applications because urinary concentrations of parent compounds have been reported to range from sub to hundreds of ng/mL in cases of acute pesticide intoxication or in some biomonitoring investigations [17,20,26,42]. Therefore, with the established analytical method, 260 pesticides can be determined in a urinary sample without further concentration of the sample extract.

Except for the target compounds, we have further studied more pesticides such as flonicamid and butocarboxim. They showed high LOQs (50 and 150 ng/mL, respectively) in this methodology. The signal suppression by urine matrices were very strong for these compounds (matrix effect; −58.8% and −83.7%, respectively), so further cleanup steps such as solid-phase extraction [43] are needed to remove matrices and to improve their LOQ levels.

The linearity of calibration is determined by a correlation coefficient (r^2^) of the 1st order linear regression. The closer the r^2^ value is to 1, the better is the fit between signals and their concentrations (quantitative information). The 260 target pesticides showed r^2^ ≥ 0.988, meaning that target compounds had excellent quantitative properties with good linearity within their linear ranges.

#### 2.3.2. Accuracy and Precision

In bioanalytical methodology, the accuracy is expressed as the RE of an expected value, and its variation in repeatability (precision) is determined using the RSD. According to the Guidance for Industry: Bioanalytical Method Validation criteria, acceptable accuracy ranges are divided into two types by treated levels; First, from −20% to 20% with RSD ≤ 20% at LOQ level. Second, from −15% to 15% with RSD ≤ 15% at other higher concentrations [44]. The accuracy and precision were validated at four treated levels of 10 (LOQ), 50, 150, and 250 ng/mL during the intra- and inter-day tests. For the concentration of 10 ng/mL, RE ranges for 260 analytes were −18.4% to 19.5% with RSDs from 2.1% to 19.7% in the intra-day measurements and −18.2% to 17.4% with RSDs from 2.6% to 19.9% in inter-day (Table 4). For other concentrations (50, 150, and 250 ng/mL), RE ranges were −14.7% to 13.1% with RSDs from 0.6% to 14.9% in the intra-day and −11.5% to 14.9% with RSDs from 0.6% to 14.7% in inter-day. As a result, all the target compounds satisfied the accuracy and precision criteria. Although 260 pesticides were extracted together from a sample and analyzed simultaneously within only 15 min, the analytes did not lose their chemical properties or react with each other. In addition, the MRM mode of the tandem mass spectrometry exhibited excellent throughput abilities to select, detect, and quantify hundreds of pesticides. Therefore, biomonitoring for pesticide multiresidues in urine can be determined with high reliability using this analytical method.

#### 2.3.3. Recovery

The extraction efficiency of the preparation step is considered to be excellent when the recovery rate of a compound is close to 100%. Therefore, recovery can affect the determination of sensitivity for target compounds. Recovery and its variation (RSD) are also regarded as accuracy and precision parameters in many bioanalytical methods [5,45]. Generally, a recovery rate of 70%–120% with RSD ≤ 20% is an acceptable range for those parameters [39]. The recovery study was conducted at treated levels of 10, 50, and 250 ng/mL. The recovery ranges were 54.2%–113.9% (RSD; 0.5%–19.9%), 71.8%–106.1% (RSD; 0.6%–20.0%), and 68.5%–99.1% (RSD; 0.3%–11.9%) at 10, 50, and 250 ng/mL, respectively (Table 4). Most of the target pesticides satisfied the recovery range of 70%–120% with RSD ≤ 20% criteria. Only for the five compounds (chloridazon, chlorsulfuron, fluxapyroxad, milbemectin A4, and pretilachlor), lower recovery values (54.2%–69.3%) were observed in some concentrations but acceptable repeatability (RSD; 5.7%–17.1%).

From the recovery data, most of the pesticides showed high extraction efficiency by this bioanalytical method. In spite of the diverse chemical properties of the different pesticides, strong extraction/partitioning reagents make the pesticides maintain overall excellent recovery rates. Additionally, further cleanup steps were excluded to prevent the loss of target analytes.

Some compounds such as acetamiprid, cyprodinil, dicrotophos, imazalil, phosphamidon, and simazine showed higher recovery range (77.4%–109.9%) at 10 ng/mL than that in Cazorla-Reyes et al. (2011)’s results (60%–68%) in which analyzing 87 multiresidues using SPE (C18 cartridge) and LC-MS/MS [5]. It is considered that these compounds were adsorbed on C18 sorbent and some of them were not eluted.

**Figure 2:**
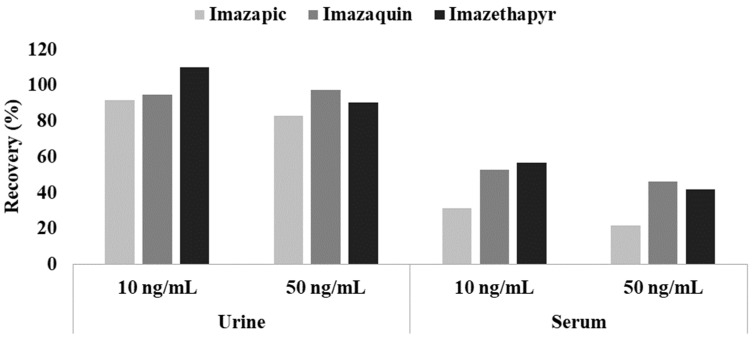
The recovery results for three imidazolinones in urine and serum samples.

For imidazolinones (imazapic, imazaquin, and imazethapyr), their recoveries were superior to those in our previous study using the same preparation in serum samples (Figure 2). Because imidazolinones are zwitterions, ion suppression is required using acidic buffers to improve extraction efficiencies in serum [35]. Urine, however, is generally acidic, so imidazolinones were fully extracted without the help of buffer reagents.

Except for the target 260 compounds, we have further verified recoveries for more compounds such as dithianon, 4-trifluoromethylnicotinic acid (TFNA), and N-(4-trifluoromethylnicotinoyl)-glycine (TFNG) (metabolites of flonicamid) in urine. They showed poor recovery rates in this preparation procedure method (35.2%–52.1% at 50 and 250 ng/mL) as well as preparation method B and C. Dithianon is easily hydrolyzed in alkaline but stable in acidic media, thus dSPE including primary-secondary amine (PSA) is not suitable for urine treatment [46]. TFNA and TFNG need lower pH conditions for ion suppression, thus strong acidic reagents such as formic acid are needed to adjust pH and increase their recovery rates as well as LOQ level.

#### 2.3.4. Matrix Effect

It has been reported that urine, as one of the most complex biological matrices, may affect ionization of target compounds in the ESI step of LC-MS/MS such that the intensity of the chromatogram could be enhanced or suppressed compared with that of non-matrix-based solutions [47,48]. Usually, the more concentrated is the urine, the more severe is the observed matrix effect [47]. Therefore, dilution of the sample is the common way to minimize the matrix effect and its mitigation between different samples [16,49].

We have compared the matrix effects using the same extraction sovent volume (400 μL) but different urine volumes (100 μL vs. 400 μL). The overall matrix effects of target pesticides were reduced in smaller urine volume (100 μL) (Figure S1). Therefore, 100 μL of urine was selected with a four times larger volume (400 μL) of organic solvent to reduce the matrix effect. The extract was subjected to partitioning with salts (MgSO_4_ and NaCl) for the exclusion of polar compounds such as urea, salts, glucuronides, or sulfates from the organic layer to reduce the matrix effect. The percentage of matrix effect was calculated using the following Equation (1):

(1) $$\mathrm{Matrix}\;\mathrm{effect},\;\%=\left(\frac{\mathrm{Calibration}\;\mathrm{slope}\;\mathrm{of}\;\mathrm{matrix}\;\mathrm{based}\;\mathrm{standard}}{\mathrm{Calibration}\;\mathrm{slope}\;\mathrm{of}\;\mathrm{solvent}\;\mathrm{based}\;\mathrm{standard}}-1\right)\times 100$$

Therefore, matrix effect of each compound can be expressed as percentage enhancement (>0%) or suppression (<0%). The farther away the percentage is from zero (0%), the larger is the matrix effect.

**Figure 3:**
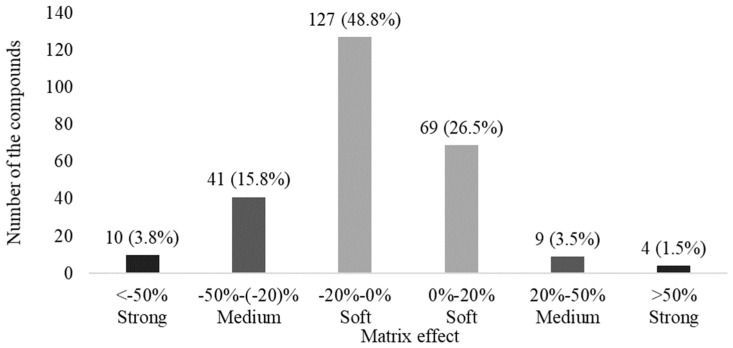
Summary of matrix effects for 260 pesticides in the final optimized method. Matrix effects are classified into soft effect (light grey bars, −20% to 0% and 0% to 20%), medium effect (grey bars, −50% to −20% and 20% to 50%), and strong effect (dark grey bars, <−50% and >50%).

To summarize and evaluate matrix effects, the results of the 260 pesticides in Table 4 were classified into three groups defined by six ranges including a soft effect (matrix effect within −20% to 0% or 0% to 20%), medium effect (−50% to −20% or 20% and 50%), and strong effect (below −50% or above 50%) based on the reports of Kmellár et al. [50] and Ferrer et al. [51] (Figure 3).

Most of the pesticides (196, 75.3% of total) were included in the soft effect group, in which 127 (48.8%) compounds fell between −20% and 0%, and 69 (26.5%) pesticides fell between 0% and 20%. Within the soft group, matrix effects are considered negligible on LC-MS/MS [51]. Therefore, it is possible to determine the concentration of real urine samples using solvent-only (matrix-free) standard solution rather than matrix-matched solution. The numbers of compounds in the medium and strong groups were 50 (19.3%) and 14 (5.3%), respectively. These groups were susceptible to interfering influences in urine, thus requiring matrix-matched calibration for correct quantitation.

**Figure 4:**
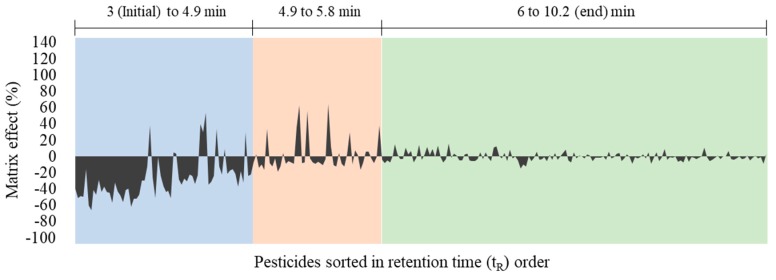
Distribution chart for 260 pesticides showing a pattern of matrix effects over retention time.

For verification of the matrix effect for each pesticide and correlation with retention time, a graph of matrix effects ordered by t_R_ is shown in Figure 4. From the initiation time of pesticide elution to around 4.9 min, a large number of pesticides showed the matrix effect below -20%. In contrast, during t_R_ of 4.9 to 5.8 min, signal suppression was reduced but some pesticides showed signal enhancement with matrix effects >20%. Matrix effects weakened after approximately 5.8 min through the end of the elution time. This result indicates that most of the polar urinary matrices co-eluted with target pesticides in the early stages of the analytical time (~5.8 min), causing considerable signal suppression/enhancement of target compounds.

### 2.4. Application

**Figure 5:**
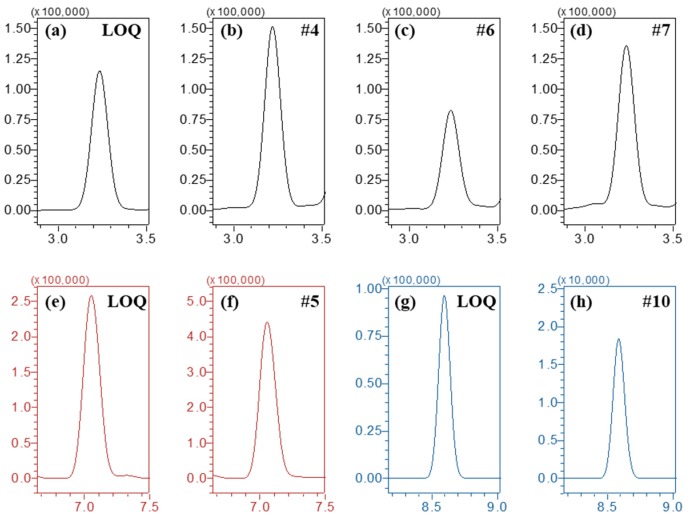
Chromatograms of (**a**–**d**) imidacloprid from QC (LOQ) and agricultural workers (#4, #6, and #7), (**e**,**f**) difenoconazole from QC (LOQ) and agricultural workers (#5), and (**g**,**h**) chlorfluazuron from QC (LOQ) and agricultural workers (#10). MRM transitions in the chromatograms were 255.8 > 209.0 (imidacloprid), 406.0 > 250.9 (difenoconazole), and 539.8 > 382.8 (chlorfluazuron), respectively.

Urine samples from agricultural workers were analyzed (*n* = 10, designated as #1 to #10) using the established method, and five samples (#4, #5, #6, #7, and #10) showed positive detections (Table 5 and Figure 5). Therefore, this bioanalytical method with tandem mass spectrometry is appropriate to determine pesticides in unknown urine samples.

## 3. Materials and Methods

### 3.1. Reagents

Reference pesticide standards (purity >98%) or stock solutions (1000 mg/L) were sourced from Sigma-Aldrich (St, Louis, MO, USA), Dr. Ehrenstorfer (Augsburg, Germany), Wako Pure Chemical Industries (Osaka, Japan), ChemService (West Chester, PA, USA), and Ultra Scientific (North Kingstown, RI, USA). Acetonitrile and methanol (HPLC grade) were purchased from Fisher Scientific (Seoul, the Republic of Korea). Sodium chloride (NaCl, 99.0%) was bought from Samchun (Gyeonggi-do, Korea). Ammonium formate (≥99.0%), formic acid (LC-MS grade), acetic acid (HOAc, ≥99.7%), sodium citrate tribasic dihydrate (Na_3_Citrate · 2H_2_O, ≥99.0%), sodium citrate dibasic sesquihydrate (Na_2_HCitr·1.5H_2_O, ≥99.0%), sodium acetate anhydrous (NaOAc, ≥99.0%), and magnesium sulfate anhydrous (MgSO_4_, ≥99.5%) were obtained from Sigma-Aldrich. Ceramic homogenizers (2 mm) were obtained from Ultra Scientific. Ultrapure water was prepared in house using LaboStar™ TWF UV 7 (Siemens, Lowell, MA, USA).

### 3.2. Urine Samples Collection

Blank urine samples were obtained from healthy volunteers. For the application of the established method, ten samples from male agricultural workers were collected in urine bags. The samples were stored at −70 °C until preparation and analysis. Urine sample collection was conducted under the permission of the Institutional Review Board (IRB) at Seoul National University, Seoul, the Republic of Korea (IRB No. 1604/002-007).

### 3.3. Preparation of Standard Solutions

Each reference standard was dissolved in acetonitrile, acetone, methanol, or water to prepare a 1000 mg/L stock solution. For some compounds (e.g., carbendazim) that are partially insoluble at this concentration, lower concentrations of stock solutions were prepared to make the compounds thoroughly soluble. These solutions were subjected to further dilution for use in MRM optimization on LC-MS/MS. To prepare four groups of intermediate mixed stock solutions at 10 mg/L, a portion of each stock solution was brought up with acetonitrile in a 25-mL volumetric flask. The aliquots of intermediates were again mixed to make a final mixed standard solution at 2.5 mg/L. This mixture was serial-diluted with acetonitrile for use in methodology validation steps. All the stock and standard solutions were stored at −20 °C until use.

### 3.4. LC-MS/MS Instrumental Conditions

LC-MS/MS analysis was carried out on a LCMS-8050 triple quadrupole mass spectrometer (Shimadzu, Kyoto, Japan) coupled to a Shimadzu Nexera X2 UHPLC system. For the mass spectrometer system, a heated ESI probe and positive/negative switching mode were used for target analyte ionization. Heating gas (air), drying gas (nitrogen), and nebulizing gas (nitrogen) flow rates were 10, 15, and 3 L/min, respectively. Argon gas was used for collision-induced dissociation (CID). The heat block, interface, and desolvation line (DL) temperatures were 400, 300, and 250 °C, respectively. To optimize MRM conditions, each target analyte at 0.1–1 mg/L was subjected to a Q3 full scan with mass to charge ratio (*m/z*) range of 50–500 or 100–1000. A precursor ion (e.g., [M + H]^+^) was selected according to its spectrum pattern, and more than 2 kinds of product ions were determined from the precursor ion using CID gas with variable collision energy (CE) voltages. Finally, two product ions with specific CEs were selected as quantifier and qualifier ions based on their selectivity and sensitivity. These optimized MRM conditions were scheduled according to the retention time of each compound, such that the MRM detection window was ± 0.5 min. Dwell times were adjusted to ≥ 2.0 ms based upon loop time (0.12 s) for maximizing data acquisition.

The UHPLC system comprised a solvent delivery module (LC-30AD), column oven (CTO-20A), autosampler (SIL-30AC), and degassing unit (DGU-20A5R). A Kinetex^®^ C18 column (100 × 2.1 mm, 2.6 µm, Phenomenex, Torrance, CA, USA) was used for analyte separation, and a SecurityGuard™ Ultra guard column (Phenomenex) was connected to the column to prevent contamination. The oven temperature was maintained at 40 °C. The total flow rate of the mobile phase was 0.2 mL/min. For the mobile phases, solvent A was 5 mM ammonium formate and 0.1% formic acid in water and B was 5 mM ammonium formate and 0.1% formic acid in methanol. For the gradient program, mobile phase B was initialized at 5%, and maintained for 0.5 min. The ratio of B was raised to 55% for 0.5 min, ramped to 95% for 7 min, held for 3 min, raised to 100% for 1 min, then dropped sharply to 5% for 0.1 min, and held for 2.9 min. The total analytical time was 15.0 min, and the injection volume was 4 µL. LabSolutions software (version 5.72) was used for multiresidue MRM data processing.

### 3.5. Preparation of Three Versions of QuEChERS

The optimization of urine treatment methods by comparing three different versions of QuEChERS modified from Original [29], AOAC [30], and EN QuEChERS [31] was performed as follows: Method A; 400 μL of acetonitrile, 40 mg of MgSO_4_, and 10 mg of NaCl. Method B; 1% HOAc in acetonitrile (400 μL), 40 mg of MgSO_4_, and 10 mg of NaOAc. Method C; 400 μL of acetonitrile, 40 mg of MgSO_4_, 10 mg of NaCl, 5 mg of Na_2_HCitr, and 10 mg of Na_3_Citrate. The extract from each method was centrifuged, and 200 µL of supernatant was collected and mixed with 50 µL of solvent (acetonitrile). The urine sample was equivalent to 200 µL per 1000 µL in the final extract. A portion of the sample (4 µL) was injected into the LC-MS/MS, and the recovery as well as relative peak intensity of the three methods were compared to optimize the final sample preparation.

### 3.6. The Final Optimized Method

Human urine (100 µL) was transferred to a 1.5-mL microcentrifuge tube, and 400 µL of acetonitrile and two ceramic homogenizers were added before being shaken with a Geno Grinder (1600 MiniG SPEX Sample Prep, Metuchen, NJ, USA) for 1 min at 1200 rpm. The sample was cooled in an ice bath and shaken again for 1 min at 1200 rpm after 40 mg of MgSO_4_ and 10 mg of NaCl were added. After shaking, the sample was centrifuged for 5 min at 16800g (13000 rpm) using a microcentrifuge (17TR, Hanil Science, Seoul, Korea). Then, 200 µL of the upper organic layer was transferred to a 2-mL amber glass vial, and acetonitrile (50 µL) was added for matrix-matching. Without further cleanup steps, the final extract (4 µL) was injected into the LC-MS/MS for analysis of multiresidue pesticides.

### 3.7. Analytical Method Validation

The limit of quantitation (LOQ, 10 ng/mL) was evaluated with signal to noise ratio (s/n) as well as RE and RSD of accuracy and precision results. The accuracy and precision tests were conducted on intra-day and inter-day conditions using a quality control (QC) sample (a sample with a known quantity of analyte [44]) with a calibration range from 10 to 250 ng/mL. The intra-day test was performed in one day by analyzing five QC samples of urine at 10, 50, 150, or 250 ng/mL, respectively. The inter-day test was carried out with a single QC sample of 10, 50, 150, or 250 ng/mL per day and repeated on 5 consecutive days. The accuracy and precision results were expressed with RE and RSD. For evaluation of the recovery, pesticides were spiked in blank urine before and after preparation (10, 50, and 250 ng/mL; *n* = 3). The result was calculated as a ratio of the pre-spiking sample’s response to the post-spiking sample’s response. The matrix effect of each target compound was evaluated by comparing a calibration slope of matrix-based standard and that of solvent-based (matrix-free) standard.

## 4. Conclusions

A sensitive, fast, and simultaneous methodology for 260 pesticides in urine was successfully developed utilizing LC-MS/MS. Scheduled MRM for each target pesticide was optimized with the high-throughput triple quadrupole mass spectrometer. As a result, an average of 17.3 pesticides could be detected in a minute, thus the total analysis time for 260 pesticides was within only 15 min in a sample. Tiny volumes of urine (100 μL) were used for sample preparation considering a realistic situation where a lot of urine cannot be collected from a pesticide poisoning victim or patient. To maximize extraction efficiency and minimize matrix effects, three versions of QuEChERS were compared, and the scaled-down QuEChERS procedure without dSPE cleanup was optimized for diverse chemical properties of the different pesticides. LOQs for target compounds were sufficiently low to detect pesticides in urinary samples. The final optimized analytical method for 260 pesticides was fully validated with the parameters of linearity of calibration, accuracy/precision, recovery, and matrix effect. The established method was successfully applied to determine pesticides in agricultural exposure samples. Therefore, the scaled-down QuEChERS method using LC-MS/MS can be a strong alternative to current analytical techniques.

## Figures and Tables

**Table 1:** Reagents used in three versions of the urine treatment and distribution of recovery results.

**Item**	**Method A**	**Method B**	**Method C**
**Reagents**			
Extraction Solvent	400 μL of acetonitrile	1% HOAc in acetonitrile (400 μL)	400 μL of acetonitrile
Extraction Reagent	MgSO_4_ (40 mg) NaCl (10 mg)	MgSO_4_ (40 mg) NaOAc (10 mg)	MgSO_4_ (40 mg) NaCl (10 mg) Na_3_Citrate (10 mg) Na_2_HCitr (5 mg)
**Recovery (%)**	**No. of Analytes (%)**	**No. of Analytes (%)**	**No. of Analytes (%)**
40–70 (RSD ≤ 20%)	2 (0.8%)	3 (1.2%)	0
70–120 (RSD ≤ 20%)	258 (99.2%)	256 (98.5%)	258 (99.2%)
70–120 (RSD > 20%)	0	0	1 (0.4%)
>120 (RSD ≤ 20%)	0	1 (0.4%)	0
n.d.^1^	0	0	1 (0.4%)
Sum	260 (100%)	260 (100%)	260 (100%)
Average Recovery	92.3%	87.3%	84.9%

^1^ Not determined.

**Table 2:** Distribution of relative peak intensity results (100 at solvent standard) for three versions of the urine treatment.

Relative Peak Intensity^1^	Method A		Method B		Method C	
	No. of Analytes (%)		No. of Analytes (%)		No. of Analytes (%)	
	10 ng/mL	250 ng/mL	10 ng/mL	250 ng/mL	10 ng/mL	250 ng/mL
<50	7 (2.7%)	6 (2.3%)	25 (9.6%)	21 (8.1%)	45 (17.3%)	44 (16.9%)
50–80	46 (17.7%)	43 (16.5%)	71 (27.3%)	73 (28.1%)	77 (29.6%)	78 (30.0%)
80–120	189 (72.7%)	194 (74.6%)	150 (57.7%)	157 (60.4%)	123 (47.3%)	126 (48.5%)
120–150	15 (5.8%)	15 (5.8%)	13 (5.0%)	8 (3.1%)	13 (5.0%)	9 (3.5%)
>150	3 (1.2%)	2 (0.8%)	1 (0.4%)	1 (0.4%)	1 (0.4%)	2 (0.8%)
n.d.^2^	0	0	0	0	1 (0.4%)	1 (0.4%)
Sum	260 (100%)	260 (100%)	260 (100%)	260 (100%)	260 (100%)	260 (100%)

^1^$\mathrm{Relative}\;\mathrm{peak}\;\mathrm{intensity}=\left(\frac{\mathrm{Peak}\;\mathrm{area}\;\mathrm{of}\;\mathrm{post}\;\mathrm{spiked}\;\mathrm{sample}}{\mathrm{Peak}\;\mathrm{area}\;\mathrm{of}\;\mathrm{solvent}\;\mathrm{standard}}\right)\times 100$ [40]; ^2^ Not determined.

**Table 3:** Retention time (t_R_), monoisotopic mass, and MRM transition of 260 target pesticides.

No.	Compound Name	t_R_ (min)	Mono-Isotopic Mass	Ionization Form of Precursor Ion	Precursor Ion > Product Ion (CE^1^, V)		Ratio^2^ (%)
					Quantifier	Qualifier	
1	Acetamiprid	3.26	222.1	[M + H]^+^	222.6 > 126.0 (−20)	222.6 > 56.1 (−15)	36.1
2	Alachlor	8.03	269.1	[M + H]^+^	269.7 > 238.0 (−11)	269.7 > 162.1 (−20)	101.9
3	Allidochlor	3.76	173.1	[M + H]^+^	173.9 > 98.1 (−14)	173.9 > 41.1 (−24)	53.5
4	Ametoctradin	7.22	275.2	[M + H]^+^	275.7 > 176.1 (−36)	275.7 > 149.1 (−37)	107.4
5	Ametryn	4.94	227.1	[M + H]^+^	227.6 > 186.0 (−19)	227.6 > 68.0 (−38)	55.5
6	Amisulbrom	7.49	465.0	[M + H]^+^	465.7 > 226.9 (−21)	465.7 > 148.0 (−51)	37.7
7	Anilofos	6.57	367.0	[M + H]^+^	367.5 > 125.0 (−31)	367.5 > 198.9 (−15)	69.0
8	Asulam	2.88	230.0	[M + H]^+^	231.0 > 155.9 (−11)	231.0 > 92.1 (−24)	64.1
9	Atrazine	4.66	215.1	[M + H]^+^	215.7 > 174.0 (−18)	215.7 > 104.0 (−28)	42.5
10	Azaconazole	4.71	299.0	[M + H]^+^	299.5 > 158.9 (−27)	299.5 > 230.9 (−17)	38.0
11	Azamethiphos	3.80	324.0	[M + H]^+^	324.5 > 183.0 (−16)	324.5 > 112.1 (−36)	86.6
12	Azimsulfuron	4.53	424.1	[M + H]^+^	424.6 > 182.0 (−19)	424.6 > 139.0 (−41)	29.5
13	Azinphos-methyl	4.95	317.0	[M + H]^+^	317.8 > 77.0 (−39)	317.8 > 125.0 (−20)	53.6
14	Azoxystrobin	5.03	403.1	[M + H]^+^	403.6 > 372.0 (−17)	403.6 > 344.0 (−25)	29.0
15	Bendiocarb	3.95	223.1	[M + H]^+^	223.6 > 109.0 (−18)	223.6 > 167.1 (−10)	48.4
16	Bensulfuron-methyl	4.85	410.1	[M + H]^+^	410.6 > 182.0 (−20)	410.6 > 149.0 (−20)	53.4
17	Bentazone	3.73	240.1	[M−H]^−^	238.9 > 132.0 (24)	238.9 > 197.1 (19)	59.6
18	Benthiavalicarb-isopropyl	5.59	381.2	[M + H]^+^	381.6 > 180.0 (−33)	381.6 > 116.1 (−21)	48.9
19	Benzobicyclon	5.45	446.0	[M + H]^+^	446.5 > 257.0 (−24)	446.5 > 229.0 (−36)	72.5
20	Benzoximate	7.01	363.1	[M + H]^+^	363.9 > 199.0 (−12)	363.9 > 105.1 (−26)	51.9
21	Boscalid	5.31	342.0	[M + H]^+^	342.6 > 307.0 (−21)	342.6 > 140.0 (−20)	31.0
22	Bromacil	4.02	260.0	[M + H]^+^	260.8 > 204.9 (−14)	260.8 > 187.9 (−28)	22.3
23	Bromobutide	6.06	311.1	[M + H]^+^	311.8 > 194.0 (−13)	311.8 > 91.1 (−45)	73.0
24	Bupirimate	6.06	316.2	[M + H]^+^	316.6 > 166.1 (−24)	316.6 > 210.1 (−24)	29.2
25	Buprofezin	7.86	305.2	[M + H]^+^	305.7 > 57.1 (−24)	305.7 > 200.9 (−15)	80.4
26	Butachlor	7.98	311.2	[M + H]^+^	312.1 > 238.1 (−13)	312.1 > 162.1 (−21)	36.6
27	Butafenacil	5.81	474.1	[M+NH_4_]^+^	491.6 > 331.0 (−25)	491.6 > 180.0 (−45)	76.6
28	Cadusafos	7.22	270.1	[M + H]^+^	270.6 > 158.9 (−17)	270.6 > 130.9 (−22)	111.3
29	Carbaryl	4.14	201.1	[M + H]^+^	201.8 > 145.1 (−11)	201.8 > 127.1 (−26)	73.3
30	Carbendazim	3.11	191.1	[M + H]^+^	191.6 > 159.8 (−24)	191.6 > 132.1 (−29)	42.2
31	Carbofuran	3.98	221.1	[M + H]^+^	221.6 > 123.0 (−21)	221.6 > 165.1 (−11)	70.6
32	Carbophenothion	8.43	342.0	[M + H]^+^	342.8 > 157.0 (−13)	342.8 > 45.0 (−37)	34.3
33	Carboxin	4.17	235.1	[M + H]^+^	235.6 > 143.0 (−15)	235.6 > 87.0 (−25)	36.1
34	Carpropamid	6.66	333.0	[M + H]^+^	333.6 > 139.0 (−21)	333.6 > 103.1 (−42)	84.6
35	Cartap	6.51	237.1	[M + H]^+^	238.0 > 150.0 (−14)	238.0 > 73.0 (−28)	72.8
36	Chlorantraniliprole	4.82	483.0	[M + H]^+^	483.5 > 452.8 (−18)	483.5 > 285.9 (−16)	68.2
37	Chlorfenvinphos	6.70	358.0	[M + H]^+^	358.5 > 99.0 (−30)	358.5 > 169.9 (−40)	114.7
38	Chlorfluazuron	8.74	539.0	[M + H]^+^	539.8 > 382.8 (−23)	539.8 > 158.0 (−20)	43.2
39	Chloridazon	3.35	221.0	[M + H]^+^	221.5 > 104.1 (−22)	221.5 > 77.0 (−35)	91.1
40	Chlorotoluron	4.44	212.1	[M + H]^+^	212.7 > 72.1 (−22)	212.7 > 46.2 (−16)	52.7
41	Chlorsulfuron	4.05	357.0	[M + H]^+^	358.0 > 141.1 (−20)	358.0 > 167.1 (−19)	94.7
42	Chromafenozide	5.87	394.2	[M + H]^+^	394.8 > 175.1 (−19)	394.8 > 147.0 (−44)	18.2
43	Cinmethylin	7.95	274.2	[M+NH_4_]^+^	292.1 > 105.0 (−23)	292.1 > 153.0 (−11)	9.7
44	Clofentezine	7.05	302.0	[M + H]^+^	303.0 > 138.0 (−15)	303.0 > 102.1 (−35)	84.9
45	Clomazone	5.02	239.1	[M + H]^+^	239.6 > 125.0 (−20)	239.6 > 89.1 (−49)	20.8
46	Clothianidin	3.19	249.0	[M + H]^+^	250.0 > 169.0 (−13)	250.0 > 132.0 (−18)	93.8
47	Cyanazine	3.78	240.1	[M + H]^+^	240.8 > 214.1 (−17)	240.8 > 104.0 (−30)	28.0
48	Cyazofamid	6.01	324.0	[M + H]^+^	325.0 > 108.0 (−13)	325.0 > 44.1 (−31)	20.8
49	Cyclosulfamuron	5.76	421.1	[M + H]^+^	421.6 > 261.0 (−19)	421.6 > 218.0 (−26)	77.4
50	Cymoxanil	3.41	198.1	[M + H]^+^	198.9 > 128.1 (−10)	198.9 > 111.1 (−18)	80.8
51-1	Cyproconazole_1	5.55	291.1	[M + H]^+^	291.8 > 70.1 (−21)	291.8 > 125.0 (−31)	50.8
51-2	Cyproconazole_2	5.77	291.1	[M + H]^+^	291.8 > 70.1 (−21)	291.8 > 125.0 (−31)	77.8
52	Cyprodinil	6.42	225.1	[M + H]^+^	225.6 > 93.1 (−34)	225.6 > 77.1 (−45)	56.4
53	Daimuron	5.61	268.2	[M + H]^+^	269.0 > 151.0 (−20)	269.0 > 91.0 (−50)	98.8
54	Diazinon	6.77	304.1	[M + H]^+^	305.0 > 169.0 (−25)	305.0 > 153.0 (−25)	73.6
55	Dicrotophos	3.08	237.1	[M + H]^+^	238.0 > 72.0 (−30)	238.0 > 112.1 (−15)	89.0
56	Diethofencarb	5.14	267.1	[M + H]^+^	268.0 > 124.0 (−35)	268.0 > 152.0 (−25)	69.9
57	Difenoconazole	7.14	405.1	[M + H]^+^	406.0 > 250.9 (−30)	406.0 > 188.0 (−46)	25.0
58	Diflubenzuron	6.18	310.0	[M + H]^+^	311.0 > 158.0 (−14)	311.0 > 141.0 (−30)	115.2
59	Diflufenican	7.37	394.1	[M + H]^+^	395.0 > 265.9 (−25)	395.0 > 246.0 (−35)	17.5
60	Dimethachlor	4.85	255.1	[M + H]^+^	256.0 > 224.0 (−20)	256.0 > 148.1 (−30)	100.9
61	Dimethametryn	6.22	255.2	[M + H]^+^	256.0 > 186.0 (−25)	256.0 > 68.0 (−46)	31.5
62	Dimethenamid	5.35	275.1	[M + H]^+^	276.0 > 244.0 (−20)	276.0 > 168.1 (−28)	48.3
63	Dimethoate	3.30	229.0	[M + H]^+^	230.0 > 198.9 (−11)	230.0 > 125.0 (−22)	112.5
64-1	Dimethomorph_1	5.19	387.1	[M + H]^+^	388.0 > 300.9 (−25)	388.0 > 165.0 (−35)	79.8
64-2	Dimethomorph_2	5.45	387.1	[M + H]^+^	388.0 > 300.9 (−25)	388.0 > 165.0 (−35)	77.7
65	Diniconazole	7.09	325.1	[M + H]^+^	326.0 > 70.1 (−25)	326.0 > 158.9 (−33)	13.1
66	Diphenamid	4.82	239.1	[M + H]^+^	240.0 > 134.1 (−25)	240.0 > 167.1 (−22)	20.4
67	Diuron	4.72	232.0	[M + H]^+^	233.0 > 72.0 (−25)	233.0 > 46.1 (−17)	51.5
68	Edifenphos	6.57	310.0	[M + H]^+^	311.0 > 109.0 (−40)	311.0 > 282.9 (−18)	71.3
69	Emamectin B1a	7.91	885.5	[M + H]^+^	886.4 > 158.1 (−39)	886.4 > 82.2 (−55)	17.4
70	Emamectin B1b	7.60	871.5	[M + H]^+^	872.3 > 158.1 (−36)	872.3 > 82.1 (−55)	17.4
71	EPN	7.27	323.0	[M + H]^+^	324.0 > 295.9 (−14)	324.0 > 156.9 (−22)	136.6
72	Epoxiconazole	6.00	329.1	[M + H]^+^	330.0 > 121.0 (−21)	330.0 > 101.1 (−49)	65.1
73	Ethaboxam (EBX)	4.29	320.1	[M + H]^+^	320.6 > 183.0 (−21)	320.6 > 200.0 (−25)	21.4
74	Ethametsulfuron-methyl	4.16	410.1	[M + H]^+^	410.6 > 196.0 (−18)	410.6 > 168.0 (−30)	76.8
75	Ethiofencarb	4.30	225.1	[M + H]^+^	225.6 > 107.1 (−16)	225.6 > 77.0 (−45)	41.6
76	Ethion	8.06	384.0	[M + H]^+^	384.6 > 198.9 (−11)	384.6 > 143.0 (−24)	108.9
77	Ethoprophos	6.04	242.1	[M + H]^+^	242.6 > 97.0 (−32)	242.6 > 130.9 (−20)	82.6
78	Ethoxyquin	5.08	217.1	[M + H]^+^	218.0 > 174.1 (−27)	218.0 > 148.0 (−22)	71.9
79	Ethoxysulfuron	5.52	398.1	[M + H]^+^	398.7 > 260.9 (−16)	398.7 > 218.0 (−25)	76.1
80	Etoxazole	8.48	359.2	[M + H]^+^	359.6 > 141.0 (−29)	359.6 > 113.0 (−55)	41.1
81	Etrimfos	6.65	292.1	[M + H]^+^	292.6 > 125.0 (−25)	292.6 > 265.0 (−17)	104.7
82	Fenamidone	5.25	311.1	[M + H]^+^	311.7 > 92.1 (−24)	311.7 > 236.1 (−15)	90.3
83	Fenamiphos	6.22	303.1	[M + H]^+^	303.6 > 217.0 (−23)	303.6 > 201.9 (−35)	50.4
84	Fenazaquin	9.08	306.2	[M + H]^+^	306.7 > 161.2 (−17)	306.7 > 57.2 (−26)	109.4
85	Fenbuconazole	6.06	336.1	[M + H]^+^	336.9 > 125.0 (−31)	336.9 > 70.1 (−21)	77.7
86	Fenhexamid	5.86	301.1	[M + H]^+^	302.0 > 97.2 (−24)	302.0 > 55.1 (−41)	68.2
87	Fenobucarb (BPMC)	5.12	207.1	[M + H]^+^	207.9 > 95.0 (−16)	207.9 > 77.0 (−39)	30.0
88	Fenothiocarb	6.35	253.1	[M + H]^+^	253.7 > 72.1 (−23)	253.7 > 160.0 (−10)	18.3
89	Fenoxanil	6.24	328.1	[M + H]^+^	329.0 > 302.0 (−12)	329.0 > 86.1 (−23)	62.7
90	Fenoxycarb	6.29	301.1	[M + H]^+^	302.1 > 88.1 (−21)	302.1 > 116.1 (−12)	58.3
91	Fenthion	6.61	278.0	[M + H]^+^	279.0 > 246.9 (−13)	279.0 > 169.0 (−18)	118.1
92	Ferimzone	4.73	254.2	[M + H]^+^	254.6 > 91.1 (−32)	254.6 > 132.1 (−20)	118.8
93	Fipronil	6.22	435.9	[M−H]^−^	434.6 > 330.0 (16)	434.6 > 250.0 (26)	37.9
94	Fluacrypyrim	7.24	426.1	[M + H]^+^	426.9 > 145.0 (−26)	426.9 > 205.0 (−11)	40.6
95	Fluazinam	7.97	464.0	[M−H]^−^	462.7 > 416.0 (18)	462.7 > 398.0 (16)	44.1
96	Flucetosulfuron	4.94	487.1	[M + H]^+^	487.8 > 156.0 (−20)	487.8 > 273.0 (−26)	44.6
97	Flufenacet	5.96	363.1	[M + H]^+^	363.6 > 152.1 (−20)	363.6 > 194.1 (−11)	46.7
98	Flufenoxuron	8.40	488.0	[M + H]^+^	488.8 > 158.0 (−20)	488.8 > 141.0 (−46)	92.7
99	Fluopicolide	5.54	382.0	[M + H]^+^	382.5 > 172.9 (−23)	382.5 > 145.0 (−48)	58.3
100	Fluopyram	5.81	396.0	[M + H]^+^	396.5 > 145.1 (−53)	396.5 > 173.0 (−28)	88.3
101	Flusilazole	6.21	315.1	[M + H]^+^	315.6 > 247.0 (−18)	315.6 > 165.0 (−26)	101.8
102	Flusulfamide	6.84	413.9	[M−H]^−^	412.6 > 171.1 (36)	412.6 > 349.0 (26)	35.7
103	Flutolanil	5.45	323.1	[M + H]^+^	323.6 > 242.0 (−26)	323.6 > 262.0 (−19)	84.5
104	Fluxapyroxad	5.47	381.1	[M + H]^+^	381.5 > 362.0 (−15)	381.5 > 342.0 (−21)	95.1
105	Fonofos	6.70	246.0	[M + H]^+^	247.0 > 109.0 (−19)	247.0 > 137.0 (−12)	47.5
106	Forchlorfenuron	4.62	247.1	[M−H]^−^	245.9 > 127.1 (11)	245.9 > 91.0 (26)	12.6
107	Fosthiazate	4.30	283.0	[M + H]^+^	283.5 > 104.0 (−24)	283.5 > 227.9 (−10)	51.4
108	Furathiocarb	7.76	382.2	[M + H]^+^	382.6 > 195.0 (−19)	382.6 > 252.0 (−13)	46.7
109	Halosulfuron-methyl	5.82	434.0	[M + H]^+^	434.8 > 182.0 (−22)	434.8 > 139.0 (−43)	34.8
110	Haloxyfop-R-Methyl	7.25	375.0	[M + H]^+^	375.6 > 316.0 (−18)	375.6 > 91.1 (−32)	56.2
111	Hexaconazole	6.81	313.1	[M + H]^+^	313.9 > 70.1 (−21)	313.9 > 158.9 (−32)	25.3
112	Hexaflumuron	7.38	460.0	[M−H]^−^	458.8 > 438.9 (11)	458.8 > 175.1 (34)	14.6
113	Hexazinone	4.00	252.2	[M + H]^+^	252.7 > 170.8 (−20)	252.7 > 71.1 (−32)	56.7
114	Imazalil	4.26	296.0	[M + H]^+^	296.6 > 158.9 (−23)	296.6 > 200.9 (−18)	50.8
115	Imazapic	3.41	275.1	[M + H]^+^	275.6 > 163.0 (−26)	275.6 > 216.0 (−22)	68.0
116	Imazaquin	3.92	311.1	[M + H]^+^	311.6 > 267.0 (−21)	311.6 > 199.0 (−28)	100.9
117	Imazethapyr	3.71	289.1	[M + H]^+^	289.6 > 177.1 (−27)	289.6 > 245.1 (−21)	123.1
118	Imibenconazole	7.91	410.0	[M + H]^+^	410.7 > 125.0 (−30)	410.7 > 171.0 (−20)	16.0
119	Imicyafos	3.67	304.1	[M + H]^+^	304.5 > 201.0 (−22)	304.5 > 235.0 (−18)	16.1
120	Imidacloprid	3.15	255.1	[M + H]^+^	255.8 > 209.0 (−16)	255.8 > 175.1 (−20)	93.0
121	Indoxacarb	7.26	527.1	[M + H]^+^	527.9 > 203.0 (−40)	527.9 > 150.0 (−24)	65.9
122	Iprobenfos	6.43	288.1	[M + H]^+^	288.6 > 91.1 (−29)	288.6 > 205.0 (−11)	34.9
123	Iprovalicarb	5.88	320.2	[M + H]^+^	320.8 > 119.0 (−20)	320.8 > 203.1 (−10)	19.6
124	Isazofos	5.75	313.0	[M + H]^+^	313.7 > 162.0 (−16)	313.7 > 97.0 (−34)	35.6
125	Isoprocarb	4.53	193.1	[M + H]^+^	193.9 > 95.1 (−15)	193.9 > 77.1 (−38)	37.7
126	Isoprothiolane	5.54	290.1	[M + H]^+^	290.8 > 188.9 (−22)	290.8 > 231.0 (−12)	57.1
127	Isoproturon	4.65	206.1	[M + H]^+^	206.7 > 72.1 (−21)	206.7 > 46.1 (−18)	52.4
128	Isopyrazam	7.18	359.2	[M + H]^+^	359.7 > 244.1 (−23)	359.7 > 320.1 (−21)	69.9
129	Isoxathion	6.93	313.1	[M + H]^+^	313.7 > 105.2 (−15)	313.7 > 97.0 (−35)	68.7
130	Kresoxim-methyl	6.47	313.1	[M + H]^+^	314.1 > 222.0 (−14)	314.1 > 267.0 (−8)	67.3
131	Linuron	5.22	248.0	[M + H]^+^	249.0 > 160.0 (−18)	249.0 > 182.0 (−15)	68.3
132	Mandipropamid	5.34	411.1	[M + H]^+^	411.9 > 328.0 (−16)	411.9 > 125.0 (−34)	79.7
133	Mecarbam	5.96	329.1	[M + H]^+^	330.0 > 226.9 (−9)	330.0 > 97.0 (−38)	106.5
134	Mefenacet	5.72	298.1	[M + H]^+^	298.7 > 148.0 (−14)	298.7 > 120.1 (−24)	90.3
135	Mefenpyr-diethyl	6.81	372.1	[M + H]^+^	372.8 > 327.0 (−16)	372.8 > 160.0 (−33)	32.1
136	Mepronil	5.55	269.1	[M + H]^+^	269.6 > 119.0 (−26)	269.6 > 91.1 (−41)	100.4
137	Metalaxyl	4.61	279.1	[M + H]^+^	279.6 > 220.1 (−13)	279.6 > 192.1 (−17)	61.7
138	Metamifop	7.65	440.1	[M + H]^+^	440.9 > 288.0 (−20)	440.9 > 123.1 (−28)	43.0
139	Metazosulfuron	5.14	475.1	[M + H]^+^	475.9 > 182.0 (−20)	475.9 > 294.9 (−18)	49.5
140	Metconazole	6.82	319.2	[M + H]^+^	320.1 > 70.0 (−24)	320.1 > 125.0 (−39)	18.5
141	Methabenzthiazuron	4.59	221.1	[M + H]^+^	221.5 > 165.0 (−17)	221.5 > 150.0 (−31)	43.5
142	Methidathion	4.84	302.0	[M + H]^+^	302.9 > 145.0 (−10)	302.9 > 85.1 (−22)	128.1
143	Methiocarb	5.28	225.1	[M + H]^+^	225.8 > 121.1 (−20)	225.8 > 169.0 (−10)	63.1
144	Methomyl	3.02	162.0	[M + H]^+^	163.0 > 88.0 (−10)	163.0 > 106.1 (−11)	65.1
145	Methoxyfenozide	5.57	368.2	[M + H]^+^	369.0 > 149.0 (−21)	369.0 > 313.1 (−8)	18.4
146	Metobromuron	4.51	258.0	[M + H]^+^	258.5 > 169.9 (−19)	258.5 > 148.0 (−16)	59.3
147	Metolachlor	6.16	283.1	[M + H]^+^	283.6 > 251.9 (−17)	283.6 > 176.1 (−25)	63.5
148	Metolcarb	3.80	165.1	[M + H]^+^	165.9 > 109.1 (−12)	165.9 > 94.1 (−30)	29.1
149	Metominostrobin	4.75	284.1	[M + H]^+^	284.6 > 196.0 (−18)	284.6 > 194.0 (−21)	97.0
150	Metrafenone	7.02	408.1	[M + H]^+^	408.9 > 209.0 (−15)	408.9 > 226.9 (−22)	80.3
151−1	Mevinphos_1	3.25	224.0	[M + H]^+^	224.7 > 127.0 (−16)	224.7 > 193.0 (−9)	55.5
151-2	Mevinphos_2	3.44	224.0	[M + H]^+^	224.7 > 127.0 (−16)	224.7 > 193.0 (−9)	33.8
152	Milbemectin A4	9.49	542.3	[M + H−H_2_O]^+^	525.0 > 109.2 (−27)	525.0 > 507.2 (−13)	82.9
153	Molinate	6.32	187.1	[M+NH_4_]^+^	204.6 > 145.1 (−15)	204.6 > 115.0 (−26)	52.5
154	Monocrotophos	3.03	223.1	[M + H]^+^	223.6 > 127.0 (−15)	223.6 > 193.0 (−8)	62.1
155	Myclobutanil	5.58	288.1	[M + H]^+^	289.1 > 70.1 (−21)	289.1 > 125.0 (−32)	54.5
156	Napropamide	6.03	271.2	[M + H]^+^	271.7 > 171.1 (−19)	271.7 > 129.1 (−16)	96.7
157	Nicosulfuron	3.77	410.1	[M + H]^+^	410.9 > 182.0 (−19)	410.9 > 213.0 (−17)	51.1
158	Nitenpyram	2.94	270.1	[M + H]^+^	270.6 > 225.0 (−12)	270.6 > 126.0 (−26)	106.6
159	Nuarimol	5.16	314.1	[M + H]^+^	315.0 > 252.0 (−22)	315.0 > 81.1 (−30)	50.6
160	Ofurace	3.93	281.1	[M + H]^+^	281.6 > 254.1 (−12)	281.6 > 160.1 (−24)	128.6
161	Omethoate	2.87	213.0	[M + H]^+^	213.5 > 125.0 (−21)	213.5 > 183.0 (−11)	62.6
162	Orysastrobin	5.51	391.2	[M + H]^+^	392.1 > 205.0 (−15)	392.1 > 116.1 (−28)	94.8
163	Oxadiazon	7.99	344.1	[M + H]^+^	344.9 > 303.0 (−14)	344.9 > 219.9 (−19)	99.4
164	Oxadixyl	3.61	278.1	[M + H]^+^	278.6 > 219.1 (−12)	278.6 > 132.1 (−29)	62.8
165	Oxamyl	2.92	219.1	[M+NH_4_]^+^	236.8 > 90.1 (−8)	236.8 > 56.0 (−45)	10.7
166	Oxydemeton-methyl	2.92	246.0	[M + H]^+^	246.5 > 169.0 (−13)	246.5 > 109.0 (−27)	82.1
167	Paclobutrazol	5.45	293.1	[M + H]^+^	294.1 > 70.1 (−21)	294.1 > 125.1 (−38)	17.9
168	Pebulate	7.17	203.1	[M + H]^+^	204.1 > 128.1 (−12)	204.1 > 57.1 (−17)	97.7
169	Penconazole	6.50	283.1	[M + H]^+^	284.0 > 70.1 (−16)	284.0 > 159.0 (−30)	98.8
170	Pendimethalin	8.30	281.1	[M + H]^+^	282.1 > 212.0 (−12)	282.1 > 194.0 (−18)	15.8
171	Penoxsulam	4.08	483.1	[M + H]^+^	483.9 > 195.0 (−28)	483.9 > 164.0 (−34)	22.5
172	Penthiopyrad	6.48	359.1	[M + H]^+^	359.8 > 276.0 (−15)	359.8 > 177.0 (−34)	133.4
173	Phenmedipham	4.81	300.1	[M+NH_4_]^+^	318.1 > 136.0 (−24)	318.1 > 168.0 (−14)	78.8
174	Phenthoate	6.40	320.0	[M + H]^+^	321.0 > 79.0 (−43)	321.0 > 247.0 (−12)	81.7
175	Phosalone	6.92	367.0	[M + H]^+^	367.7 > 182.0 (−17)	367.7 > 111.0 (−40)	68.8
176	Phosmet	4.95	317.0	[M + H]^+^	317.8 > 160.0 (−16)	317.8 > 77.0 (−54)	29.9
177	Phosphamidon	3.70	299.1	[M + H]^+^	300.0 > 174.0 (−14)	300.0 > 127.0 (−30)	60.1
178	Phoxim	6.90	298.1	[M + H]^+^	298.5 > 129.0 (−11)	298.5 > 77.0 (−30)	231.1
179	Picolinafen	7.93	376.1	[M + H]^+^	376.9 > 237.9 (−27)	376.9 > 358.9 (−20)	28.0
180	Picoxystrobin	6.31	367.1	[M + H]^+^	367.9 > 145.0 (−21)	367.9 > 205.1 (−9)	44.5
181	Piperophos	7.29	353.1	[M + H]^+^	353.7 > 170.9 (−23)	353.7 > 255.0 (−14)	50.6
182	Pirimicarb	4.05	238.1	[M + H]^+^	238.8 > 72.1 (−23)	238.8 > 182.1 (−16)	39.3
183	Pirimiphos-ethyl	7.94	333.1	[M + H]^+^	333.6 > 198.1 (−23)	333.6 > 182.1 (−23)	44.4
184	Pirimiphos-methyl	6.97	305.1	[M + H]^+^	305.7 > 108.0 (−31)	305.7 > 164.1 (−22)	90.1
185	Pretilachlor	7.49	311.2	[M + H]^+^	312.1 > 252.0 (−17)	312.1 > 176.1 (−29)	29.6
186	Probenazole	3.76	223.0	[M + H]^+^	224.0 > 41.1 (−22)	224.0 > 39.1 (−45)	44.2
187	Prochloraz	6.81	375.0	[M + H]^+^	375.8 > 308.0 (−13)	375.8 > 70.1 (−26)	43.2
188	Promecarb	5.46	207.1	[M + H]^+^	208.1 > 109.0 (−16)	208.1 > 151.1 (−10)	30.4
189	Prometryn	5.63	241.1	[M + H]^+^	241.6 > 158.0 (−23)	241.6 > 200.1 (−18)	47.8
190	Propachlor	4.65	211.1	[M + H]^+^	211.7 > 170.0 (−15)	211.7 > 94.1 (−27)	61.1
191	Propazine	5.31	229.1	[M + H]^+^	229.7 > 146.0 (−23)	229.7 > 188.0 (−17)	83.2
192	Propiconazole	6.70	341.1	[M + H]^+^	342.0 > 158.9 (−28)	342.0 > 69.2 (−21)	45.3
193	Propisochlor	6.64	283.1	[M + H]^+^	283.9 > 224.0 (−11)	283.9 > 43.1 (−25)	107.7
194	Propoxur	3.95	209.1	[M + H]^+^	209.8 > 111.0 (−14)	209.8 > 93.0 (−25)	59.8
195	Prothiofos	9.13	344.0	[M + H]^+^	344.9 > 240.7 (−20)	344.9 > 268.8 (−12)	43.7
196	Pyraclofos	6.91	360.0	[M + H]^+^	360.5 > 256.9 (−23)	360.5 > 138.0 (−40)	42.2
197	Pyraclostrobin	6.85	387.1	[M + H]^+^	388.0 > 163.1 (−25)	388.0 > 194.1 (−13)	83.5
198	Pyrazolynate	7.02	438.0	[M + H]^+^	438.6 > 91.1 (−37)	438.6 > 172.9 (−20)	76.2
199	Pyrazophos	7.00	373.1	[M + H]^+^	373.5 > 222.0 (−21)	373.5 > 194.0 (−32)	143.2
200	Pyrazoxyfen	6.62	402.1	[M + H]^+^	402.9 > 91.1 (−40)	402.9 > 105.1 (−21)	64.6
201	Pyribenzoxim	7.87	609.2	[M+Na]^+^	631.8 > 488.1 (−21)	631.8 > 180.1 (−40)	44.6
202	Pyributicarb	8.13	330.1	[M + H]^+^	330.6 > 181.0 (−16)	330.6 > 108.1 (−28)	108.6
203	Pyridaben	9.00	364.1	[M + H]^+^	364.6 > 147.1 (−25)	364.6 > 309.0 (−14)	75.7
204	Pyridalyl	10.07	489.0	[M + H]^+^	489.8 > 183.0 (−18)	489.8 > 109.0 (−28)	310.6
205	Pyridaphenthion	5.65	340.1	[M + H]^+^	340.5 > 189.0 (−21)	340.5 > 205.0 (−22)	57.7
206	Pyridate	9.36	378.1	[M + H]^+^	378.8 > 207.0 (−21)	378.8 > 351.1 (−10)	19.9
207-1	Pyrifenox_1	5.65	294.0	[M + H]^+^	294.5 > 93.1 (−22)	294.5 > 67.1 (−55)	6.2
207-2	Pyrifenox_2	5.94	294.0	[M + H]^+^	294.5 > 93.1 (−22)	294.5 > 67.1 (−55)	7.7
208	Pyriminobac-methyl E	5.40	361.1	[M + H]^+^	361.6 > 330.0 (−14)	361.6 > 284.0 (−30)	25.6
209	Pyriminobac-methyl Z	4.98	361.1	[M + H]^+^	361.6 > 330.0 (−15)	361.6 > 244.0 (−26)	10.7
210	Pyrimisulfan	4.69	419.1	[M + H]^+^	419.5 > 370.0 (−19)	419.5 > 255.0 (−28)	87.9
211	Pyriproxyfen	8.11	321.1	[M + H]^+^	321.6 > 96.1 (−16)	321.6 > 78.0 (−53)	49.1
212	Pyroquilon	3.93	173.1	[M + H]^+^	173.8 > 117.1 (−31)	173.8 > 132.1 (−22)	88.0
213	Quinoclamine	3.89	207.0	[M + H]^+^	208.0 > 89.0 (−39)	208.0 > 77.0 (−38)	110.3
214	Rimsulfuron	4.05	431.1	[M + H]^+^	431.5 > 182.0 (−22)	431.5 > 325.0 (−16)	51.5
215	Saflufenacil	4.90	500.0	[M + H]^+^	500.8 > 197.9 (−45)	500.8 > 348.9 (−29)	88.1
216	Sethoxydim	7.87	327.2	[M + H]^+^	327.6 > 178.0 (−20)	327.6 > 282.1 (−12)	45.6
217	Simazine	4.08	201.1	[M + H]^+^	201.9 > 104.0 (−26)	201.9 > 124.1 (−20)	70.2
218	Simeconazole	5.93	293.1	[M + H]^+^	294.1 > 70.1 (−21)	294.1 > 135.0 (−21)	24.5
219	Simetryn	4.31	213.1	[M + H]^+^	213.6 > 68.0 (−36)	213.6 > 124.1 (−20)	49.3
220	Spinetoram (XDE-175-J)	7.30	747.5	[M + H]^+^	748.1 > 142.1 (−32)	748.1 > 98.1 (−55)	16.0
221	Spinosyn A	6.79	731.5	[M + H]^+^	732.0 > 142.1 (−30)	732.0 > 98.1 (−54)	17.5
222	Spinosyn D	7.23	745.5	[M + H]^+^	746.3 > 142.1 (−31)	746.3 > 98.1 (−55)	21.1
223	Spirodiclofen	8.69	410.1	[M + H]^+^	410.9 > 71.1 (−21)	410.9 > 313.0 (−13)	74.5
224	Sulfoxaflor	3.32	277.0	[M + H]^+^	278.0 > 174.0 (−12)	278.0 > 154.0 (−29)	76.4
225	Sulprofos	8.30	322.0	[M + H]^+^	322.5 > 218.9 (−16)	322.5 > 155.0 (−24)	62.3
226	TCMTB	5.24	238.0	[M + H]^+^	238.8 > 180.0 (−12)	238.8 > 136.1 (−26)	70.8
227	Tebuconazole	6.53	307.1	[M + H]^+^	308.1 > 70.1 (−22)	308.1 > 125.1 (−40)	14.5
228	Tebufenozide	6.32	352.2	[M + H]^+^	353.2 > 133.1 (−20)	353.2 > 297.1 (−10)	22.0
229	Tebupirimfos	7.96	318.1	[M + H]^+^	318.6 > 277.0 (−15)	318.6 > 153.1 (−30)	117.9
230	Terbuthylazine	5.44	229.1	[M + H]^+^	229.7 > 174.0 (−17)	229.7 > 96.1 (−26)	21.2
231	Terbutryn	5.73	241.1	[M + H]^+^	241.6 > 186.1 (−19)	241.6 > 91.0 (−27)	15.4
232	Tetrachlorvinphos	6.33	365.9	[M + H]^+^	366.7 > 127.0 (−14)	366.7 > 205.9 (−38)	68.7
233	Tetraconazole	5.91	371.0	[M + H]^+^	371.8 > 159.0 (−30)	371.8 > 70.1 (−23)	41.2
234	Thenylchlor	5.96	323.1	[M + H]^+^	323.8 > 127.0 (−15)	323.8 > 53.0 (−55)	20.4
235	Thiabendazole	3.30	201.0	[M + H]^+^	201.5 > 175.0 (−24)	201.5 > 131.1 (−31)	128.1
236	Thiacloprid	3.38	252.0	[M + H]^+^	252.6 > 126.0 (−21)	252.6 > 99.0 (−43)	20.6
237	Thiazopyr	6.55	396.1	[M + H]^+^	396.6 > 377.0 (−23)	396.6 > 334.9 (−29)	55.2
238	Thidiazuron	3.91	220.0	[M−H]^−^	218.9 > 100.0 (9)	218.9 > 71.0 (32)	37.2
239	Thifensulfuron-methyl	3.75	387.0	[M + H]^+^	387.8 > 167.1 (−17)	387.8 > 204.9 (−27)	22.4
240	Thiobencarb	7.04	257.1	[M + H]^+^	257.8 > 125.0 (−20)	257.8 > 89.0 (−48)	21.4
241	Thiodicarb	4.20	354.0	[M + H]^+^	355.0 > 88.0 (−21)	355.0 > 108.0 (−15)	49.3
242	Thiophanate-methyl	3.82	342.1	[M + H]^+^	342.8 > 151.0 (−20)	342.8 > 310.9 (−11)	14.5
243	Tolfenpyrad	7.93	383.1	[M + H]^+^	383.7 > 197.0 (−25)	383.7 > 154.1 (−42)	40.7
244	Triadimefon	5.60	293.1	[M + H]^+^	294.0 > 69.1 (−22)	294.0 > 197.0 (−16)	66.3
245	Triadimenol	5.74	295.1	[M + H]^+^	296.0 > 70.0 (−12)	296.0 > 99.0 (−16)	9.4
246	Tri-allate	8.31	303.0	[M + H]^+^	303.7 > 86.1 (−17)	303.7 > 142.9 (−27)	94.5
247	Triazophos	5.75	313.1	[M + H]^+^	313.5 > 162.0 (−19)	313.5 > 97.0 (−36)	34.0
248	Tribenuron-methyl	4.44	395.1	[M + H]^+^	395.8 > 155.0 (−15)	395.8 > 181.0 (−21)	64.7
249	Tribufos	9.02	314.1	[M + H]^+^	314.9 > 169.0 (−17)	314.9 > 112.9 (−23)	35.9
250	Trichlorfon	3.31	255.9	[M + H]^+^	256.9 > 109.1 (−18)	256.9 > 221.0 (−11)	30.1
251	Tricyclazole	3.56	189.0	[M + H]^+^	189.5 > 163.0 (−22)	189.5 > 136.0 (−27)	98.4
252	Trifloxystrobin	7.34	408.1	[M + H]^+^	408.6 > 186.0 (−19)	408.6 > 145.0 (−43)	97.4
253	Triflumizole	7.40	345.1	[M + H]^+^	346.0 > 277.9 (−11)	346.0 > 43.1 (−28)	70.9
254	Triflumuron	6.86	358.0	[M + H]^+^	358.8 > 156.0 (−18)	358.8 > 139.0 (−30)	76.5
255	Trimethacarb	4.71	193.2	[M + H]^+^	193.7 > 137.1 (−12)	193.7 > 107.0 (−36)	57.5
256	Triticonazole	5.90	317.1	[M + H]^+^	318.1 > 70.0 (−22)	318.1 > 125.0 (−37)	9.5
257-1	Uniconazole_1	5.55	291.1	[M + H]^+^	291.9 > 70.1 (−24)	291.9 > 125.0 (−32)	65.5
257-2	Uniconazole_2	5.78	291.1	[M + H]^+^	291.9 > 70.1 (−24)	291.9 > 125.0 (−32)	72.6
258	Vamidothion	3.21	287.0	[M + H]^+^	287.7 > 145.8 (−18)	287.7 > 118.0 (−23)	92.9
259	XMC	4.34	179.1	[M + H]^+^	180.1 > 123.1 (−12)	180.1 > 108.1 (−27)	35.0
260	Zoxamide	6.73	335.0	[M + H]^+^	335.8 > 186.9 (−22)	335.8 > 159.0 (−40)	75.9

^1^ Collision energy; ^2^ Qualifier/Quantifier.

**Table 4:** Linearity of calibration (r^2^), accuracy and precision in intra-day and inter-day measurements, recovery, and matrix effect results for 260 pesticides.

No.	Compound Name	*r^2^*	Accuracy and Precision (Intra-Day)								Accuracy and Precision (Inter-Day)								Recovery, % (RSD, %)			ME^1^ %
			10 ng/mL		50 ng/mL		150 ng/mL		250 ng/mL		10 ng/mL		50 ng/mL		150 ng/mL		250 ng/mL		10 ng/mL	50 ng/mL	250 ng/mL	
			RE %	RSD %	RE %	RSD %	RE %	RSD %	RE %	RSD %	RE %	RSD %	RE %	RSD %	RE %	RSD %	RE %	RSD %				
1	Acetamiprid	1.000	1.5	5.5	−2.8	2.6	−1.2	5.6	−4.1	1.4	2.0	10.1	1.2	4.0	−1.2	2.9	−3.6	7.0	85.4 (3.8)	91.7 (4.3)	83.9 (3.6)	−44.0
2	Alachlor	0.999	9.7	10.3	3.8	4.8	−4.5	4.8	−8.7	1.2	6.1	18.3	0.9	5.1	−2.7	3.7	−6.0	5.9	80.6 (14.7)	83.6 (12.6)	71.8 (6.6)	1.1
3	Allidochlor	1.000	17.1	11.9	10.3	6.6	1.0	2.4	−2.7	2.8	10.0	14.2	4.0	4.8	−1.3	2.8	−2.2	4.2	82.5 (18.0)	91.6 (5.0)	84.4 (7.8)	−25.5
4	Ametoctradin	0.999	2.1	10.2	−1.3	2.2	−5.5	2.2	−6.8	2.4	2.7	14.9	0.9	4.3	−4.1	5.3	−6.0	5.3	92.6 (13.7)	84.1 (3.5)	76.1 (4.8)	−0.8
5	Ametryn	1.000	4.9	9.1	0.6	3.0	−3.9	2.0	−6.1	2.2	−9.0	16.4	−4.0	2.6	−5.2	1.7	−4.6	6.8	90 (12.3)	93.2 (9.6)	83.9 (3.9)	−7.8
6	Amisulbrom	0.997	−8.6	14.6	−0.9	11.6	−4.3	8.4	−4.4	5.2	2.1	5.5	5.6	8.0	1.2	5.7	−3.4	10.1	110.3 (7.8)	97.3 (18.1)	83.8 (1.6)	−0.6
7	Anilofos	0.999	−0.5	10.6	−0.1	8.0	−7.0	4.1	−9.2	2.9	13.2	9.4	0.8	2.3	−3.9	3.9	−8.4	6.3	73.3 (6.9)	97 (12.6)	78 (4.0)	−3.1
8	Asulam	0.999	−11.9	16.0	10.4	5.6	6.9	4.6	1.6	6.1	5.8	14.1	6.1	4.7	3.0	4.8	−8.1	2.3	84.1 (15.3)	84.2 (4.1)	83.3 (3.4)	−51.1
9	Atrazine	0.999	−1.0	3.2	5.4	2.7	2.0	1.6	−6.2	2.8	−8.2	8.2	4.0	5.3	−3.4	5.1	−8.6	4.3	76.1 (6.5)	89.4 (8.8)	79.1 (4.4)	−16.5
10	Azaconazole	0.997	−10.4	14.2	7.1	3.4	−3.7	4.6	−7.4	0.7	−8.5	16.0	6.5	3.0	−3.0	4.2	−8.6	5.3	94.2 (10.4)	98.5 (13.3)	80.2 (3.9)	−8.3
11	Azamethiphos	1.000	−4.0	8.7	−0.1	2.0	−0.3	2.7	−2.1	3.3	−8.0	7.4	2.5	5.4	−1.7	6.1	−4.1	7.1	86.2 (11.3)	87.5 (10.0)	85.8 (4.7)	−43.7
12	Azimsulfuron	1.000	−1.5	8.9	−0.1	2.8	−3.4	3.3	−9.1	2.1	−2.0	15.8	−0.6	4.9	−6.4	4.6	−5.9	4.8	80.2 (7.4)	91.4 (8.5)	80.3 (4.9)	29.8
13	Azinphos−methyl	0.998	11.1	11.0	−2.4	8.7	−4.0	5.4	−5.7	2.6	−15.0	11.4	−5.5	5.8	0.3	4.4	−7.9	6.8	97.1 (7.9)	94.3 (2.5)	79.2 (1.4)	−7.6
14	Azoxystrobin	1.000	0.9	3.9	−3.6	3.4	−5.2	2.4	−6.5	2.0	−0.8	5.9	−1.6	3.7	−4.3	1.1	−3.7	5.6	102 (5.0)	90.4 (4.8)	83.6 (1.7)	−6.1
15	Bendiocarb	0.998	−8.4	8.4	4.3	6.4	−0.2	1.5	−5.3	1.1	−11.9	12.3	0.0	5.4	0.9	1.9	−5.3	7.8	89.7 (15.9)	98.6 (5.9)	82.6 (5.2)	−26.8
16	Bensulfuron−methyl	1.000	−9.2	8.9	0.3	3.7	−5.9	6.1	−10.3	1.9	−5.4	9.2	−0.2	4.1	−4.1	4.3	−3.7	6.5	101.5 (10.7)	94.9 (13.1)	79.6 (4.3)	36.1
17	Bentazone	0.993	−16.1	14.3	12.7	5.2	5.5	4.7	−2.9	3.9	12.8	9.5	8.3	6.4	2.1	6.1	−7.8	7.3	87.2 (17.2)	88.5 (3.5)	85.1 (4.9)	−12.3
18	Benthiavalicarb−isopropyl	0.998	4.1	11.7	−2.5	5.8	1.9	7.7	−6.5	3.8	−13.0	13.7	2.1	6.4	−2.3	5.7	−6.5	9.1	89.8 (7.5)	91.7 (13.5)	81.5 (6.2)	1.4
19	Benzobicyclon	0.994	1.5	17.2	7.7	9.3	6.4	6.1	−9.4	3.2	−7.5	16.5	14.1	5.4	−1.1	8.7	−6.9	2.4	84.3 (8.8)	85.8 (1.1)	84.6 (4.3)	6.4
20	Benzoximate	0.999	3.4	13.4	−1.7	14.7	−1.8	4.2	−9.9	3.0	6.7	19.5	−0.2	10.1	−2.3	3.9	−5.7	6.1	106.5 (15.2)	96.7 (9.5)	77.4 (8.1)	−2.4
21	Boscalid	0.999	12.5	11.0	−3.1	7.5	1.0	3.9	−8.3	4.0	−13.0	19.9	1.0	7.9	−3.1	4.8	−7.9	6.2	80.9 (18.4)	96.2 (9.1)	77.2 (1.4)	2.6
22	Bromacil	0.997	−5.1	10.8	9.1	5.7	3.5	2.7	−5.1	3.9	−8.1	9.2	7.7	5.8	2.6	3.4	−7.9	4.6	75.5 (4.6)	93.5 (6.6)	79.6 (3.9)	−33.9
23	Bromobutide	0.998	8.1	10.5	10.7	4.9	0.8	3.0	−4.6	1.9	−0.6	16.4	7.0	7.4	0.8	2.4	−2.3	8.1	99.2 (16.1)	91 (16.2)	79 (4.1)	−5.2
24	Bupirimate	0.999	12.4	7.6	−1.8	4.3	−3.2	2.5	−4.7	3.4	−4.3	17.6	−3.2	5.4	−5.0	7.4	−5.0	7.5	91.7 (9.9)	88.6 (7.6)	79.8 (9.7)	−3.0
25	Buprofezin	1.000	11.6	8.9	0.0	7.3	−5.1	2.9	−7.8	2.9	−5.5	7.3	−0.7	6.0	−2.2	4.6	−5.6	10.1	75.6 (6.4)	80.8 (6.8)	75.5 (7.2)	−7.0
26	Butachlor	1.000	−13.1	17.0	−4.8	7.0	−6.1	2.8	−6.7	3.9	−7.2	9.8	1.6	5.8	−2.5	5.1	−2.9	6.2	86.8 (9.4)	89.6 (8.7)	76.4 (4.0)	−1.5
27	Butafenacil	0.997	7.4	9.5	10.3	2.9	1.0	3.7	−8.9	5.7	−3.4	9.2	1.7	9.1	−3.2	5.9	−8.4	10.8	99.9 (12.2)	89.6 (14.4)	72.8 (10.1)	9.0
28	Cadusafos	0.998	−10.6	10.9	1.2	1.9	−3.2	3.7	−7.6	2.9	−12.4	13.6	0.7	7.1	−3.4	3.3	−7.5	7.0	78.3 (6.1)	89.8 (8.1)	76.5 (2.2)	−2.2
29	Carbaryl	0.999	9.5	9.2	6.9	5.1	2.0	1.7	−3.3	2.5	−7.3	14.1	4.3	3.9	−2.1	4.0	−5.5	5.4	87.1 (9.5)	91.8 (9.1)	85.3 (7.2)	−31.0
30	Carbendazim	1.000	−0.4	3.3	1.9	2.8	−0.3	1.8	-4.2	1.1	0.0	9.5	-1.1	2.6	-4.2	3.3	-4.9	5.1	99.9 (12.1)	88.6 (12.7)	79.3 (2.8)	-46.7
31	Carbofuran	0.997	8.3	7.5	4.6	2.9	-0.3	2.0	-5.3	1.2	-2.7	9.3	2.6	3.8	-2.2	2.3	-8.3	6.6	85.9 (8.5)	93 (9.8)	77.4 (2.6)	-21.8
32	Carbophenothion	1.000	3.6	13.6	1.7	5.9	-0.8	2.7	-5.2	2.2	0.3	9.4	-0.9	4.6	-2.7	2.2	-5.1	5.5	95.3 (6.1)	90 (12.5)	77.1 (6.4)	-0.1
33	Carboxin	0.999	-16.4	10.4	2.9	5.7	-0.2	4.2	-7.1	3.1	-1.3	13.3	1.1	4.8	-1.0	1.8	-7.6	6.1	91.3 (12.7)	88.9 (7.1)	80 (2.2)	-24.2
34	Carpropamid	0.999	-5.3	16.0	1.7	10.7	-5.5	1.4	-9.8	4.2	8.3	12.8	0.1	6.6	-4.6	6.5	-8.4	5.1	77.6 (19.5)	91.4 (15.5)	76.7 (4.1)	-4.0
35	Cartap	0.995	-2.2	4.9	4.4	7.0	-4.4	4.3	-14.3	5.5	15.0	10.8	0.1	5.4	-0.9	9.8	0.4	12.2	80.8 (0.8)	85.2 (9.7)	70.4 (5.7)	-5.7
36	Chlorantraniliprole	0.996	3.6	6.9	-2.7	5.8	-3.0	5.2	-7.8	1.5	-1.4	8.4	0.2	8.4	-3.2	4.6	-7.3	8.5	113.8 (4.9)	97.2 (13.9)	83.5 (1.6)	4.4
37	Chlorfenvinphos	0.997	-15.1	18.0	3.5	4.8	-1.2	4.8	-5.1	3.9	4.5	12.7	3.7	4.9	0.2	4.9	-4.6	9.9	80.1 (10.0)	86.8 (2.1)	76.4 (7.6)	0.2
38	Chlorfluazuron	0.991	-3.7	7.4	-2.4	4.5	-6.0	10.2	-3.7	8.6	-6.5	10.2	-4.0	7.7	-5.4	10.5	-5.3	12.3	81.9 (14.9)	81.4 (9.0)	76.1 (4.6)	1.2
39	Chloridazon	0.999	15.1	7.1	2.4	5.1	6.5	4.4	-2.6	4.8	9.7	5.8	4.5	6.3	6.0	4.3	−8.4	7.7	66.7 (17.1)	83.7 (8.2)	89.3 (2.5)	−56.0
40	Chlorotoluron	1.000	6.7	4.8	1.6	1.9	1.1	2.0	−0.7	1.6	10.5	6.9	−0.7	2.4	−2.0	4.0	−5.0	5.0	102 (2.5)	88.8 (5.4)	82 (0.6)	−37.1
41	Chlorsulfuron	0.996	11.7	12.0	10.4	4.7	0.3	2.3	−7.8	2.7	−9.8	9.8	6.0	2.8	−6.7	3.2	−6.5	7.1	55.2 (12.7)	95.3 (8.3)	81.1 (2.0)	39.8
42	Chromafenozide	0.999	−18.4	9.9	4.6	8.6	−6.7	2.7	−8.2	4.7	−1.6	12.0	6.0	6.6	−3.9	5.2	−7.7	4.4	89.9 (10.4)	88.5 (15.9)	80.7 (3.6)	−7.5
43	Cinmethylin	0.999	−5.2	13.4	3.2	9.3	−5.5	5.1	−11.9	3.6	1.8	16.6	3.0	14.7	−4.5	3.2	−6.3	6.6	101.1 (2.6)	86.1 (2.9)	72.7 (9.7)	−0.9
44	Clofentezine	0.996	−13.7	11.4	4.3	6.5	−1.9	3.0	−10.0	1.4	−0.4	9.4	8.6	4.0	−3.7	4.6	−6.7	8.9	94.4 (8.8)	79.1 (5.8)	71 (4.9)	4.3
45	Clomazone	1.000	−5.6	8.3	0.7	3.3	−4.9	3.1	−8.9	3.2	−13.7	13.8	0.1	1.9	−2.7	3.1	−6.1	5.3	97.2 (8.5)	92.9 (1.8)	80.5 (3.9)	−9.0
46	Clothianidin	0.999	−1.8	10.2	−4.4	9.6	−3.9	6.6	−5.7	4.7	10.7	8.3	−6.1	6.9	−3.3	10.0	−7.3	6.2	82 (10.4)	75.8 (14.6)	70.8 (5.9)	−44.0
47	Cyanazine	0.999	−6.3	5.1	0.1	6.8	−2.6	2.3	−4.9	1.3	−2.9	7.5	0.0	4.6	−4.8	2.2	−5.9	3.7	100 (0.5)	92.9 (2.5)	79.8 (5.3)	−23.4
48	Cyazofamid	0.992	−16.7	16.9	9.8	6.3	−0.5	5.7	−10.9	1.7	−0.1	9.2	10.5	5.3	−3.7	3.0	−11.3	2.6	83.7 (2.2)	100.9 (1.8)	76.2 (6.1)	3.5
49	Cyclosulfamuron	0.999	9.9	6.0	2.7	3.9	−8.0	4.5	−12.2	1.7	−8.3	17.8	−2.0	1.1	−3.9	4.2	−7.3	4.5	96.1 (12.8)	94.7 (11.0)	84 (4.0)	12.1
50	Cymoxanil	1.000	6.7	8.6	9.8	8.4	7.8	3.7	−0.1	2.1	9.4	19.1	6.9	4.8	3.8	5.1	−4.8	7.9	80.5 (13.9)	91.5 (0.9)	86.9 (6.5)	−61.7
51	Cyproconazole	0.998	−5.2	12.1	3.4	6.7	−4.8	5.3	−9.8	4.1	−9.5	6.8	6.0	3.3	−3.8	4.3	−7.5	6.4	105.3 (9.7)	93 (8.6)	76 (2.4)	2.8
52	Cyprodinil	0.999	7.8	12.5	3.8	7.9	−3.7	4.6	−3.9	3.1	−1.5	15.6	2.8	2.7	−4.2	3.4	−3.0	7.0	87.2 (18.8)	96.9 (13.8)	85 (4.8)	−14.3
53	Daimuron	0.997	1.5	6.6	−0.1	5.8	−1.6	3.7	−6.4	0.9	−1.2	7.3	3.1	4.8	−3.5	2.4	−6.3	7.0	87.1 (3.0)	87.8 (8.1)	79.3 (6.2)	−3.2
54	Diazinon	1.000	−2.9	5.0	2.2	2.8	−3.3	6.1	−4.7	5.5	3.4	13.6	0.9	4.2	−4.7	4.1	0.0	7.3	81.4 (6.4)	86.3 (3.0)	79.9 (7.6)	−7.2
55	Dicrotophos	1.000	0.7	8.0	2.0	2.5	−4.3	1.4	−7.2	2.6	4.0	13.1	4.5	4.4	−1.2	4.2	−7.4	6.0	93.8 (4.2)	90.8 (6.9)	85.2 (6.8)	−41.5
56	Diethofencarb	0.999	−0.6	4.6	−5.4	6.9	−1.3	2.0	−7.3	2.7	−6.6	11.8	2.0	7.9	−2.1	1.5	−4.5	5.1	86.2 (15.9)	87.4 (6.2)	76.6 (4.5)	−3.4
57	Difenoconazole	1.000	1.3	6.8	−0.8	4.9	−4.1	3.6	−5.3	1.2	−2.2	17.9	−4.1	3.4	−7.5	3.5	−4.2	4.7	94.1 (4.6)	90.5 (8.4)	80.4 (1.9)	2.6
58	Diflubenzuron	0.998	−3.3	19.7	9.2	14.9	−1.6	4.6	−8.5	3.6	0.4	9.9	0.1	14.0	3.9	2.2	−9.4	9.7	93.5 (5.5)	88.6 (12.0)	73 (6.8)	4.9
59	Diflufenican	0.999	5.7	4.5	−1.0	4.9	−8.4	2.8	−6.7	1.9	3.4	15.5	−3.5	9.1	−4.0	4.9	−3.4	8.5	97.8 (4.6)	88.4 (7.3)	73.7 (11.6)	−0.6
60	Dimethachlor	0.997	−1.6	15.5	5.0	3.6	0.2	2.5	−6.4	2.0	−5.4	11.6	3.4	3.3	−2.9	2.4	−6.3	4.9	96.6 (10.7)	93.8 (5.4)	80.9 (2.8)	−8.7
61	Dimethametryn	1.000	9.8	5.8	−1.1	4.4	−2.1	2.9	−4.1	1.8	4.1	6.5	−4.6	7.6	−4.2	3.3	−1.8	6.8	89.5 (4.7)	92.3 (10.0)	81.3 (2.6)	−5.8
62	Dimethenamid	0.999	19.5	2.7	1.6	4.7	−4.3	1.5	−8.1	3.0	2.0	13.1	2.0	4.1	−0.3	2.4	−7.0	5.7	77.4 (8.3)	85.5 (5.3)	79.6 (6.2)	−10.1
63	Dimethoate	0.998	−1.2	10.0	2.0	3.4	0.5	4.6	−2.0	2.0	−0.1	10.9	2.6	4.5	0.4	2.0	−3.4	7.4	96.6 (0.7)	93.5 (4.7)	86.5 (3.9)	−44.2
64	Dimethomorph	0.999	13.3	7.7	0.3	7.8	−4.2	2.2	−8.8	0.9	1.6	15.6	1.2	5.3	−1.4	3.9	−5.8	7.4	79.2 (6.5)	86.3 (9.3)	76.6 (3.6)	29.9
65	Diniconazole	0.999	8.7	15.6	−0.3	6.8	−4.9	7.0	−9.4	2.6	−4.2	13.5	−6.8	5.3	−3.2	6.2	−7.9	7.2	108.4 (3.2)	82.5 (8.6)	76.3 (3.5)	−2.6
66	Diphenamid	0.999	−5.4	4.0	1.5	2.0	−2.1	2.3	−5.2	1.8	−2.0	4.6	1.5	3.3	−3.7	2.0	−6.3	4.3	88.7 (6.8)	91.7 (7.7)	81.3 (1.1)	−9.2
67	Diuron	0.996	−7.8	5.6	11.8	4.4	3.2	3.2	−7.5	1.6	−1.5	5.8	7.3	6.3	0.4	3.3	−7.6	5.7	77.9 (6.3)	95 (7.9)	81.8 (4.0)	−18.5
68	Edifenphos	0.998	−4.5	11.4	−0.2	2.2	−1.1	3.9	−5.9	3.2	−11.9	16.1	0.7	6.2	−6.5	2.5	−6.7	4.4	99.5 (10.0)	83.7 (10.5)	75.4 (8.6)	−0.7
69	Emamectin B1a	1.000	14.7	3.5	−3.7	3.6	−7.3	2.2	−6.0	2.0	5.6	10.4	−4.6	1.6	−6.8	3.1	−4.2	5.4	96.2 (2.4)	89.4 (7.2)	83.3 (3.3)	−0.6
70	Emamectin B1b	1.000	9.2	7.4	−1.3	11.0	−14.0	3.9	−12.2	2.8	9.4	9.6	−5.7	13.6	−6.3	9.5	−7.5	5.6	98.5 (13.3)	87.4 (19.4)	83.6 (7.9)	0.1
71	EPN	0.997	−8.6	4.2	7.5	2.6	0.8	2.8	−7.5	1.9	−11.4	9.4	6.9	0.6	−1.8	1.8	−8.3	3.4	96.6 (7.5)	95.2 (6.7)	78.5 (3.3)	−8.9
72	Epoxiconazole	0.999	4.7	9.3	2.8	5.1	−4.8	2.9	−6.0	2.3	−3.8	13.4	−0.5	8.0	−1.4	3.3	−6.8	9.6	92.4 (10.4)	87 (12.6)	77.9 (8.6)	2.9
73	Ethaboxam (EBX)	0.999	−8.2	5.5	2.3	3.1	−2.7	2.6	−7.0	1.6	−8.9	4.9	2.4	7.6	−1.9	4.2	−6.4	6.2	91.8 (6.7)	94.7 (3.2)	79 (5.5)	9.6
74	Ethametsulfuron−methyl	1.000	10.2	4.5	1.8	3.5	−2.8	3.8	−7.8	2.7	0.3	10.9	1.1	3.8	−4.9	3.4	−6.5	4.2	100.3 (5.2)	93.3 (8.9)	80.5 (4.8)	33.7
75	Ethiofencarb	0.999	−7.8	3.4	−1.0	3.9	−2.6	1.4	−4.5	3.0	1.2	9.2	1.7	3.0	−2.4	2.3	−5.1	3.6	89.9 (12.9)	92.7 (4.2)	80.9 (5.9)	−21.6
76	Ethion	0.999	−7.6	8.0	1.5	5.1	−3.3	1.8	−6.6	3.1	−4.8	7.2	0.7	4.8	−2.9	4.0	−5.8	7.4	94.8 (13.9)	81.8 (5.4)	75.5 (9.7)	−3.4
77	Ethoprophos	0.998	−8.7	7.5	−1.7	5.5	−4.9	2.9	−10.5	2.5	−4.8	15.1	5.3	7.9	1.0	4.6	−5.4	9.5	71.4 (7.4)	89.8 (6.0)	77.4 (6.2)	−5.7
78	Ethoxyquin	0.999	−5.0	7.1	−7.3	4.6	−9.8	8.0	−4.7	6.6	−18.2	13.2	−5.0	7.4	−10.6	4.2	0.1	7.1	105.1 (11.5)	85.7 (5.0)	79.7 (10.4)	−8.4
79	Ethoxysulfuron	0.998	7.1	12.3	6.3	6.4	−4.2	2.4	−14.4	2.1	0.9	10.9	6.9	5.0	−7.9	5.1	−8.0	8.4	90.1 (15.0)	98.1 (15.4)	78.9 (6.8)	38.5
80	Etoxazole	1.000	13.7	4.1	−0.5	6.6	−9.4	3.6	−9.0	5.8	6.3	6.2	−5.3	3.0	−3.2	5.7	−3.2	5.4	92.9 (5.3)	87.1 (11.5)	79.9 (4.7)	−3.0
81	Etrimfos	1.000	−3.5	17.9	−0.4	4.5	−2.6	3.3	−4.0	2.1	4.6	7.1	−1.5	2.1	−5.2	3.7	−1.1	10.8	100.3 (4.0)	95.1 (6.5)	83.3 (4.8)	−3.8
82	Fenamidone	1.000	14.3	9.3	−3.2	3.7	−4.2	2.4	−8.1	2.8	2.3	10.5	−0.1	5.4	−2.8	7.2	−3.4	6.2	100.4 (10.6)	91.1 (5.9)	80.5 (0.6)	4.3
83	Fenamiphos	1.000	15.3	7.0	1.1	0.8	−2.8	1.9	−5.4	1.5	7.3	8.1	−0.2	3.4	−5.2	5.5	−6.8	8.0	96.2 (2.1)	84.8 (10.2)	75.3 (6.4)	10.9
84	Fenazaquin	1.000	2.8	2.1	−1.9	5.1	−4.5	2.5	−6.3	0.8	1.8	4.2	−1.9	5.9	−2.5	5.2	−2.6	8.1	82.6 (7.9)	79.6 (9.5)	71.5 (4.1)	1.3
85	Fenbuconazole	0.999	6.6	13.1	−2.5	7.1	−3.1	4.8	−7.1	2.0	−8.4	16.6	1.8	5.5	−3.1	7.2	−4.4	4.9	73.5 (16.4)	86.6 (7.5)	74.4 (3.3)	5.0
86	Fenhexamid	0.998	−4.3	19.3	5.4	7.7	−0.2	5.9	−9.7	5.7	−3.2	6.6	0.7	6.6	−4.9	8.3	−9.3	6.7	78.1 (6.8)	90.9 (8.4)	77.3 (1.7)	−0.6
87	Fenobucarb (BPMC)	1.000	3.1	4.9	1.2	2.6	−4.0	3.4	−5.7	1.5	6.0	7.6	1.5	5.2	−3.7	5.3	−4.9	6.0	82.7 (11.5)	86.3 (8.5)	80.9 (4.3)	−10.2
88	Fenothiocarb	0.999	−4.9	8.9	2.8	4.2	−4.3	2.8	−10.9	1.0	−9.3	13.3	1.9	7.2	−2.9	4.6	−6.3	8.9	71.8 (13.0)	87.1 (6.4)	72.7 (4.4)	0.1
89	Fenoxanil	0.998	−10.3	10.4	2.9	5.7	−2.2	1.8	−7.7	2.4	−2.1	16.9	3.3	3.5	−0.7	3.6	−6.0	6.2	73.5 (18.2)	87.4 (8.8)	72 (9.6)	0.7
90	Fenoxycarb	0.998	−6.8	12.8	2.7	5.4	−5.3	2.6	−7.9	2.5	−3.6	7.5	0.3	5.6	−1.1	6.3	−4.6	3.7	90.7 (4.2)	83.4 (5.6)	72.2 (3.6)	−2.1
91	Fenthion	0.999	19.3	5.7	6.9	14.5	−0.1	3.4	−5.5	4.2	−2.6	16.1	1.9	7.7	−3.5	4.8	−6.6	5.4	96.7 (6.6)	92.5 (7.5)	79.6 (7.8)	−5.4
92	Ferimzone	1.000	−4.5	8.6	−0.8	5.2	−5.3	3.0	−6.5	1.3	−3.9	3.6	−4.8	5.0	−3.6	4.4	−6.0	3.5	113.6 (6.1)	86.2 (8.2)	74.9 (5.9)	−2.2
93	Fipronil	0.999	14.0	7.4	−1.1	6.4	−10.4	7.7	−11.0	1.7	14.8	13.2	−4.2	8.1	−8.8	7.9	−7.0	5.2	100.8 (6.0)	100.8 (16.4)	83.5 (4.0)	12.5
94	Fluacrypyrim	0.999	−8.4	6.7	3.5	6.3	−3.8	4.0	−5.0	3.3	0.5	15.7	4.5	1.9	−4.9	3.7	−8.4	4.1	95.2 (5.7)	95.2 (6.9)	77.5 (5.5)	−0.7
95	Fluazinam	1.000	−0.7	10.7	3.0	6.6	−1.7	3.4	−0.6	3.0	10.9	6.1	−2.3	5.6	−5.2	5.3	−3.7	5.5	113.9 (8.7)	94.2 (11.2)	84.4 (7.7)	11.0
96	Flucetosulfuron	0.999	9.6	9.2	1.2	2.7	−8.4	3.8	−11.3	2.1	1.1	13.6	−0.8	5.6	−8.3	3.9	−6.5	7.2	90.5 (7.6)	87.8 (8.4)	77.9 (4.0)	56.2
97	Flufenacet	0.996	−15.7	15.6	7.2	8.1	−3.8	3.5	−14.5	3.7	1.5	7.6	7.7	3.7	−4.7	2.7	−10.7	2.9	79 (2.5)	91.6 (7.8)	74.1 (3.9)	−4.7
98	Flufenoxuron	1.000	8.8	5.2	−1.8	4.4	−6.2	2.5	−4.7	1.4	4.7	7.8	−3.6	3.4	−4.9	4.3	−4.3	7.6	95.1 (8.2)	86.9 (6.0)	77.9 (5.8)	−2.6
99	Fluopicolide	0.992	−9.0	17.5	8.6	5.3	−1.7	1.9	−14.7	3.8	4.0	9.7	9.2	6.1	0.8	5.2	−9.2	8.0	98.1 (9.2)	95.8 (4.7)	79.7 (10.6)	−3.9
100	Fluopyram	0.998	4.5	9.5	−2.2	4.9	−6.6	5.0	−10.3	1.6	−8.1	14.5	2.7	5.5	−1.6	7.0	−10.2	4.3	82.9 (12.9)	89.8 (5.4)	72.1 (6.2)	−0.6
101	Flusilazole	1.000	−5.6	6.1	−2.1	8.5	−4.1	2.5	−8.6	3.6	1.2	9.2	−0.8	9.5	−3.6	5.5	−4.9	6.3	89.2 (18.2)	78.3 (5.3)	73.6 (3.4)	−1.5
102	Flusulfamide	0.999	10.0	11.6	1.8	5.5	−3.2	4.4	−9.6	3.5	5.7	7.2	−0.7	8.3	−4.5	6.9	−4.8	8.2	83.3 (2.4)	86.9 (8.6)	83 (1.6)	2.8
103	Flutolanil	0.995	−1.4	17.3	8.7	5.2	−1.8	3.2	−10.4	3.0	−4.6	7.3	1.8	6.0	−6.0	4.2	−10.0	6.3	104.2 (5.5)	91.4 (8.7)	77.8 (2.1)	−7.2
104	Fluxapyroxad	0.988	1.7	6.8	11.3	6.8	−1.0	8.2	−9.8	6.3	−10.1	17.3	14.0	8.4	1.3	5.5	−4.5	3.7	54.2 (11.9)	90.3 (11.4)	80 (4.4)	−2.4
105	Fonofos	1.000	9.3	19.6	−6.1	10.9	−6.6	5.7	−7.5	3.9	−0.8	18.3	−2.9	12.3	1.3	6.7	−3.0	9.5	86.4 (3.7)	82.2 (14.5)	77.9 (4.4)	8.7
106	Forchlorfenuron	1.000	−6.6	10.6	0.1	4.2	−5.9	2.1	−4.8	2.3	−0.7	14.3	2.0	2.0	−3.7	2.7	−1.5	7.8	100.8 (7.3)	93.1 (8.1)	84.9 (5.6)	2.3
107	Fosthiazate	1.000	1.9	2.7	−0.1	2.1	−3.3	2.6	−5.7	2.4	−2.5	6.1	−1.0	4.8	−3.5	2.2	−4.1	6.3	91.6 (4.4)	88.9 (7.2)	83.9 (2.0)	−15.4
108	Furathiocarb	1.000	−7.6	2.5	−4.2	3.4	−4.6	3.7	−9.6	1.5	−5.7	7.6	−1.0	9.7	−2.3	8.4	−3.6	9.9	92.2 (9.8)	81.4 (6.8)	75.9 (5.8)	−4.4
109	Halosulfuron−methyl	0.999	1.9	12.6	−1.7	10.0	−6.4	2.0	−9.2	1.4	−4.0	14.0	−0.1	5.2	−3.2	3.8	−6.3	9.2	70.9 (5.5)	83.1 (8.7)	79.8 (7.7)	13.8
110	Haloxyfop−R−Methyl	0.996	4.0	11.5	4.4	5.3	−4.7	3.2	−11.1	3.5	0.5	7.3	4.0	5.1	−1.2	3.7	−7.1	5.5	72.4 (6.7)	92 (9.7)	77.3 (5.4)	−2.3
111	Hexaconazole	1.000	2.5	10.3	1.0	5.7	−6.4	3.4	−7.9	3.0	−4.3	5.8	−2.2	6.2	−6.4	6.3	−5.1	7.9	79.1 (16.6)	84.4 (1.6)	75.9 (3.8)	−2.7
112	Hexaflumuron	0.999	−6.1	13.0	1.9	11.0	−4.6	2.8	−9.0	3.2	−0.1	17.8	−4.5	10.1	−3.0	3.3	−6.5	4.5	104.1 (2.4)	95.9 (10.4)	93.8 (4.1)	9.4
113	Hexazinone	1.000	2.0	3.0	0.9	2.8	−2.4	2.6	−6.3	0.9	3.8	3.2	0.7	3.3	−2.5	2.5	−5.4	3.8	87.1 (2.3)	90.1 (8.0)	84.6 (2.6)	−23.8
114	Imazalil	0.999	16.8	10.3	0.4	4.0	−5.8	2.0	−6.2	1.5	4.0	17.9	0.4	5.5	−3.8	7.1	−4.8	2.9	96.1 (7.4)	92.7 (8.6)	89.3 (6.0)	−22.2
115	Imazapic	1.000	−3.7	6.8	−1.9	4.9	−3.0	2.0	−3.6	4.2	7.6	18.4	−1.6	3.8	−3.3	5.7	−2.8	2.2	91.8 (11.2)	83 (4.9)	76 (2.5)	−39.6
116	Imazaquin	1.000	6.3	8.7	−1.8	3.7	−4.6	3.4	−9.8	1.6	10.1	6.9	−3.6	5.6	−1.6	5.4	−7.2	4.2	94.7 (14.4)	97.4 (2.8)	84.1 (4.7)	3.3
117	Imazethapyr	0.999	−4.9	19.1	1.7	9.3	−1.0	3.7	−1.6	1.5	−1.8	15.1	−1.5	5.9	0.6	3.1	−5.3	5.7	109.8 (14.3)	90.5 (7.4)	80.3 (4.8)	−29.3
118	Imibenconazole	0.999	16.5	3.6	1.0	6.6	−5.5	3.9	−7.1	2.0	−2.5	13.8	−2.5	10.8	−4.4	2.7	−3.8	5.5	89.3 (13.0)	78.5 (4.2)	72.4 (6.3)	−1.3
119	Imicyafos	0.996	−2.1	12.1	5.9	3.8	8.1	10.7	2.9	4.8	4.6	17.5	5.0	11.3	3.4	14.0	−6.6	8.7	92.2 (10.3)	88.4 (10.6)	87.8 (9.9)	−46.7
120	Imidacloprid	1.000	−4.1	11.8	6.9	5.5	6.1	3.1	1.8	4.7	7.0	12.5	5.8	5.7	4.8	3.1	−4.2	6.4	71.5 (8.2)	79.4 (5.3)	76.7 (1.7)	−28.8
121	Indoxacarb	0.996	−8.2	12.9	−3.2	5.0	−3.5	6.0	−8.6	4.4	−10.1	17.8	2.1	7.8	−5.9	9.5	−4.8	8.4	101 (5.2)	86.2 (16.3)	80.9 (1.2)	4.9
122	Iprobenfos	0.999	6.3	12.7	0.0	4.5	−4.3	2.7	−3.9	2.1	7.0	14.5	2.1	3.4	−2.7	3.8	−4.1	5.2	87.9 (10.6)	82.7 (3.9)	74.6 (4.7)	−9.8
123	Iprovalicarb	0.999	−2.8	9.8	−2.2	3.2	−5.4	1.2	−8.7	2.4	−6.9	7.4	0.0	7.4	−2.3	3.9	−4.1	4.2	90.1 (1.0)	91.7 (8.4)	78.3 (2.0)	−3.4
124	Isazofos	0.997	−8.2	5.6	5.7	2.6	−2.2	3.1	−8.9	1.9	−11.1	2.7	6.0	5.8	−2.5	2.7	−6.9	5.7	78.8 (6.4)	88.8 (8.6)	77.1 (4.1)	−4.0
125	Isoprocarb	1.000	−1.0	4.6	0.0	1.8	−2.0	2.4	−4.7	1.9	3.5	7.3	−0.1	2.9	−3.4	2.5	−4.0	3.5	91.1 (2.5)	92.4 (2.1)	83 (1.2)	−23.9
126	Isoprothiolane	0.998	−7.3	7.3	6.7	1.7	−2.4	2.9	−7.9	2.8	−7.0	7.2	4.3	3.7	−2.8	3.7	−8.2	3.8	75 (6.1)	92 (12.7)	80.4 (2.1)	−8.3
127	Isoproturon	0.999	−4.3	7.7	−1.0	2.8	−3.8	3.8	−6.8	2.0	1.1	4.5	−1.1	4.2	−4.9	2.9	−4.8	5.9	89 (9.6)	94.6 (7.4)	82.4 (4.5)	−13.8
128	Isopyrazam	0.999	−7.1	10.7	−1.0	4.1	−4.6	2.1	−11.9	3.3	−8.9	17.3	2.5	10.7	−0.8	6.2	−7.3	7.8	93.3 (18.2)	87 (11.8)	75.7 (3.7)	−0.7
129	Isoxathion	0.999	−6.5	13.1	2.7	5.4	−0.7	2.7	−5.9	2.7	0.8	9.8	0.0	4.8	−3.2	4.4	−6.0	8.2	80.8 (8.1)	87.2 (9.4)	74.2 (5.1)	−1.4
130	Kresoxim−methyl	0.996	7.2	12.9	4.0	7.9	0.8	5.1	−5.8	3.6	−12.8	15.0	10.3	10.0	3.3	6.1	−5.3	4.3	105.2 (11.6)	79.7 (9.6)	75.1 (4.3)	−11.9
131	Linuron	0.999	−15.7	18.0	−0.3	2.1	−2.5	2.3	−7.7	1.1	−8.3	15.8	−0.2	6.0	−5.6	4.2	−7.3	8.7	79.6 (6.9)	91.5 (15.0)	82.9 (4.8)	−10.2
132	Mandipropamid	0.999	−1.9	6.0	0.8	2.9	−2.2	1.7	−7.2	1.3	−7.0	10.2	4.6	4.4	−4.5	2.1	−5.3	6.6	86 (9.5)	92.8 (1.6)	78.9 (3.9)	7.8
133	Mecarbam	0.998	4.8	5.4	6.5	4.0	−0.9	2.8	−6.9	2.7	−5.6	17.3	4.7	4.8	0.9	2.9	−6.9	6.8	78.1 (7.5)	93.8 (13.5)	79.2 (5.9)	−5.0
134	Mefenacet	0.998	−1.9	5.8	5.0	2.7	−0.9	2.0	−4.6	1.5	−3.1	7.4	2.4	6.1	−0.5	4.6	−6.5	4.2	95.8 (11.3)	91.5 (7.6)	81 (2.2)	−6.9
135	Mefenpyr−diethyl	0.999	3.7	5.7	0.0	4.1	−3.9	4.1	−7.6	2.8	−1.6	7.1	2.1	4.6	−3.6	4.9	−3.6	5.6	72.6 (9.5)	87.8 (13.7)	75.1 (3.7)	0.6
136	Mepronil	0.996	12.2	10.0	9.2	1.7	0.6	2.5	−8.8	2.2	−16.6	15.2	5.4	3.0	−0.5	3.1	−8.4	3.7	78.3 (17.9)	94.4 (5.1)	77.4 (4.4)	−4.7
137	Metalaxyl	1.000	2.0	7.0	0.9	2.1	−4.7	2.0	−5.2	1.7	−4.7	12.9	−2.7	5.0	−4.6	2.1	−5.6	4.5	100 (7.2)	89.2 (8.7)	80.1 (3.9)	−6.8
138	Metamifop	0.998	10.7	3.4	−4.2	9.6	−4.5	1.6	−6.8	3.1	5.4	12.8	1.1	5.0	−4.0	3.1	−2.7	9.9	86.8 (6.5)	86.4 (5.0)	76 (7.0)	−6.1
139	Metazosulfuron	0.999	−17.4	7.7	−4.6	3.7	−6.3	3.4	−11.1	2.8	−13.2	11.8	2.6	7.6	−5.2	5.1	−6.9	8.1	89.6 (12.1)	81 (8.8)	76.3 (5.3)	65.1
140	Metconazole	1.000	−4.5	10.8	−4.1	3.1	−3.9	2.8	−8.8	1.4	3.9	10.4	−1.1	5.4	−5.0	4.9	−5.0	6.2	89 (1.2)	83.2 (10.9)	76.9 (1.9)	−2.3
141	Methabenzthiazuron	1.000	−0.1	4.9	0.6	2.0	−4.4	1.5	−6.5	1.6	−1.1	6.2	4.1	3.6	−3.0	1.9	−4.9	6.1	88.6 (7.3)	87.6 (6.6)	79 (3.1)	−21.9
142	Methidathion	0.997	3.9	16.3	7.7	7.2	−4.8	2.9	−10.9	2.2	3.3	16.2	5.8	5.4	−3.5	5.4	−8.2	11.5	98 (7.3)	101.4 (8.7)	84 (4.6)	−7.4
143	Methiocarb	0.999	0.1	9.1	3.2	6.4	−1.9	1.2	−6.3	3.3	−1.8	9.8	4.0	6.6	−1.2	2.6	−6.1	4.5	93.3 (3.9)	91.1 (6.7)	77.4 (3.5)	−8.9
144	Methomyl	0.997	8.5	6.7	11.1	6.8	7.0	3.6	−5.5	4.6	4.1	19.1	8.7	8.4	7.5	4.3	−9.8	4.2	103.2 (3.5)	92.3 (10.4)	86.6 (5.7)	−66.3
145	Methoxyfenozide	0.997	10.2	4.8	−2.3	7.6	−8.7	5.3	−13.1	4.1	3.3	19.6	2.3	6.9	−1.1	6.6	−7.2	6.8	71.9 (5.8)	106.1 (6.1)	85.3 (6.4)	−6.8
146	Metobromuron	0.998	8.0	14.0	6.9	4.8	6.4	4.7	2.7	6.8	2.9	19.7	−1.6	5.5	−0.2	5.9	−4.1	7.2	102.7 (19.4)	90.4 (1.4)	86.8 (5.8)	−31.8
147	Metolachlor	0.999	−3.8	7.4	−2.4	7.9	−3.6	3.2	−4.8	3.3	−6.9	12.7	−1.8	6.6	−5.3	5.4	−2.9	5.1	76.8 (9.5)	86 (7.3)	77.5 (2.0)	−3.2
148	Metolcarb	1.000	−10.1	10.2	0.6	2.1	−0.6	3.7	−3.2	1.1	−4.1	14.0	3.7	4.0	−0.6	3.2	−4.7	5.9	97.7 (10.3)	87.7 (4.4)	84.4 (5.4)	−36.3
149	Metominostrobin	1.000	−0.2	3.9	−1.7	3.0	−5.5	4.2	−7.6	1.4	−4.0	12.9	−1.6	3.2	−2.8	1.9	−4.5	4.0	87.6 (4.1)	90.6 (3.5)	81.5 (5.2)	−11.9
150	Metrafenone	1.000	−5.8	12.3	−1.7	4.7	−2.9	1.6	−6.2	0.6	−1.5	14.2	−1.3	7.1	−2.6	3.6	−5.8	8.9	77.8 (9.3)	88.2 (11.0)	76.8 (5.6)	−0.7
151	Mevinphos	1.000	−3.8	6.4	8.4	2.2	3.2	2.3	1.4	1.3	3.7	11.0	1.3	7.2	−0.8	3.0	−3.6	5.0	80.6 (1.5)	90.7 (1.8)	83.6 (4.0)	−47.6
152	Milbemectin A4	0.996	−1.0	9.1	−2.2	4.9	−6.6	5.4	−5.9	3.7	14.6	17.2	−5.5	4.3	−1.7	5.8	1.1	7.9	107.4 (9.9)	79.1 (7.8)	68.5 (5.7)	−8.9
153	Molinate	1.000	17.1	3.5	−2.5	3.3	−8.9	2.8	−8.9	2.8	9.3	12.0	−0.8	4.5	−2.3	3.0	−5.6	5.9	91.9 (5.6)	90.6 (9.8)	79.1 (0.3)	4.2
154	Monocrotophos	0.999	−1.7	8.9	5.7	7.8	3.3	5.4	−5.2	4.4	−4.7	13.1	7.1	5.9	4.2	2.2	−7.5	3.8	90.6 (16.6)	89.3 (5.1)	86 (5.0)	−60.1
155	Myclobutanil	0.996	4.1	5.0	13.1	9.1	−0.4	2.2	−7.8	3.7	−12.5	15.2	14.9	7.0	−0.7	2.5	−6.8	9.2	103.1 (15.5)	94.7 (7.9)	73.9 (5.5)	15.3
156	Napropamide	0.999	1.8	9.6	−1.8	5.1	−8.3	5.1	−10.7	2.7	−4.3	9.9	−1.2	5.4	−2.7	5.1	−8.6	7.3	88.6 (2.2)	86.4 (20.0)	78.9 (1.6)	−4.9
157	Nicosulfuron	1.000	1.3	6.1	−1.3	1.9	−2.5	1.9	−3.6	2.2	4.9	4.2	−2.5	1.5	−4.7	3.2	−5.0	7.0	97.9 (3.5)	89.7 (1.1)	81.4 (2.4)	−1.0
158	Nitenpyram	0.992	−8.3	6.6	10.7	8.1	1.1	5.4	−7.0	7.5	5.7	19.9	6.1	5.9	2.0	3.6	−8.4	5.8	85.5 (8.3)	88 (8.3)	76.1 (11.9)	−15.3
159	Nuarimol	0.999	11.8	18.5	7.1	5.9	−4.6	4.9	−4.6	2.8	−0.7	15.7	1.1	4.6	−4.9	10.0	−3.4	6.4	95.6 (12.9)	83.6 (12.9)	75.8 (4.1)	13.0
160	Ofurace	0.999	1.2	8.3	4.8	3.7	−0.4	3.1	−4.5	3.5	0.8	8.9	−1.2	5.1	−1.4	4.6	−5.6	5.6	92.6 (6.5)	86.7 (12.2)	80.5 (6.3)	−27.5
161	Omethoate	0.999	16.8	17.8	12.8	3.7	8.9	7.4	−5.4	2.2	−2.1	17.2	3.0	9.5	−0.1	10.2	−2.1	12.6	93.5 (5.4)	83.7 (0.6)	99.1 (6.6)	−39.7
162	Orysastrobin	1.000	4.4	4.2	−0.1	1.5	−4.8	3.6	−6.5	2.1	−2.9	7.3	−4.7	5.6	−4.6	3.4	−5.6	3.9	106.1 (5.0)	89.6 (10.3)	82.4 (3.1)	−0.4
163	Oxadiazon	0.998	0.2	15.6	11.8	6.0	−1.5	7.1	−8.9	2.3	12.8	19.9	9.4	6.9	−4.8	5.7	−1.9	7.6	97.5 (16.4)	93.1 (10.0)	85.4 (6.8)	−4.3
164	Oxadixyl	1.000	−9.9	11.7	2.1	4.5	1.4	3.0	−1.8	3.4	−3.4	7.1	4.0	2.9	0.7	3.8	−5.6	3.1	100 (4.6)	90.3 (3.2)	87.5 (2.1)	−51.6
165	Oxamyl	0.998	5.7	7.3	7.5	6.7	5.9	2.6	−2.1	1.8	5.6	12.0	5.9	4.4	2.1	4.4	−5.7	4.9	92.4 (2.6)	96 (5.4)	86.9 (2.5)	−49.5
166	Oxydemeton−methyl	0.998	−6.4	6.0	3.6	2.8	1.9	3.2	−6.3	1.8	0.1	7.1	1.8	4.0	1.9	2.9	−6.0	5.6	102.8 (2.7)	95.5 (5.3)	85.7 (1.0)	−48.8
167	Paclobutrazol	0.996	-6.4	11.7	2.0	6.9	0.5	1.5	-6.7	1.9	-4.4	13.2	4.4	4.4	-2.5	2.3	-7.8	5.0	98 (17.7)	91.7 (9.4)	75.8 (5.5)	6.1
168	Pebulate	0.999	-1.0	19.7	-2.3	11.0	-8.0	3.2	-10.9	7.6	13.6	15.7	-7.3	4.5	0.4	7.5	-6.6	7.7	96.9 (4.2)	90.2 (12.8)	81.7 (5.8)	-8.7
169	Penconazole	0.999	-12.9	7.6	0.3	5.8	-3.7	5.3	-9.4	1.6	6.7	4.1	2.5	6.4	0.7	1.4	-5.3	9.0	86.1 (10.8)	78.2 (8.2)	72.3 (5.2)	-1.6
170	Pendimethalin	1.000	2.3	15.6	-5.2	2.6	-4.2	2.9	-5.7	1.7	10.0	13.9	-4.9	2.8	-2.8	2.2	-1.8	8.3	86.6 (7.1)	80.9 (10.1)	75.2 (6.4)	-2.8
171	Penoxsulam	1.000	1.9	5.9	3.8	2.6	-1.3	2.5	-5.3	1.1	-3.7	12.0	-0.8	5.2	-2.1	4.6	-5.9	8.6	92.5 (6.5)	88.5 (5.0)	80.4 (1.1)	53.9
172	Penthiopyrad	0.996	-11.0	9.9	5.7	9.0	0.7	2.8	-5.7	2.4	0.3	8.5	7.7	2.7	1.8	2.6	-5.5	6.9	93.2 (10.6)	82.8 (5.7)	70 (6.0)	0.4
173	Phenmedipham	0.999	-6.0	6.7	1.8	3.4	-2.7	1.3	-7.2	1.0	-1.1	4.6	0.3	3.1	-3.8	2.4	-6.5	4.3	104.5 (3.8)	90.7 (4.8)	78.6 (4.8)	-5.9
174	Phenthoate	0.998	-2.1	4.2	5.9	3.6	3.4	3.5	-4.2	3.4	-4.2	16.4	4.5	5.9	2.0	1.8	-7.5	7.5	92.3 (4.9)	84.1 (1.8)	75.6 (3.2)	-4.5
175	Phosalone	0.998	-16.6	7.5	6.7	5.1	0.2	2.0	-4.6	3.3	-13.6	10.9	6.1	7.6	0.6	4.1	-5.1	9.5	91.9 (10.6)	78.6 (8.8)	70.1 (5.7)	0.0
176	Phosmet	0.998	-8.2	5.1	2.6	3.0	−2.7	3.9	−5.9	2.2	−9.3	16.4	2.0	2.6	−2.2	3.1	−5.6	4.9	70.5 (3.4)	92.6 (8.4)	79 (3.1)	−2.0
177	Phosphamidon	1.000	12.4	4.3	0.5	2.9	−0.7	2.1	−3.7	1.2	0.0	4.6	0.7	3.4	−1.3	3.3	−4.1	3.6	109.9 (3.4)	93.7 (2.5)	84.8 (2.2)	−29.4
178	Phoxim	0.999	−8.1	5.3	4.6	8.3	−1.2	5.3	−8.7	0.9	−16.5	13.3	4.9	6.7	−5.0	3.6	−7.1	10.1	95 (14.6)	82.6 (7.2)	76.9 (6.4)	−1.7
179	Picolinafen	0.999	−1.6	4.9	1.0	5.4	−1.1	2.9	−8.4	1.3	−5.0	11.0	5.8	6.7	−2.7	1.8	−7.5	7.2	93.7 (3.5)	86.9 (9.4)	72.1 (3.9)	−1.3
180	Picoxystrobin	1.000	−0.6	4.8	−2.9	2.5	−3.2	3.0	−6.5	2.5	2.9	8.9	−1.7	4.4	−3.4	4.1	−5.1	6.3	87.8 (17.3)	88.7 (10.4)	80.7 (3.5)	−5.4
181	Piperophos	0.999	5.0	6.0	−0.3	7.5	−4.9	2.1	−7.0	3.0	3.0	12.3	−2.6	3.1	−1.3	5.4	−2.9	6.7	105.2 (13.0)	92.1 (7.5)	80.5 (4.8)	−1.7
182	Pirimicarb	1.000	6.1	3.5	1.2	2.2	0.6	1.8	−2.2	1.8	6.3	5.2	1.8	3.4	−0.9	2.3	−4.5	6.9	98.7 (2.3)	90.3 (8.5)	82.6 (4.6)	−22.7
183	Pirimiphos−ethyl	1.000	4.4	6.3	0.0	5.8	−4.8	1.9	−6.4	1.2	−0.6	9.1	−1.8	4.8	−4.4	1.8	−7.1	5.5	91.1 (5.0)	83.2 (9.2)	76 (5.8)	0.5
184	Pirimiphos−methyl	1.000	8.3	12.0	1.0	2.6	−3.6	1.7	−8.4	2.8	0.1	13.3	0.9	2.3	−4.0	6.4	−5.5	4.2	90.7 (10.3)	89 (12.4)	77.4 (4.2)	−3.7
185	Pretilachlor	0.999	0.8	5.0	−3.5	6.3	−4.7	2.5	−5.6	1.7	1.2	4.5	−0.7	5.3	−1.4	3.7	−2.1	8.5	75.3 (1.9)	75.6 (6.1)	69.3 (6.5)	−1.3
186	Probenazole	0.998	−16.5	10.2	5.2	7.4	6.3	3.5	1.7	2.8	1.9	18.8	4.2	8.1	−3.5	4.3	−5.1	6.4	104.6 (5.6)	92.1 (4.3)	84.9 (3.7)	−51.5
187	Prochloraz	1.000	−14.4	11.9	−8.5	5.8	−8.0	2.3	−8.6	2.4	−7.7	18.1	−1.1	8.8	−6.1	3.2	−5.0	5.5	101.9 (16.1)	86.1 (3.7)	78.3 (3.5)	1.1
188	Promecarb	0.997	−17.9	16.5	3.1	2.2	−1.6	3.2	−7.8	2.1	−6.7	16.4	6.8	5.0	−1.1	4.0	−6.7	6.7	93 (6.2)	97.3 (4.4)	81.8 (2.6)	−7.8
189	Prometryn	1.000	0.3	9.7	−1.9	2.8	−2.8	2.2	−5.8	2.6	−3.9	11.1	0.4	4.2	−3.5	4.2	−3.7	8.5	100.1 (7.5)	88.9 (7.4)	77.2 (2.8)	−2.8
190	Propachlor	0.998	−5.6	6.6	4.3	3.1	−0.3	1.5	−7.2	1.0	5.2	6.3	3.7	4.1	−2.0	3.4	−6.1	6.7	73 (4.4)	91.1 (8.8)	81.2 (3.2)	−9.7
191	Propazine	0.999	1.8	5.3	6.1	6.2	−1.6	2.8	−6.8	1.5	−6.7	7.8	3.7	2.8	−1.1	3.9	−4.8	4.8	85.6 (18.7)	86.8 (5.1)	77.8 (3.2)	−12.2
192	Propiconazole	1.000	6.9	8.7	2.1	3.8	−0.2	2.9	0.2	1.7	8.1	4.9	0.6	6.5	−4.3	3.9	−2.4	8.0	99.1 (14.9)	85.7 (10.2)	74.3 (5.7)	4.4
193	Propisochlor	0.997	−0.7	18.9	8.5	8.3	−5.2	3.7	−8.5	3.6	4.8	18.2	10.0	4.6	−2.6	5.3	−5.6	10.0	96 (18.6)	81.4 (1.7)	73.1 (4.3)	4.5
194	Propoxur	1.000	6.4	3.7	0.3	4.2	−0.9	2.9	−6.1	1.9	9.4	3.3	0.8	4.8	−1.9	2.8	−3.8	3.5	91.2 (8.1)	92.4 (1.2)	81.6 (1.3)	−30.0
195	Prothiofos	1.000	−3.0	7.2	−4.7	6.5	−5.4	4.2	−5.8	1.8	−3.2	8.8	−8.3	6.6	−3.6	4.4	−4.7	4.3	100.9 (4.8)	89.1 (9.3)	77.2 (10.0)	−2.7
196	Pyraclofos	1.000	9.1	6.5	−2.3	3.6	−7.0	3.6	−10.1	1.2	1.1	5.8	−2.6	6.1	1.7	4.5	0.4	12.9	91.4 (3.1)	80.8 (10.8)	76.5 (10.4)	−1.8
197	Pyraclostrobin	1.000	8.4	3.6	−1.8	5.1	−6.7	2.3	−8.4	2.9	3.1	13.0	−4.5	2.9	−3.6	3.0	−4.1	5.5	97.9 (6.5)	88.9 (3.0)	83.2 (4.2)	1.2
198	Pyrazolynate	0.998	4.0	13.5	3.7	7.0	−5.3	2.4	−9.9	3.1	4.3	14.4	7.2	8.1	0.8	7.7	−6.1	4.6	97 (10.4)	90.9 (16.5)	73.5 (3.9)	3.5
199	Pyrazophos	0.999	−2.4	12.8	2.1	10.7	−7.7	3.7	−10.2	5.3	3.6	18.0	−0.8	6.8	−5.1	8.1	−2.2	8.9	74 (15.7)	89.5 (2.0)	82.4 (8.6)	6.0
200	Pyrazoxyfen	1.000	−0.8	10.0	1.0	3.7	−2.5	2.3	−4.1	2.3	−4.6	11.0	−3.2	2.3	−2.8	3.0	−5.6	8.4	83.6 (2.7)	84.8 (8.6)	80.1 (8.3)	1.9
201	Pyribenzoxim	0.996	9.4	9.4	−14.3	13.2	−12.2	4.8	−11.7	4.8	−5.8	10.6	−11.5	12.4	−6.9	6.8	−2.2	11.2	92.2 (4.5)	71.8 (17.8)	85.8 (6.9)	1.7
202	Pyributicarb	1.000	4.0	11.1	−0.1	6.5	−3.9	1.9	−4.8	1.5	−0.4	14.9	0.4	4.9	−2.5	2.8	−4.3	7.8	89.6 (5.3)	79.2 (7.0)	74.9 (7.1)	0.5
203	Pyridaben	1.000	6.9	2.4	−0.9	6.7	−6.6	4.3	−6.5	3.6	1.8	6.3	−1.3	4.0	−4.4	4.9	−5.5	6.4	94.4 (6.2)	90.4 (6.9)	82.1 (5.2)	−5.0
204	Pyridalyl	0.999	4.6	2.8	−1.9	4.8	−3.9	2.4	−3.5	0.8	5.5	4.0	−1.2	5.2	−3.9	3.3	−4.2	9.0	83.7 (7.8)	75.9 (5.8)	70.5 (5.5)	2.7
205	Pyridaphenthion	0.997	−12.8	15.2	1.8	6.1	−3.7	5.0	−9.2	3.5	−7.4	9.0	7.2	4.8	−4.3	2.5	−9.2	6.6	94.9 (19.9)	92.9 (8.0)	74.8 (3.2)	10.6
206	Pyridate	0.999	6.8	3.8	−2.3	4.3	−4.3	2.7	−3.0	1.7	9.6	3.2	−2.4	3.6	−3.8	2.8	−1.3	7.8	98.9 (5.6)	80.9 (6.1)	71.8 (7.2)	−0.5
207	Pyrifenox	1.000	−3.8	8.9	4.9	10.8	−7.0	2.3	−9.6	4.1	−10.1	11.5	3.7	4.2	−5.9	3.3	−6.9	5.4	101 (9.9)	87.8 (5.7)	78.4 (5.0)	1.8
208	Pyriminobac-methyl E	1.000	14.6	3.6	0.4	3.4	−8.2	3.5	−8.5	3.3	1.8	6.5	−4.6	5.5	−7.1	3.1	−4.3	6.2	97 (10.1)	93.5 (10.0)	80.2 (1.5)	2.3
209	Pyriminobac-methyl Z	1.000	−0.9	3.8	−1.1	3.3	−5.9	2.6	−5.7	1.1	4.7	7.2	−3.4	3.8	−5.3	5.1	−4.9	5.3	103.9 (8.7)	94.6 (6.0)	82.7 (2.3)	−7.0
210	Pyrimisulfan	0.999	10.5	14.2	3.7	9.4	−1.6	9.2	−6.9	4.2	−2.5	12.2	3.7	6.0	2.5	6.2	−3.3	13.3	86 (2.9)	90.8 (13.0)	79.6 (5.4)	34.4
211	Pyriproxyfen	1.000	−2.2	3.5	2.5	4.8	−2.2	2.2	−5.0	2.8	−4.8	5.5	3.6	2.8	−0.6	2.8	−3.6	7.5	78.3 (3.8)	83.9 (4.9)	72.4 (4.1)	0.1
212	Pyroquilon	1.000	−10.5	6.1	4.2	2.9	2.9	1.1	−2.4	2.2	−2.3	14.1	0.7	5.8	−2.9	3.5	−5.0	4.2	82 (5.7)	89.1 (3.0)	81.2 (1.2)	−34.7
213	Quinoclamine	1.000	−3.1	13.6	−4.5	11.2	−0.6	4.3	−4.1	5.0	−1.4	18.7	−1.5	9.1	−3.7	7.3	−6.5	6.6	99.3 (19.6)	81.3 (11.3)	84.7 (3.0)	−51.1
214	Rimsulfuron	0.999	−4.8	10.2	2.3	2.7	−7.5	3.3	−9.5	2.8	−7.8	11.8	0.7	3.6	−5.9	3.4	−7.9	5.0	96.4 (2.7)	94.2 (6.0)	80.6 (7.2)	30.1
215	Saflufenacil	0.999	11.0	8.1	−3.8	6.4	−7.9	2.3	−11.5	4.9	−0.6	13.9	−5.2	8.4	−4.7	4.6	−5.9	9.0	93 (6.3)	85.1 (8.2)	75.4 (5.9)	63.2
216	Sethoxydim	0.999	−13.1	4.8	8.3	4.6	−2.4	3.8	−6.3	2.3	−3.8	16.5	5.4	2.9	−2.8	5.4	−5.9	7.4	85.8 (16.8)	85.8 (8.1)	71.1 (4.6)	−6.3
217	Simazine	0.998	−14.1	18.2	10.5	1.9	7.0	3.1	0.4	2.3	−1.6	17.3	2.3	5.9	1.9	3.0	−4.7	9.2	77.4 (7.1)	94.3 (7.9)	79.8 (5.5)	−34.8
218	Simeconazole	0.997	3.7	11.5	7.6	3.0	−0.6	2.7	−6.3	2.2	−10.3	11.2	5.0	5.5	−1.5	5.2	−7.6	7.7	87 (7.3)	82.3 (19.9)	76.9 (9.0)	3.8
219	Simetryn	0.999	2.3	7.4	−5.6	2.1	−3.3	2.4	−3.2	1.3	2.4	8.8	−3.0	2.8	−4.9	3.9	−3.8	5.9	101.8 (7.2)	91.9 (8.2)	86.1 (2.9)	−16.9
220	Spinetoram (XDE−175−J)	0.999	3.1	3.8	−5.2	6.5	−12.2	2.6	−7.7	2.9	7.9	4.2	−5.5	7.4	−6.3	4.6	−1.3	9.7	111.3 (6.9)	88 (9.2)	79.5 (5.1)	5.2
221	Spinosyn A	0.999	3.3	4.9	−4.2	6.7	−8.7	4.5	−6.8	3.4	6.0	9.8	−5.2	5.9	−4.3	5.8	−4.2	6.3	91.3 (8.7)	86 (11.7)	79.6 (5.0)	5.1
222	Spinosyn D	0.999	−6.4	3.7	−1.3	2.6	−5.3	3.1	−5.5	1.4	17.4	9.4	−3.3	8.3	−5.3	2.8	−4.2	6.6	106.2 (15.0)	86.2 (10.0)	79.1 (4.5)	2.5
223	Spirodiclofen	0.998	4.6	2.8	7.9	4.9	−3.0	8.5	−2.3	10.2	−8.2	8.7	0.3	7.8	−3.3	8.6	−10.6	8.2	80.2 (0.7)	78 (5.0)	74.5 (2.3)	−2.3
224	Sulfoxaflor	0.998	14.3	11.3	11.7	3.5	13.0	3.8	0.6	1.8	−2.6	8.3	7.0	3.2	3.0	10.3	−4.6	9.0	106.5 (16.3)	91.6 (5.6)	83.1 (4.5)	−42.5
225	Sulprofos	0.999	5.7	10.9	−3.0	4.2	−5.9	3.5	−7.2	1.6	4.3	11.8	−3.6	5.5	−6.8	3.4	−3.8	8.5	102.7 (9.1)	81.8 (5.4)	70.9 (7.0)	7.0
226	TCMTB	1.000	10.9	10.7	1.4	5.8	−6.6	4.3	−10.6	3.7	3.1	12.6	−0.6	5.3	−5.2	3.1	−8.8	5.4	81.9 (15.1)	91.6 (13.6)	79.2 (3.7)	−12.4
227	Tebuconazole	1.000	14.5	10.4	4.8	4.0	−1.7	2.1	−7.1	3.6	2.1	13.3	0.6	6.7	−5.1	4.2	−5.9	7.4	89.8 (2.8)	82.5 (12.6)	74.2 (3.3)	5.7
228	Tebufenozide	0.998	17.5	12.2	0.6	6.8	−2.4	4.2	−7.4	3.2	−12.4	13.0	0.5	4.8	−1.7	4.9	−5.5	8.2	111.3 (15.3)	84.8 (18.1)	80.8 (6.6)	8.8
229	Tebupirimfos	1.000	0.4	7.6	2.2	6.0	−6.6	2.9	−6.4	2.2	−1.4	9.5	2.0	4.3	−5.2	4.6	−6.3	8.3	99.2 (7.2)	88.9 (4.2)	78.1 (5.5)	−5.9
230	Terbuthylazine	0.998	−3.6	7.1	5.2	7.1	−1.1	3.4	−7.6	1.8	−1.9	5.6	6.2	3.6	−3.1	2.1	−10.6	4.5	86.8 (7.9)	94.8 (5.3)	82.4 (4.0)	−16.6
231	Terbutryn	1.000	−14.5	11.3	−5.4	0.8	−6.7	2.1	−7.9	3.2	1.9	15.6	−4.6	3.2	−6.7	2.9	−9.1	6.2	92 (3.3)	87.8 (6.5)	79.7 (1.6)	−1.1
232	Tetrachlorvinphos	1.000	3.1	15.3	6.8	6.2	0.7	4.9	−5.9	3.1	4.6	10.5	4.8	6.3	−0.2	5.7	−5.1	8.1	97.6 (14.8)	86.6 (11.8)	74 (4.4)	−2.5
233	Tetraconazole	0.998	10.7	10.0	−1.0	8.4	−1.7	4.4	−7.9	2.3	−10.5	14.2	4.5	6.5	−4.0	1.7	−5.5	4.2	72.4 (4.5)	87.9 (6.7)	76.4 (2.7)	−1.3
234	Thenylchlor	0.998	−8.6	6.2	8.6	4.1	−2.8	4.7	−8.6	4.3	−2.2	7.8	8.9	4.0	−2.7	1.7	−7.8	4.9	72.1 (9.1)	89.9 (8.7)	75 (6.2)	0.8
235	Thiabendazole	1.000	8.6	6.5	0.3	3.5	1.8	2.8	−2.6	2.4	9.5	7.2	−0.8	6.7	0.9	5.1	−3.1	5.5	98.6 (9.0)	87.3 (12.2)	85.7 (1.2)	−56.5
236	Thiacloprid	1.000	−18.3	14.8	3.7	5.8	6.7	3.3	−1.2	3.0	−5.5	13.3	4.3	4.0	3.1	4.1	−4.0	4.9	94.3 (6.3)	87.6 (6.9)	85.2 (2.7)	−41.1
237	Thiazopyr	0.996	4.2	14.4	7.0	5.4	−3.4	5.5	−6.4	2.0	10.4	13.6	4.7	6.0	−1.5	4.9	−8.8	12.1	92.4 (19.8)	74.8 (18.9)	73.1 (2.7)	−4.2
238	Thidiazuron	1.000	−6.6	10.5	3.4	1.5	−2.7	3.1	−4.9	2.0	0.6	10.0	3.0	2.1	−5.1	3.4	−6.0	3.3	81.7 (7.0)	89 (9.3)	79.4 (4.9)	5.1
239	Thifensulfuron−methyl	1.000	−1.4	4.5	2.6	4.9	−5.2	1.9	−5.7	1.7	6.0	12.0	0.6	4.8	−7.9	3.7	−4.5	4.3	89.6 (6.8)	94.4 (7.8)	81.8 (4.9)	38.0
240	Thiobencarb	0.998	1.5	8.1	0.7	3.8	−2.1	2.6	−10.8	2.7	0.2	6.5	2.8	5.9	−3.5	2.7	−6.5	6.1	71.9 (8.3)	85.3 (3.5)	74.7 (6.0)	−5.8
241	Thiodicarb	1.000	8.9	3.1	2.1	2.4	−1.4	3.4	−3.8	1.3	−1.9	10.5	0.2	5.1	−2.7	1.8	−6.1	5.8	95.7 (3.9)	94.2 (5.7)	83.1 (4.0)	−11.9
242	Thiophanate−methyl	1.000	2.6	3.9	2.5	3.9	−0.9	2.1	−0.8	2.2	6.7	8.1	0.8	5.9	−1.3	1.2	−4.7	5.1	103.1 (6.1)	91.4 (8.2)	84.5 (3.9)	−42.1
243	Tolfenpyrad	1.000	2.3	12.3	0.1	5.8	−7.6	2.7	−8.1	4.3	1.9	11.1	0.8	6.1	−3.5	5.9	−3.6	6.5	86.6 (6.0)	78 (8.2)	76.3 (9.2)	−2.8
244	Triadimefon	0.995	13.7	17.2	3.0	4.2	−4.3	3.7	−10.9	1.0	6.5	18.7	7.6	11.3	−5.6	6.4	−8.8	5.6	105.7 (13.1)	94.7 (5.0)	77.1 (5.2)	2.6
245	Triadimenol	0.999	12.5	18.7	−11.9	7.8	−2.6	2.2	−10.5	7.7	15.2	11.1	0.4	10.0	−2.2	6.0	−7.9	9.1	85 (13.0)	76.5 (16.0)	72.5 (8.0)	14.4
246	Tri−allate	0.998	−0.8	14.7	−4.5	8.1	−2.6	5.0	−5.5	3.1	−3.7	17.4	−1.3	13.3	−3.7	6.7	−2.2	10.0	110.6 (5.7)	82 (4.2)	76.9 (5.7)	−4.3
247	Triazophos	0.998	−4.7	6.7	2.2	7.4	−3.0	2.8	−8.9	1.8	−5.8	8.1	5.2	8.1	−2.7	4.3	−5.1	8.4	82.7 (1.2)	83.4 (4.4)	71.5 (5.6)	2.1
248	Tribenuron−methyl	1.000	−3.6	2.8	−0.7	1.6	−4.5	2.1	−8.1	1.7	−6.3	2.6	−0.1	2.2	−4.2	0.8	−5.2	5.2	95.7 (5.1)	89.2 (4.6)	71 (3.0)	−18.0
249	Tribufos	0.999	−0.2	2.6	0.4	1.5	−2.3	1.5	−5.8	0.9	−0.5	3.2	1.1	3.0	−3.5	2.5	−4.6	6.5	93.7 (5.3)	91 (7.9)	77.4 (2.3)	−1.4
250	Trichlorfon	0.999	10.5	4.7	2.6	7.3	2.4	4.9	−3.8	2.1	0.6	15.6	2.2	5.2	−1.5	4.5	−4.6	3.6	71.8 (15.5)	88.8 (7.3)	81.1 (5.2)	−32.1
251	Tricyclazole	1.000	14.6	15.1	3.1	7.8	5.6	2.1	−0.3	4.0	11.7	6.9	−1.3	8.2	4.5	3.8	−3.1	6.6	110.9 (7.2)	93 (8.8)	89.4 (2.8)	−52.1
252	Trifloxystrobin	0.999	−5.0	5.3	−0.2	5.8	−7.1	1.8	−9.1	2.7	−3.5	13.6	4.0	6.6	−2.2	4.9	−6.3	5.5	81.5 (3.8)	89.5 (8.4)	78.4 (6.6)	−5.9
253	Triflumizole	0.999	12.9	10.1	−1.8	5.3	−5.4	2.3	−6.5	1.3	1.2	17.8	−4.1	4.8	−3.0	5.1	−3.2	7.8	90.7 (11.7)	83 (6.0)	76.9 (10.2)	−4.0
254	Triflumuron	0.996	−8.3	9.6	10.2	7.2	1.1	4.1	−9.1	3.6	−13.0	8.6	5.5	4.6	1.0	5.6	−5.0	10.2	85.9 (7.8)	91.1 (4.2)	74.7 (8.7)	−5.8
255	Trimethacarb	0.999	−5.8	5.4	3.0	1.7	−1.3	2.1	−7.3	0.8	−6.4	7.3	5.0	2.4	−1.8	2.1	−6.7	4.3	93.2 (5.0)	94.5 (6.6)	80.9 (1.4)	−11.9
256	Triticonazole	0.999	−6.0	10.4	0.7	1.9	−3.9	4.8	−8.2	2.8	−4.5	10.5	3.5	6.5	−5.0	3.0	−7.0	10.4	83 (5.4)	85.9 (9.8)	75.6 (6.6)	16.2
257	Uniconazole	0.997	−10.5	13.2	0.6	6.5	−2.4	5.6	−8.9	2.3	−2.7	10.3	4.8	2.9	−3.5	2.8	−8.3	5.9	96.5 (11.4)	88.3 (4.5)	73.4 (3.1)	7.0
258	Vamidothion	1.000	16.5	5.0	5.2	2.2	−0.5	3.3	−2.9	2.0	7.3	8.6	0.2	5.5	−3.6	1.7	−3.9	4.4	83 (10.8)	88.1 (4.4)	85 (6.8)	−36.7
259	XMC	1.000	7.3	6.4	4.4	2.8	1.7	1.8	−0.9	3.0	1.3	11.5	2.5	4.4	−1.4	3.6	−3.5	6.6	95.2 (5.8)	90 (7.6)	81.2 (5.9)	−21.4
260	Zoxamide	0.999	8.6	7.4	3.1	3.6	−5.2	4.9	−9.3	1.8	1.2	14.7	2.4	3.1	−4.0	5.6	−6.1	6.7	96.7 (6.6)	85.7 (9.3)	76.8 (2.0)	−4.4

^1^ Matrix effect.

**Table 5:** Quantitative application results in urine samples obtained from agricultural workers.

Compound Name	#4 ng/mL	#5 ng/mL	#6 ng/mL	#7 ng/mL	#10 ng/mL
Imidacloprid	11.7	-^1^	<LOQ	10.8	-
Difenoconazole	-	15.3	-	-	-
Chlorfluazuron	-	-	-	-	<LOQ

^1^ Not detected.

## Supplementary Materials

The following are available online at https://www.mdpi.com/1420-3049/24/7/1330/s1, Table S1: Recoveries and relative standard deviations (RSDs) of 260 pesticides for three versions of urine preparation methods; Table S2: Relative area intensities (100 at solvent standard peak area) of 260 pesticides for three versions of urine preparation methods; Figure S1: Matrix effects of 260 pesticides in 100 µL and 400 µL of urine samples.
