# Supplementary material for: A Quantitative Tandem Mass Spectrometry and Scaled-Down QuEChERS Approach for Simultaneous Analysis of Pesticide Multiresidues in Human Urine
Source: Molecules. 2019 Apr 4;24(7):1330. doi: 10.3390/molecules24071330 (PMC6480104; doi:10.3390/molecules24071330)
Supplement: Supplementary file 1 [file molecules-24-01330-s001.pdf]

*Supplementary Material*

# A Quantitative Tandem Mass Spectrometry and Scaled-Down QuEChERS Approach for Simultaneous Analysis of Pesticide Multiresidues in Human Urine

**Yongho Shin [1,2], Jiho Lee [1], Eunyoung Park [1], Junghak Lee [1], Hye Suk Lee [2,*] and Jeong-Han Kim [1,*]**

[1] Pesticide Chemistry and Toxicology Laboratory, Department of Agricultural Biotechnology and Research Institute of Agriculture and Life Sciences, Seoul National University, Seoul 08826, Republic of Korea; driger6103@catholic.ac.kr (Y.S.); micai1@snu.ac.kr (J.L.); pey4321@snu.ac.kr (E.P.); crane245@snu.ac.kr (J.L.)
[2] Drug Metabolism and Bioanalysis Laboratory, College of Pharmacy, The Catholic University of Korea, Bucheon-si, Gyeonggi-do 14662, Republic of Korea
* Correspondence: kjh2404@snu.ac.kr; Tel.: +82-2-880-4644; Fax: +82-2-873-4415 (J.-H.K.); sianalee@catholic.ac.kr; Tel.: +82-2-2164-4061; Fax: +82-32-342-2013 (H.S.L.)

**Table S1.** Recoveries and relative standard deviations (RSDs) of 260 pesticides for three versions of urine preparation methods ($n$ = 3).

| No. | Compound name | Method A | | Method B | | Method C | |
|---|---|---|---|---|---|---|---|
| | | RCY[1] (%) | RSD (%) | RCY (%) | RSD (%) | RCY (%) | RSD (%) |
| 1 | Acetamiprid | 89.2 | 6.5 | 85.1 | 4.5 | 84.2 | 4.2 |
| 2 | Alachlor | 87.7 | 1.9 | 85.6 | 2.9 | 80.7 | 0.7 |
| 3 | Allidochlor | 105.7 | 6.3 | 96.0 | 5.9 | 94.4 | 3.7 |
| 4 | Ametoctradin | 90.0 | 2.7 | 85.6 | 1.6 | 84.3 | 3.6 |
| 5 | Ametryn | 95.4 | 1.3 | 83.4 | 1.7 | 86.0 | 2.8 |
| 6 | Amisulbrom | 84.8 | 0.9 | 100.6 | 4.9 | 70.1 | 11.9 |
| 7 | Anilofos | 94.8 | 1.3 | 86.9 | 1.5 | 82.3 | 1.9 |
| 8 | Asulam | 87.4 | 2.5 | 75.2 | 5.9 | 87.3 | 13.4 |
| 9 | Atrazine | 96.7 | 5.2 | 89.8 | 5.0 | 85.4 | 6.2 |
| 10 | Azaconazole | 95.3 | 0.8 | 89.9 | 2.5 | 90.2 | 1.2 |
| 11 | Azamethiphos | 94.9 | 3.0 | 82.7 | 3.6 | 85.8 | 6.1 |
| 12 | Azimsulfuron | 98.2 | 2.6 | 86.7 | 4.6 | 86.9 | 3.0 |
| 13 | Azinphos-methyl | 96.0 | 5.3 | 91.2 | 4.8 | 94.8 | 2.5 |
| 14 | Azoxystrobin | 89.3 | 2.5 | 87.0 | 1.4 | 87.7 | 5.8 |
| 15 | Bendiocarb | 89.9 | 2.0 | 84.5 | 1.5 | 80.6 | 8.4 |
| 16 | Bensulfuron-methyl | 92.2 | 2.1 | 91.9 | 5.4 | 86.7 | 2.4 |
| 17 | Bentazone | 93.9 | 8.1 | 68.8 | 6.7 | 83.1 | 6.3 |
| 18 | Benthiavalicarb-isopropyl | 96.4 | 3.7 | 92.2 | 0.5 | 82.3 | 5.3 |
| 19 | Benzobicyclon | 88.1 | 3.1 | 88.9 | 5.9 | 77.3 | 3.4 |
| 20 | Benzoximate | 94.2 | 5.5 | 81.7 | 2.7 | 85.1 | 7.0 |
| 21 | Boscalid | 92.4 | 2.3 | 87.1 | 8.6 | 78.5 | 2.7 |
| 22 | Bromacil | 90.9 | 2.2 | 90.0 | 1.2 | 83.9 | 5.6 |
| 23 | Bromobutide | 97.1 | 2.8 | 86.6 | 2.2 | 84.7 | 0.9 |
| 24 | Bupirimate | 86.4 | 1.3 | 81.8 | 3.3 | 84.5 | 3.8 |
| 25 | Buprofezin | 87.5 | 4.8 | 90.3 | 4.6 | 90.4 | 1.8 |
| 26 | Butachlor | 90.8 | 2.4 | 86.4 | 0.3 | 81.3 | 0.7 |
| 27 | Butafenacil | 84.1 | 3.6 | 83.5 | 2.5 | 83.1 | 6.7 |

| No. | Compound name | Method A | | Method B | | Method C | |
|---|---|---|---|---|---|---|---|
| | | RCY[1] (%) | RSD (%) | RCY (%) | RSD (%) | RCY (%) | RSD (%) |
| 28 | Cadusafos | 91.1 | 4.1 | 89.5 | 6.5 | 83.0 | 4.2 |
| 29 | Carbaryl | 89.9 | 2.3 | 91.6 | 3.5 | 83.3 | 4.3 |
| 30 | Carbendazim | 89.0 | 1.8 | 87.2 | 1.7 | 80.9 | 3.0 |
| 31 | Carbofuran | 94.2 | 6.1 | 83.4 | 3.6 | 92.1 | 2.6 |
| 32 | Carbophenothion | 94.3 | 1.8 | 86.4 | 4.0 | 89.9 | 0.5 |
| 33 | Carboxin | 93.1 | 3.9 | 91.7 | 6.9 | 78.0 | 3.8 |
| 34 | Carpropamid | 102.6 | 4.1 | 100.3 | 7.8 | 93.4 | 0.4 |
| 35 | Cartap | 89.9 | 8.7 | 80.0 | 8.0 | N.D.[2] | N.D. |
| 36 | Chlorantraniliprole | 86.7 | 2.8 | 93.6 | 6.2 | 89.9 | 3.7 |
| 37 | Chlorfenvinphos | 91.3 | 6.6 | 87.2 | 5.2 | 83.3 | 6.7 |
| 38 | Chlorfluazuron | 89.6 | 2.6 | 91.6 | 5.0 | 85.3 | 5.5 |
| 39 | Chloridazon | 91.0 | 2.4 | 92.4 | 10.2 | 76.9 | 11.9 |
| 40 | Chlorotoluron | 96.0 | 1.1 | 86.9 | 5.2 | 83.9 | 2.9 |
| 41 | Chlorsulfuron | 100.8 | 4.1 | 91.3 | 4.8 | 92.0 | 5.1 |
| 42 | Chromafenozide | 93.3 | 8.8 | 89.4 | 5.1 | 83.9 | 9.5 |
| 43 | Cinmethylin | 97.6 | 3.6 | 102.8 | 4.6 | 84.4 | 3.2 |
| 44 | Clofentezine | 103.3 | 1.6 | 87.1 | 3.3 | 89.6 | 3.7 |
| 45 | Clomazone | 89.7 | 1.3 | 84.6 | 5.5 | 83.9 | 3.9 |
| 46 | Clothianidin | 91.0 | 2.6 | 80.7 | 3.1 | 81.0 | 16.5 |
| 47 | Cyanazine | 92.2 | 4.5 | 89.4 | 3.8 | 80.6 | 2.5 |
| 48 | Cyazofamid | 90.4 | 3.5 | 91.9 | 5.3 | 90.5 | 6.5 |
| 49 | Cyclosulfamuron | 86.0 | 1.1 | 93.2 | 4.9 | 92.2 | 4.5 |
| 50 | Cymoxanil | 103.2 | 6.4 | 82.1 | 5.9 | 80.4 | 4.9 |
| 51 | Cyproconazole | 101.7 | 2.4 | 90.6 | 5.8 | 81.6 | 2.1 |
| 52 | Cyprodinil | 94.7 | 5.1 | 83.9 | 2.1 | 90.9 | 6.9 |
| 53 | Daimuron | 94.9 | 4.7 | 86.2 | 4.2 | 84.2 | 12.6 |
| 54 | Diazinon | 86.7 | 2.6 | 92.3 | 3.3 | 86.6 | 3.4 |
| 55 | Dicrotophos | 90.7 | 0.3 | 85.6 | 2.2 | 82.9 | 0.8 |
| 56 | Diethofencarb | 96.3 | 1.7 | 83.5 | 0.2 | 81.5 | 1.4 |
| 57 | Difenoconazole | 95.1 | 1.8 | 87.9 | 3.3 | 86.6 | 4.1 |
| 58 | Diflubenzuron | 87.0 | 6.8 | 87.5 | 3.3 | 82.2 | 7.8 |
| 59 | Diflufenican | 107.2 | 1.0 | 87.5 | 1.3 | 84.0 | 9.1 |
| 60 | Dimethachlor | 89.1 | 3.7 | 88.4 | 1.9 | 85.4 | 3.2 |
| 61 | Dimethametryn | 86.5 | 3.7 | 80.3 | 4.5 | 80.9 | 4.2 |
| 62 | Dimethenamid | 90.1 | 1.5 | 86.4 | 0.8 | 89.9 | 2.3 |
| 63 | Dimethoate | 91.6 | 4.3 | 84.7 | 2.6 | 89.7 | 7.9 |
| 64 | Dimethomorph | 88.5 | 3.6 | 84.6 | 2.0 | 89.0 | 2.7 |
| 65 | Diniconazole | 91.5 | 1.0 | 93.8 | 2.5 | 87.0 | 4.7 |
| 66 | Diphenamid | 91.7 | 1.4 | 85.2 | 0.6 | 78.1 | 2.4 |
| 67 | Diuron | 94.4 | 2.3 | 80.4 | 3.1 | 82.2 | 3.4 |
| 68 | Edifenphos | 87.5 | 4.3 | 85.5 | 3.7 | 79.2 | 3.6 |
| 69 | Emamectin B1a | 91.2 | 1.9 | 86.8 | 1.3 | 83.9 | 1.7 |
| 70 | Emamectin B1b | 89.9 | 7.5 | 89.8 | 3.1 | 83.2 | 4.4 |
| 71 | EPN | 95.5 | 5.3 | 84.0 | 3.7 | 83.5 | 4.6 |
| 72 | Epoxiconazole | 101.3 | 3.4 | 93.3 | 1.9 | 86.2 | 5.5 |
| 73 | Ethaboxam (EBX) | 89.1 | 5.9 | 86.5 | 3.4 | 82.8 | 4.8 |
| 74 | Ethametsulfuron-methyl | 89.2 | 4.4 | 82.3 | 3.2 | 82.3 | 1.7 |
| 75 | Ethiofencarb | 95.3 | 1.1 | 81.3 | 5.4 | 86.8 | 1.8 |

| No. | Compound name | Method A | | Method B | | Method C | |
|---|---|---|---|---|---|---|---|
| | | RCY[1] (%) | RSD (%) | RCY (%) | RSD (%) | RCY (%) | RSD (%) |
| 76 | Ethion | 90.7 | 2.3 | 87.5 | 1.4 | 85.2 | 1.4 |
| 77 | Ethoprophos | 89.2 | 0.3 | 91.8 | 2.7 | 81.2 | 3.7 |
| 78 | Ethoxyquin | 88.0 | 6.5 | 89.7 | 6.7 | 90.0 | 7.3 |
| 79 | Ethoxysulfuron | 87.4 | 3.4 | 91.3 | 4.8 | 81.1 | 7.0 |
| 80 | Etoxazole | 82.6 | 1.6 | 88.2 | 4.3 | 78.7 | 3.5 |
| 81 | Etrimfos | 87.9 | 1.5 | 79.3 | 5.4 | 80.8 | 5.6 |
| 82 | Fenamidone | 91.7 | 5.2 | 85.0 | 6.3 | 83.4 | 7.7 |
| 83 | Fenamiphos | 92.6 | 3.2 | 85.6 | 2.3 | 84.5 | 1.8 |
| 84 | Fenazaquin | 86.4 | 0.7 | 89.6 | 2.6 | 86.1 | 3.7 |
| 85 | Fenbuconazole | 107.3 | 5.9 | 93.5 | 5.9 | 84.6 | 3.6 |
| 86 | Fenhexamid | 90.3 | 5.5 | 92.0 | 10.8 | 85.0 | 5.0 |
| 87 | Fenobucarb (BPMC) | 95.9 | 2.2 | 85.3 | 0.2 | 84.0 | 5.3 |
| 88 | Fenothiocarb | 93.3 | 5.3 | 88.5 | 2.6 | 91.5 | 7.3 |
| 89 | Fenoxanil | 97.9 | 2.2 | 91.4 | 1.9 | 89.0 | 2.0 |
| 90 | Fenoxycarb | 96.0 | 3.0 | 87.2 | 5.7 | 85.9 | 2.4 |
| 91 | Fenthion | 96.5 | 6.3 | 88.9 | 1.4 | 83.9 | 4.1 |
| 92 | Ferimzone | 83.3 | 1.4 | 77.4 | 5.5 | 76.5 | 2.9 |
| 93 | Fipronil | 88.4 | 4.8 | 93.7 | 1.9 | 89.4 | 2.3 |
| 94 | Fluacrypyrim | 94.8 | 3.1 | 78.2 | 2.7 | 85.6 | 3.2 |
| 95 | Fluazinam | 79.6 | 2.0 | 83.4 | 3.2 | 91.3 | 6.8 |
| 96 | Flucetosulfuron | 89.5 | 5.5 | 93.2 | 2.5 | 88.3 | 2.5 |
| 97 | Flufenacet | 98.7 | 2.7 | 92.6 | 3.2 | 83.5 | 8.1 |
| 98 | Flufenoxuron | 86.4 | 2.4 | 87.4 | 1.8 | 86.9 | 4.6 |
| 99 | Fluopicolide | 88.2 | 7.9 | 96.7 | 4.6 | 87.7 | 11.0 |
| 100 | Fluopyram | 94.8 | 2.1 | 99.5 | 4.7 | 85.4 | 6.3 |
| 101 | Flusilazole | 93.4 | 1.2 | 87.0 | 4.2 | 86.6 | 2.9 |
| 102 | Flusulfamide | 96.0 | 2.5 | 88.2 | 4.0 | 84.6 | 4.9 |
| 103 | Flutolanil | 89.1 | 1.6 | 76.7 | 4.3 | 88.3 | 4.5 |
| 104 | Fluxapyroxad | 102.4 | 7.5 | 121.0 | 8.8 | 83.2 | 4.6 |
| 105 | Fonofos | 100.8 | 0.9 | 94.6 | 4.0 | 96.4 | 2.9 |
| 106 | Forchlorfenuron | 84.0 | 5.1 | 84.3 | 1.4 | 85.4 | 3.1 |
| 107 | Fosthiazate | 88.7 | 1.1 | 85.3 | 3.4 | 83.5 | 3.2 |
| 108 | Furathiocarb | 91.8 | 3.7 | 83.3 | 3.2 | 81.8 | 1.4 |
| 109 | Halosulfuron-methyl | 87.6 | 4.7 | 89.1 | 3.0 | 90.5 | 1.4 |
| 110 | Haloxyfop-R-Methyl | 96.3 | 3.4 | 95.6 | 4.3 | 84.8 | 6.0 |
| 111 | Hexaconazole | 97.4 | 4.6 | 88.8 | 0.6 | 85.0 | 4.3 |
| 112 | Hexaflumuron | 85.0 | 13.0 | 82.0 | 4.5 | 82.4 | 4.2 |
| 113 | Hexazinone | 89.0 | 5.8 | 86.6 | 5.0 | 81.0 | 2.6 |
| 114 | Imazalil | 96.3 | 7.4 | 90.7 | 5.5 | 79.5 | 7.7 |
| 115 | Imazapic | 56.1 | 1.3 | 49.4 | 6.3 | 82.7 | 1.6 |
| 116 | Imazaquin | 89.0 | 3.3 | 73.9 | 2.1 | 81.4 | 8.5 |
| 117 | Imazethapyr | 74.2 | 9.7 | 77.5 | 8.0 | 72.7 | 2.2 |
| 118 | Imibenconazole | 94.5 | 2.3 | 96.8 | 2.6 | 86.7 | 3.0 |
| 119 | Imicyafos | 84.5 | 6.0 | 90.8 | 11.6 | 99.3 | 12.8 |
| 120 | Imidacloprid | 86.1 | 2.5 | 85.9 | 5.1 | 83.8 | 3.3 |
| 121 | Indoxacarb | 84.3 | 5.6 | 82.5 | 8.8 | 83.0 | 6.1 |
| 122 | Iprobenfos | 89.5 | 2.4 | 83.3 | 4.8 | 81.0 | 4.9 |
| 123 | Iprovalicarb | 87.9 | 4.7 | 87.6 | 5.9 | 88.4 | 2.5 |

| No. | Compound name | Method A | | Method B | | Method C | |
|---|---|---|---|---|---|---|---|
| | | RCY[1] (%) | RSD (%) | RCY (%) | RSD (%) | RCY (%) | RSD (%) |
| 124 | Isazofos | 87.3 | 2.1 | 87.4 | 2.8 | 87.0 | 4.4 |
| 125 | Isoprocarb | 104.8 | 4.7 | 90.8 | 3.1 | 85.5 | 1.3 |
| 126 | Isoprothiolane | 86.9 | 5.4 | 85.5 | 2.0 | 91.7 | 3.8 |
| 127 | Isoproturon | 92.2 | 4.6 | 86.8 | 4.7 | 86.2 | 2.2 |
| 128 | Isopyrazam | 82.9 | 4.1 | 87.8 | 3.3 | 86.5 | 3.0 |
| 129 | Isoxathion | 87.3 | 3.6 | 93.0 | 2.9 | 86.0 | 4.2 |
| 130 | Kresoxim-methyl | 84.6 | 8.0 | 80.8 | 10.8 | 83.6 | 3.0 |
| 131 | Linuron | 96.8 | 5.8 | 92.3 | 4.7 | 84.0 | 0.9 |
| 132 | Mandipropamid | 84.8 | 2.4 | 86.5 | 4.9 | 90.6 | 7.7 |
| 133 | Mecarbam | 90.7 | 4.8 | 88.0 | 6.0 | 81.7 | 6.6 |
| 134 | Mefenacet | 89.2 | 5.7 | 85.5 | 2.6 | 84.3 | 5.4 |
| 135 | Mefenpyr-diethyl | 100.5 | 4.0 | 88.3 | 2.1 | 90.2 | 0.9 |
| 136 | Mepronil | 94.1 | 8.2 | 90.5 | 4.4 | 84.4 | 4.9 |
| 137 | Metalaxyl | 91.8 | 0.6 | 85.2 | 2.3 | 85.3 | 2.6 |
| 138 | Metamifop | 90.3 | 2.6 | 93.0 | 4.6 | 87.8 | 2.6 |
| 139 | Metazosulfuron | 91.7 | 7.7 | 89.2 | 1.4 | 82.1 | 6.1 |
| 140 | Metconazole | 97.8 | 2.0 | 85.5 | 3.9 | 90.8 | 3.8 |
| 141 | Methabenzthiazuron | 91.9 | 4.5 | 88.3 | 4.6 | 82.0 | 4.5 |
| 142 | Methidathion | 87.9 | 2.6 | 79.7 | 8.6 | 84.0 | 4.9 |
| 143 | Methiocarb | 99.2 | 2.4 | 90.4 | 5.2 | 83.9 | 4.8 |
| 144 | Methomyl | 98.2 | 4.0 | 76.2 | 10.7 | 88.7 | 1.5 |
| 145 | Methoxyfenozide | 89.2 | 9.8 | 87.8 | 4.7 | 91.7 | 4.2 |
| 146 | Metobromuron | 102.7 | 8.6 | 80.2 | 5.5 | 84.9 | 4.7 |
| 147 | Metolachlor | 88.6 | 2.6 | 85.9 | 1.3 | 85.2 | 0.6 |
| 148 | Metolcarb | 111.1 | 4.1 | 81.6 | 3.7 | 87.5 | 2.4 |
| 149 | Metominostrobin | 101.7 | 4.0 | 89.0 | 2.1 | 82.3 | 3.4 |
| 150 | Metrafenone | 96.3 | 2.5 | 87.5 | 2.8 | 89.7 | 2.7 |
| 151 | Mevinphos | 91.8 | 1.7 | 87.9 | 9.5 | 82.3 | 1.2 |
| 152 | Milbemectin A4 | 102.4 | 2.6 | 86.1 | 1.3 | 75.8 | 1.7 |
| 153 | Molinate | 88.2 | 2.1 | 87.2 | 5.3 | 84.7 | 8.2 |
| 154 | Monocrotophos | 90.1 | 0.4 | 83.6 | 5.3 | 81.2 | 5.9 |
| 155 | Myclobutanil | 100.8 | 7.8 | 95.2 | 4.2 | 81.5 | 4.0 |
| 156 | Napropamide | 98.3 | 4.5 | 85.7 | 2.7 | 77.0 | 4.6 |
| 157 | Nicosulfuron | 79.3 | 3.3 | 84.0 | 2.5 | 86.8 | 2.8 |
| 158 | Nitenpyram | 63.1 | 2.8 | 66.4 | 15.4 | 74.5 | 3.9 |
| 159 | Nuarimol | 92.8 | 6.3 | 87.8 | 8.8 | 84.4 | 9.0 |
| 160 | Ofurace | 86.2 | 1.9 | 85.4 | 7.8 | 92.4 | 5.2 |
| 161 | Omethoate | 73.0 | 7.4 | 83.8 | 3.4 | 89.6 | 7.3 |
| 162 | Orysastrobin | 103.8 | 8.4 | 90.1 | 1.8 | 90.0 | 6.7 |
| 163 | Oxadiazon | 103.2 | 2.5 | 84.1 | 2.3 | 88.2 | 1.4 |
| 164 | Oxadixyl | 89.0 | 4.1 | 84.4 | 0.8 | 81.1 | 4.2 |
| 165 | Oxamyl | 85.7 | 2.0 | 84.3 | 4.3 | 88.5 | 1.0 |
| 166 | Oxydemeton-methyl | 74.1 | 1.4 | 80.5 | 4.3 | 80.6 | 3.9 |
| 167 | Paclobutrazol | 99.3 | 4.9 | 82.7 | 2.8 | 84.0 | 3.1 |
| 168 | Pebulate | 98.8 | 4.9 | 83.3 | 5.6 | 87.7 | 0.9 |
| 169 | Penconazole | 92.2 | 1.9 | 87.0 | 3.1 | 81.5 | 2.2 |
| 170 | Pendimethalin | 93.3 | 2.9 | 91.9 | 1.5 | 87.8 | 4.7 |
| 171 | Penoxsulam | 93.2 | 3.5 | 88.3 | 0.6 | 81.8 | 4.9 |

| No. | Compound name | Method A | | Method B | | Method C | |
|---|---|---|---|---|---|---|---|
| | | RCY[1] (%) | RSD (%) | RCY (%) | RSD (%) | RCY (%) | RSD (%) |
| 172 | Penthiopyrad | 97.4 | 0.5 | 86.8 | 7.0 | 86.4 | 0.7 |
| 173 | Phenmedipham | 92.1 | 5.3 | 84.1 | 4.6 | 86.5 | 4.1 |
| 174 | Phenthoate | 90.3 | 2.9 | 80.7 | 2.9 | 82.8 | 5.3 |
| 175 | Phosalone | 90.6 | 2.6 | 92.5 | 2.4 | 89.5 | 0.8 |
| 176 | Phosmet | 90.2 | 1.7 | 85.1 | 3.7 | 89.0 | 3.1 |
| 177 | Phosphamidon | 90.0 | 6.2 | 86.5 | 2.4 | 82.5 | 1.2 |
| 178 | Phoxim | 108.7 | 1.3 | 84.6 | 3.9 | 81.7 | 3.1 |
| 179 | Picolinafen | 89.3 | 3.0 | 90.3 | 2.0 | 85.3 | 2.3 |
| 180 | Picoxystrobin | 92.1 | 4.9 | 87.9 | 3.5 | 85.7 | 6.0 |
| 181 | Piperophos | 93.6 | 1.8 | 85.5 | 5.1 | 79.6 | 5.6 |
| 182 | Pirimicarb | 88.8 | 1.3 | 86.5 | 4.6 | 82.9 | 3.5 |
| 183 | Pirimiphos-ethyl | 95.7 | 3.1 | 85.9 | 2.3 | 84.5 | 1.0 |
| 184 | Pirimiphos-methyl | 95.1 | 3.1 | 87.7 | 3.9 | 83.2 | 6.7 |
| 185 | Pretilachlor | 95.7 | 0.7 | 92.0 | 3.0 | 92.0 | 0.9 |
| 186 | Probenazole | 105.4 | 3.7 | 89.3 | 2.9 | 82.6 | 7.1 |
| 187 | Prochloraz | 91.3 | 3.0 | 88.6 | 4.5 | 88.2 | 5.0 |
| 188 | Promecarb | 97.6 | 3.1 | 93.4 | 3.3 | 84.6 | 1.2 |
| 189 | Prometryn | 88.2 | 1.5 | 83.5 | 4.8 | 79.1 | 8.8 |
| 190 | Propachlor | 100.6 | 4.0 | 93.5 | 5.1 | 80.4 | 2.0 |
| 191 | Propazine | 99.4 | 5.1 | 85.3 | 6.4 | 86.4 | 3.3 |
| 192 | Propiconazole | 89.5 | 2.5 | 93.0 | 1.0 | 85.1 | 1.9 |
| 193 | Propisochlor | 94.5 | 4.4 | 85.6 | 2.1 | 89.5 | 5.2 |
| 194 | Propoxur | 93.3 | 2.0 | 87.3 | 8.0 | 84.2 | 7.1 |
| 195 | Prothiofos | 104.8 | 4.1 | 95.0 | 4.5 | 88.2 | 5.6 |
| 196 | Pyraclofos | 105.7 | 5.9 | 83.5 | 6.1 | 79.7 | 6.6 |
| 197 | Pyraclostrobin | 89.8 | 2.3 | 88.2 | 3.2 | 85.5 | 5.8 |
| 198 | Pyrazolynate | 93.2 | 4.9 | 96.4 | 7.1 | 82.4 | 1.9 |
| 199 | Pyrazophos | 95.5 | 4.0 | 83.4 | 7.0 | 84.5 | 8.1 |
| 200 | Pyrazoxyfen | 91.1 | 0.6 | 97.7 | 0.7 | 92.3 | 4.4 |
| 201 | Pyribenzoxim | 82.6 | 2.2 | 86.4 | 6.8 | 76.2 | 6.6 |
| 202 | Pyributicarb | 94.8 | 1.3 | 84.7 | 1.0 | 82.3 | 2.3 |
| 203 | Pyridaben | 95.1 | 2.9 | 84.5 | 5.1 | 82.3 | 2.1 |
| 204 | Pyridalyl | 91.6 | 1.4 | 86.0 | 0.3 | 86.1 | 1.8 |
| 205 | Pyridaphenthion | 90.4 | 7.1 | 85.9 | 5.0 | 86.4 | 3.5 |
| 206 | Pyridate | 95.3 | 2.9 | 81.5 | 2.8 | 83.2 | 1.6 |
| 207 | Pyrifenox | 103.1 | 3.8 | 85.0 | 1.4 | 87.0 | 4.8 |
| 208 | Pyriminobac-methyl E | 87.9 | 4.0 | 87.8 | 2.4 | 84.8 | 1.0 |
| 209 | Pyriminobac-methyl Z | 96.2 | 4.3 | 88.0 | 4.0 | 82.3 | 2.9 |
| 210 | Pyrimisulfan | 84.0 | 9.0 | 105.8 | 8.6 | 91.5 | 12.7 |
| 211 | Pyriproxyfen | 87.3 | 3.1 | 90.5 | 3.2 | 90.9 | 0.8 |
| 212 | Pyroquilon | 94.8 | 3.5 | 83.0 | 4.2 | 85.7 | 1.5 |
| 213 | Quinoclamine | 91.9 | 3.6 | 87.1 | 0.7 | 72.6 | 21.3 |
| 214 | Rimsulfuron | 93.6 | 4.0 | 79.6 | 3.0 | 85.2 | 1.6 |
| 215 | Saflufenacil | 84.2 | 4.0 | 86.6 | 4.0 | 86.8 | 6.2 |
| 216 | Sethoxydim | 92.8 | 1.4 | 83.9 | 4.0 | 79.9 | 1.8 |
| 217 | Simazine | 101.7 | 1.0 | 89.2 | 4.7 | 92.4 | 5.4 |
| 218 | Simeconazole | 83.3 | 2.5 | 88.8 | 3.9 | 79.8 | 5.5 |
| 219 | Simetryn | 92.7 | 1.9 | 84.4 | 3.6 | 79.9 | 3.3 |

| No. | Compound name | Method A | | Method B | | Method C | |
|---|---|---|---|---|---|---|---|
| | | RCY[1] (%) | RSD (%) | RCY (%) | RSD (%) | RCY (%) | RSD (%) |
| 220 | Spinetoram (XDE-175-J) | 93.7 | 1.8 | 93.2 | 7.1 | 85.3 | 5.6 |
| 221 | Spinosyn A | 89.2 | 4.4 | 80.6 | 3.0 | 81.0 | 1.7 |
| 222 | Spinosyn D | 93.7 | 1.7 | 88.7 | 4.1 | 85.9 | 0.9 |
| 223 | Spirodiclofen | 92.0 | 2.6 | 86.0 | 3.5 | 86.0 | 2.9 |
| 224 | Sulfoxaflor | 85.9 | 2.7 | 87.8 | 5.1 | 91.4 | 17.7 |
| 225 | Sulprofos | 92.2 | 0.5 | 86.4 | 2.7 | 82.6 | 2.0 |
| 226 | TCMTB | 99.3 | 6.1 | 93.7 | 8.4 | 83.0 | 10.0 |
| 227 | Tebuconazole | 96.0 | 5.0 | 84.7 | 5.4 | 84.1 | 5.5 |
| 228 | Tebufenozide | 101.8 | 2.1 | 91.4 | 7.9 | 81.4 | 1.1 |
| 229 | Tebupirimfos | 93.3 | 1.9 | 91.2 | 4.0 | 91.6 | 2.2 |
| 230 | Terbuthylazine | 98.4 | 5.2 | 90.0 | 4.6 | 84.7 | 2.5 |
| 231 | Terbutryn | 98.8 | 9.0 | 82.1 | 1.6 | 84.5 | 3.9 |
| 232 | Tetrachlorvinphos | 94.5 | 1.4 | 92.2 | 2.1 | 86.9 | 7.2 |
| 233 | Tetraconazole | 88.9 | 3.2 | 90.4 | 5.1 | 83.2 | 6.5 |
| 234 | Thenylchlor | 91.8 | 1.2 | 83.2 | 3.4 | 86.4 | 4.8 |
| 235 | Thiabendazole | 92.0 | 6.2 | 85.0 | 4.3 | 79.8 | 11.0 |
| 236 | Thiacloprid | 86.4 | 4.1 | 89.4 | 1.9 | 80.7 | 9.4 |
| 237 | Thiazopyr | 89.8 | 6.5 | 85.3 | 4.2 | 87.3 | 9.3 |
| 238 | Thidiazuron | 86.3 | 1.5 | 87.7 | 2.8 | 81.8 | 4.2 |
| 239 | Thifensulfuron-methyl | 88.1 | 4.7 | 83.8 | 3.4 | 87.7 | 3.3 |
| 240 | Thiobencarb | 95.0 | 3.1 | 93.5 | 4.4 | 88.9 | 4.3 |
| 241 | Thiodicarb | 89.3 | 2.3 | 88.6 | 4.2 | 84.4 | 3.9 |
| 242 | Thiophanate-methyl | 96.2 | 2.3 | 97.2 | 3.4 | 84.2 | 2.1 |
| 243 | Tolfenpyrad | 84.4 | 0.3 | 87.7 | 3.8 | 84.3 | 2.1 |
| 244 | Triadimefon | 96.8 | 8.0 | 91.5 | 5.2 | 86.0 | 9.3 |
| 245 | Triadimenol | 100.7 | 12.6 | 87.5 | 8.1 | 81.0 | 10.8 |
| 246 | Tri-allate | 97.7 | 3.2 | 92.8 | 3.5 | 90.2 | 5.7 |
| 247 | Triazophos | 89.7 | 5.6 | 88.7 | 5.2 | 86.3 | 9.7 |
| 248 | Tribenuron-methyl | 97.0 | 2.9 | 87.6 | 4.1 | 84.8 | 1.7 |
| 249 | Tribufos | 89.9 | 1.3 | 88.9 | 3.5 | 89.8 | 5.3 |
| 250 | Trichlorfon | 102.8 | 2.7 | 86.9 | 4.1 | 91.5 | 1.9 |
| 251 | Tricyclazole | 84.7 | 2.2 | 86.1 | 6.6 | 86.4 | 5.0 |
| 252 | Trifloxystrobin | 94.0 | 5.6 | 83.1 | 4.4 | 78.6 | 3.3 |
| 253 | Triflumizole | 94.1 | 2.1 | 90.4 | 1.9 | 87.2 | 3.1 |
| 254 | Triflumuron | 96.3 | 5.5 | 89.6 | 5.7 | 83.8 | 4.1 |
| 255 | Trimethacarb | 96.1 | 1.3 | 85.1 | 5.4 | 85.4 | 2.8 |
| 256 | Triticonazole | 92.6 | 2.8 | 86.9 | 3.2 | 88.4 | 1.3 |
| 257 | Uniconazole | 96.1 | 6.8 | 82.4 | 2.7 | 86.5 | 2.5 |
| 258 | Vamidothion | 88.1 | 3.5 | 81.8 | 4.7 | 82.4 | 5.3 |
| 259 | XMC | 89.9 | 1.4 | 87.9 | 4.6 | 85.3 | 1.0 |
| 260 | Zoxamide | 95.1 | 1.4 | 85.5 | 1.7 | 84.0 | 6.4 |

[1] Recovery.

[2] Not determined.

**Table S2.** Relative area intensities[1] (100 at solvent standard peak area) of 260 pesticides for three versions of urine preparation methods.

| No. | Compound name | Method A | | Method B | | Method C | |
|---|---|---|---|---|---|---|---|
| | | 100 ng/mL | 250 ng/mL | 100 ng/mL | 250 ng/mL | 100 ng/mL | 250 ng/mL |
| 1 | Acetamiprid | 64.8 | 72.5 | 55.7 | 55.7 | 30.4 | 31.4 |
| 2 | Alachlor | 108.9 | 113.7 | 95.9 | 95.4 | 90.3 | 104.6 |
| 3 | Allidochlor | 78.1 | 72.6 | 69.2 | 64.8 | 51.5 | 44.7 |
| 4 | Ametoctradin | 101.3 | 92.7 | 82.4 | 92.2 | 89.1 | 89.8 |
| 5 | Ametryn | 89.6 | 80.2 | 88.8 | 81.3 | 79.0 | 80.1 |
| 6 | Amisulbrom | 94.7 | 109.6 | 97.4 | 78.4 | 84.4 | 89.0 |
| 7 | Anilofos | 105.2 | 94.7 | 100.8 | 96.4 | 91.1 | 99.5 |
| 8 | Asulam | 50.9 | 62.3 | 27.7 | 40.1 | 16.4 | 19.3 |
| 9 | Atrazine | 86.2 | 92.1 | 80.3 | 79.2 | 62.6 | 67.5 |
| 10 | Azaconazole | 86.5 | 71.0 | 68.5 | 66.5 | 68.6 | 58.8 |
| 11 | Azamethiphos | 56.7 | 60.6 | 47.3 | 44.3 | 38.0 | 32.5 |
| 12 | Azimsulfuron | 105.2 | 89.3 | 88.8 | 91.8 | 91.7 | 91.5 |
| 13 | Azinphos-methyl | 79.6 | 94.3 | 77.5 | 85.5 | 58.0 | 69.5 |
| 14 | Azoxystrobin | 92.5 | 92.9 | 77.6 | 85.4 | 73.6 | 74.0 |
| 15 | Bendiocarb | 61.5 | 69.9 | 48.3 | 59.2 | 36.4 | 47.5 |
| 16 | Bensulfuron-methyl | 130.1 | 106.9 | 97.8 | 84.0 | 93.7 | 83.7 |
| 17 | Bentazone | 84.4 | 95.8 | 53.2 | 79.6 | 31.4 | 42.4 |
| 18 | Benthiavalicarb-isopropyl | 108.3 | 83.2 | 99.1 | 69.6 | 98.1 | 76.2 |
| 19 | Benzobicyclon | 97.0 | 85.5 | 78.2 | 71.3 | 84.0 | 69.3 |
| 20 | Benzoximate | 85.9 | 91.6 | 106.8 | 94.6 | 98.2 | 90.7 |
| 21 | Boscalid | 104.8 | 109.3 | 98.7 | 93.3 | 82.4 | 85.8 |
| 22 | Bromacil | 61.0 | 72.0 | 49.9 | 60.4 | 37.4 | 48.6 |
| 23 | Bromobutide | 89.5 | 98.9 | 91.1 | 103.1 | 92.6 | 89.6 |
| 24 | Bupirimate | 92.6 | 94.4 | 72.6 | 86.1 | 74.7 | 75.5 |
| 25 | Buprofezin | 111.9 | 107.6 | 95.8 | 96.1 | 100.7 | 93.8 |
| 26 | Butachlor | 104.8 | 104.0 | 99.1 | 98.6 | 96.1 | 109.0 |
| 27 | Butafenacil | 113.4 | 114.6 | 97.1 | 99.3 | 113.6 | 99.3 |
| 28 | Cadusafos | 91.6 | 97.9 | 96.5 | 86.3 | 104.7 | 82.1 |
| 29 | Carbaryl | 60.3 | 73.9 | 52.5 | 55.5 | 40.0 | 41.3 |
| 30 | Carbendazim | 71.2 | 73.5 | 50.0 | 54.9 | 57.5 | 55.0 |
| 31 | Carbofuran | 85.5 | 86.8 | 66.8 | 80.0 | 59.4 | 60.3 |
| 32 | Carbophenothion | 114.9 | 108.9 | 109.9 | 99.0 | 109.2 | 98.6 |
| 33 | Carboxin | 64.9 | 73.6 | 57.6 | 58.6 | 49.2 | 48.7 |
| 34 | Carpropamid | 97.8 | 78.5 | 76.8 | 66.8 | 78.1 | 68.7 |
| 35 | Cartap | 100.1 | 81.4 | 73.5 | 79.6 | N.D.[2] | N.D. |
| 36 | Chlorantraniliprole | 84.4 | 89.8 | 70.1 | 69.2 | 62.4 | 64.2 |
| 37 | Chlorfenvinphos | 82.2 | 108.7 | 85.2 | 92.1 | 73.8 | 100.2 |
| 38 | Chlorfluazuron | 120.4 | 108.8 | 102.7 | 106.5 | 103.9 | 107.0 |
| 39 | Chloridazon | 42.1 | 41.1 | 27.7 | 29.1 | 17.0 | 15.1 |
| 40 | Chlorotoluron | 57.1 | 60.5 | 47.9 | 47.7 | 40.6 | 43.4 |
| 41 | Chlorsulfuron | 123.2 | 120.8 | 110.4 | 102.8 | 105.0 | 90.8 |
| 42 | Chromafenozide | 86.0 | 81.6 | 110.5 | 91.5 | 82.4 | 84.8 |

| No. | Compound name | Method A | | Method B | | Method C | |
|---|---|---|---|---|---|---|---|
| | | 100 ng/mL | 250 ng/mL | 100 ng/mL | 250 ng/mL | 100 ng/mL | 250 ng/mL |
| 43 | Cinmethylin | 79.4 | 93.4 | 96.3 | 101.1 | 94.6 | 110.4 |
| 44 | Clofentezine | 119.6 | 112.1 | 108.4 | 123.5 | 108.5 | 116.2 |
| 45 | Clomazone | 88.3 | 97.8 | 75.9 | 76.0 | 57.4 | 64.5 |
| 46 | Clothianidin | 44.3 | 59.0 | 45.4 | 48.1 | 27.7 | 25.4 |
| 47 | Cyanazine | 86.7 | 88.6 | 66.5 | 60.0 | 58.4 | 57.4 |
| 48 | Cyazofamid | 118.1 | 113.4 | 99.7 | 102.4 | 105.2 | 104.7 |
| 49 | Cyclosulfamuron | 103.8 | 99.8 | 77.0 | 78.7 | 78.5 | 71.7 |
| 50 | Cymoxanil | 55.9 | 54.4 | 37.6 | 39.3 | 23.3 | 26.8 |
| 51 | Cyproconazole | 97.5 | 83.6 | 95.5 | 80.7 | 88.1 | 88.8 |
| 52 | Cyprodinil | 99.2 | 97.9 | 79.6 | 83.6 | 93.1 | 70.3 |
| 53 | Daimuron | 91.8 | 97.5 | 81.0 | 87.1 | 68.5 | 84.2 |
| 54 | Diazinon | 113.9 | 108.4 | 98.3 | 101.5 | 118.8 | 103.5 |
| 55 | Dicrotophos | 90.5 | 79.6 | 71.9 | 71.3 | 44.8 | 41.3 |
| 56 | Diethofencarb | 95.0 | 83.9 | 90.4 | 81.7 | 77.7 | 73.3 |
| 57 | Difenoconazole | 118.9 | 115.8 | 115.6 | 114.5 | 121.3 | 115.1 |
| 58 | Diflubenzuron | 109.2 | 129.2 | 95.0 | 113.1 | 88.5 | 102.9 |
| 59 | Diflufenican | 98.3 | 81.6 | 85.7 | 86.2 | 85.3 | 82.3 |
| 60 | Dimethachlor | 97.0 | 101.6 | 79.1 | 82.3 | 69.6 | 68.3 |
| 61 | Dimethametryn | 106.5 | 101.2 | 87.9 | 89.8 | 84.4 | 90.4 |
| 62 | Dimethenamid | 83.6 | 89.8 | 69.8 | 82.9 | 72.0 | 64.4 |
| 63 | Dimethoate | 57.2 | 62.8 | 52.1 | 53.0 | 28.6 | 25.8 |
| 64 | Dimethomorph | 137.5 | 127.4 | 108.9 | 112.5 | 100.9 | 87.1 |
| 65 | Diniconazole | 109.9 | 116.6 | 116.7 | 107.3 | 114.3 | 114.8 |
| 66 | Diphenamid | 91.4 | 80.8 | 78.9 | 79.0 | 72.5 | 72.4 |
| 67 | Diuron | 79.3 | 86.4 | 63.2 | 76.6 | 57.1 | 66.3 |
| 68 | Edifenphos | 113.5 | 122.8 | 121.2 | 98.0 | 107.9 | 112.8 |
| 69 | Emamectin B1a | 118.1 | 123.2 | 123.2 | 112.2 | 119.2 | 115.0 |
| 70 | Emamectin B1b | 119.6 | 132.6 | 132.5 | 116.6 | 114.5 | 137.5 |
| 71 | EPN | 84.5 | 93.2 | 81.7 | 74.3 | 66.7 | 73.0 |
| 72 | Epoxiconazole | 108.3 | 99.2 | 106.2 | 100.4 | 103.4 | 110.8 |
| 73 | Ethaboxam (EBX) | 82.1 | 100.2 | 80.8 | 86.0 | 64.3 | 80.8 |
| 74 | Ethametsulfuron-methyl | 105.9 | 117.3 | 92.1 | 96.5 | 80.7 | 83.3 |
| 75 | Ethiofencarb | 67.5 | 70.0 | 62.1 | 64.0 | 53.1 | 50.3 |
| 76 | Ethion | 111.6 | 110.0 | 107.8 | 96.0 | 99.5 | 104.4 |
| 77 | Ethoprophos | 100.5 | 92.8 | 76.1 | 85.4 | 77.5 | 81.1 |
| 78 | Ethoxyquin | 107.6 | 94.3 | 75.5 | 80.9 | 70.5 | 70.6 |
| 79 | Ethoxysulfuron | 148.8 | 142.9 | 121.4 | 111.9 | 122.5 | 130.5 |
| 80 | Etoxazole | 94.0 | 103.8 | 88.9 | 75.8 | 88.6 | 84.8 |
| 81 | Etrimfos | 94.9 | 107.0 | 107.4 | 102.6 | 100.5 | 98.7 |
| 82 | Fenamidone | 110.6 | 110.9 | 84.6 | 91.7 | 76.2 | 74.2 |
| 83 | Fenamiphos | 113.2 | 109.6 | 101.0 | 96.5 | 95.3 | 89.3 |
| 84 | Fenazaquin | 106.2 | 107.6 | 91.5 | 84.8 | 87.6 | 86.2 |
| 85 | Fenbuconazole | 99.7 | 92.7 | 94.1 | 106.7 | 90.9 | 111.3 |
| 86 | Fenhexamid | 122.0 | 114.0 | 112.8 | 110.1 | 115.9 | 103.4 |

| No. | Compound name | Method A | | Method B | | Method C | |
|---|---|---|---|---|---|---|---|
| | | 100 ng/mL | 250 ng/mL | 100 ng/mL | 250 ng/mL | 100 ng/mL | 250 ng/mL |
| 87 | Fenobucarb (BPMC) | 95.4 | 92.4 | 72.3 | 79.0 | 66.1 | 73.4 |
| 88 | Fenothiocarb | 90.6 | 92.4 | 87.5 | 89.8 | 70.2 | 73.3 |
| 89 | Fenoxanil | 94.7 | 91.5 | 89.9 | 87.0 | 78.3 | 77.4 |
| 90 | Fenoxycarb | 98.3 | 83.1 | 80.7 | 81.9 | 80.9 | 89.3 |
| 91 | Fenthion | 109.7 | 102.9 | 100.2 | 100.8 | 98.8 | 111.0 |
| 92 | Ferimzone | 89.4 | 100.9 | 75.8 | 79.0 | 58.5 | 61.0 |
| 93 | Fipronil | 129.4 | 115.6 | 125.8 | 118.0 | 145.1 | 126.6 |
| 94 | Fluacrypyrim | 88.0 | 85.9 | 98.6 | 106.6 | 95.3 | 92.4 |
| 95 | Fluazinam | 110.3 | 116.8 | 114.1 | 108.6 | 113.5 | 109.5 |
| 96 | Flucetosulfuron | 180.7 | 179.8 | 133.4 | 133.9 | 131.9 | 147.6 |
| 97 | Flufenacet | 75.0 | 83.2 | 82.3 | 86.3 | 74.2 | 96.0 |
| 98 | Flufenoxuron | 107.9 | 117.8 | 99.7 | 101.9 | 103.9 | 106.8 |
| 99 | Fluopicolide | 97.6 | 128.1 | 80.6 | 88.1 | 73.5 | 83.0 |
| 100 | Fluopyram | 84.7 | 88.0 | 83.0 | 73.3 | 77.3 | 82.8 |
| 101 | Flusilazole | 106.6 | 103.5 | 100.3 | 99.5 | 82.9 | 90.0 |
| 102 | Flusulfamide | 102.3 | 119.7 | 114.6 | 121.7 | 109.7 | 133.9 |
| 103 | Flutolanil | 112.6 | 91.5 | 92.2 | 78.3 | 77.5 | 71.0 |
| 104 | Fluxapyroxad | 102.7 | 91.2 | 88.8 | 60.9 | 68.9 | 66.1 |
| 105 | Fonofos | 114.3 | 101.2 | 117.2 | 100.3 | 114.0 | 90.3 |
| 106 | Forchlorfenuron | 95.7 | 98.5 | 86.4 | 85.9 | 80.2 | 79.7 |
| 107 | Fosthiazate | 73.0 | 69.6 | 62.1 | 58.5 | 50.9 | 51.7 |
| 108 | Furathiocarb | 95.1 | 98.7 | 89.9 | 95.8 | 97.5 | 101.2 |
| 109 | Halosulfuron-methyl | 143.6 | 124.5 | 123.3 | 112.4 | 123.6 | 123.3 |
| 110 | Haloxyfop-R-Methyl | 111.0 | 110.4 | 99.5 | 104.1 | 95.3 | 111.7 |
| 111 | Hexaconazole | 99.4 | 114.5 | 111.5 | 106.3 | 102.3 | 111.9 |
| 112 | Hexaflumuron | 94.1 | 103.2 | 126.4 | 124.5 | 117.7 | 113.8 |
| 113 | Hexazinone | 70.8 | 84.8 | 58.7 | 62.7 | 48.0 | 52.8 |
| 114 | Imazalil | 94.8 | 73.4 | 76.2 | 66.2 | 68.1 | 60.0 |
| 115 | Imazapic | 58.3 | 63.7 | 36.6 | 41.8 | 29.3 | 29.0 |
| 116 | Imazaquin | 85.6 | 87.8 | 70.6 | 81.4 | 66.0 | 75.4 |
| 117 | Imazethapyr | 61.2 | 55.2 | 41.3 | 42.0 | 39.8 | 35.1 |
| 118 | Imibenconazole | 110.2 | 99.3 | 111.7 | 91.8 | 100.5 | 95.8 |
| 119 | Imicyafos | 64.6 | 85.3 | 57.6 | 55.4 | 41.4 | 37.5 |
| 120 | Imidacloprid | 76.7 | 72.9 | 66.1 | 51.9 | 32.4 | 30.4 |
| 121 | Indoxacarb | 101.9 | 100.5 | 93.7 | 100.3 | 97.3 | 101.2 |
| 122 | Iprobenfos | 86.6 | 86.6 | 65.4 | 67.2 | 57.2 | 64.1 |
| 123 | Iprovalicarb | 97.6 | 99.7 | 91.7 | 89.8 | 90.8 | 77.6 |
| 124 | Isazofos | 102.7 | 105.4 | 79.5 | 88.9 | 88.7 | 76.9 |
| 125 | Isoprocarb | 72.9 | 61.4 | 58.8 | 59.0 | 50.2 | 54.4 |
| 126 | Isoprothiolane | 105.7 | 100.7 | 73.1 | 84.0 | 76.5 | 74.5 |
| 127 | Isoproturon | 89.0 | 94.3 | 82.7 | 83.3 | 66.2 | 70.5 |
| 128 | Isopyrazam | 92.9 | 110.1 | 92.0 | 84.9 | 88.9 | 82.3 |
| 129 | Isoxathion | 99.0 | 119.6 | 99.9 | 99.7 | 91.4 | 103.6 |
| 130 | Kresoxim-methyl | 90.2 | 97.6 | 73.7 | 77.7 | 69.7 | 75.7 |

| No. | Compound name | Method A | | Method B | | Method C | |
|---|---|---|---|---|---|---|---|
| | | 100 ng/mL | 250 ng/mL | 100 ng/mL | 250 ng/mL | 100 ng/mL | 250 ng/mL |
| 131 | Linuron | 81.9 | 82.3 | 89.3 | 79.8 | 61.7 | 76.1 |
| 132 | Mandipropamid | 98.2 | 92.2 | 71.4 | 77.8 | 70.2 | 66.4 |
| 133 | Mecarbam | 90.5 | 96.9 | 109.3 | 98.8 | 94.5 | 112.9 |
| 134 | Mefenacet | 102.0 | 109.9 | 83.0 | 90.7 | 96.1 | 79.5 |
| 135 | Mefenpyr-diethyl | 92.9 | 72.6 | 83.0 | 82.1 | 92.4 | 74.4 |
| 136 | Mepronil | 96.4 | 103.9 | 76.2 | 88.6 | 70.8 | 79.7 |
| 137 | Metalaxyl | 89.4 | 85.6 | 66.5 | 73.7 | 64.9 | 64.2 |
| 138 | Metamifop | 110.7 | 104.2 | 118.0 | 96.4 | 116.7 | 107.1 |
| 139 | Metazosulfuron | 162.4 | 141.4 | 157.3 | 130.8 | 139.4 | 134.4 |
| 140 | Metconazole | 109.6 | 109.4 | 103.8 | 111.1 | 104.7 | 96.8 |
| 141 | Methabenzthiazuron | 71.2 | 70.0 | 58.5 | 63.4 | 58.6 | 54.0 |
| 142 | Methidathion | 83.0 | 86.5 | 82.7 | 93.6 | 79.5 | 70.8 |
| 143 | Methiocarb | 83.0 | 80.2 | 79.6 | 66.9 | 64.5 | 63.1 |
| 144 | Methomyl | 50.3 | 49.6 | 36.7 | 38.5 | 13.9 | 16.4 |
| 145 | Methoxyfenozide | 88.4 | 63.0 | 62.7 | 62.5 | 64.4 | 57.7 |
| 146 | Metobromuron | 63.3 | 50.3 | 48.8 | 50.6 | 34.3 | 42.7 |
| 147 | Metolachlor | 92.7 | 89.0 | 83.9 | 74.7 | 73.9 | 71.1 |
| 148 | Metolcarb | 69.0 | 67.4 | 50.5 | 65.0 | 39.7 | 45.8 |
| 149 | Metominostrobin | 80.2 | 94.8 | 60.4 | 81.3 | 62.1 | 77.7 |
| 150 | Metrafenone | 118.2 | 109.5 | 119.1 | 114.3 | 122.0 | 96.5 |
| 151 | Mevinphos | 68.5 | 66.2 | 46.8 | 40.0 | 27.7 | 26.3 |
| 152 | Milbemectin A4 | 116.6 | 108.0 | 117.8 | 116.4 | 147.0 | 115.6 |
| 153 | Molinate | 107.8 | 119.4 | 102.3 | 105.7 | 97.1 | 104.7 |
| 154 | Monocrotophos | 60.1 | 65.4 | 47.1 | 52.0 | 23.7 | 26.9 |
| 155 | Myclobutanil | 133.0 | 94.1 | 122.6 | 98.0 | 104.5 | 110.2 |
| 156 | Napropamide | 87.3 | 90.9 | 84.4 | 78.0 | 80.2 | 87.0 |
| 157 | Nicosulfuron | 86.7 | 93.4 | 63.9 | 66.5 | 53.4 | 58.0 |
| 158 | Nitenpyram | 132.4 | 136.2 | 60.4 | 54.6 | 30.6 | 25.5 |
| 159 | Nuarimol | 106.6 | 103.1 | 103.4 | 101.7 | 79.4 | 90.2 |
| 160 | Ofurace | 69.4 | 74.2 | 54.2 | 59.8 | 44.2 | 40.9 |
| 161 | Omethoate | 40.8 | 45.3 | 33.3 | 34.6 | 19.9 | 17.2 |
| 162 | Orysastrobin | 94.9 | 88.0 | 101.4 | 89.7 | 89.3 | 92.2 |
| 163 | Oxadiazon | 109.7 | 116.0 | 112.6 | 120.1 | 111.0 | 104.4 |
| 164 | Oxadixyl | 51.1 | 54.5 | 33.6 | 36.3 | 22.1 | 26.6 |
| 165 | Oxamyl | 56.7 | 51.7 | 44.3 | 43.0 | 16.9 | 17.7 |
| 166 | Oxydemeton-methyl | 56.3 | 63.8 | 50.6 | 47.6 | 26.5 | 27.7 |
| 167 | Paclobutrazol | 101.4 | 81.6 | 85.5 | 82.9 | 62.3 | 78.7 |
| 168 | Pebulate | 119.0 | 111.0 | 103.0 | 124.9 | 114.9 | 104.8 |
| 169 | Penconazole | 87.2 | 114.6 | 90.8 | 101.8 | 90.1 | 104.7 |
| 170 | Pendimethalin | 98.3 | 109.4 | 100.6 | 100.1 | 94.8 | 97.8 |
| 171 | Penoxsulam | 122.4 | 117.6 | 114.7 | 102.7 | 82.4 | 89.2 |
| 172 | Penthiopyrad | 113.7 | 95.1 | 89.2 | 93.8 | 84.2 | 90.0 |
| 173 | Phenmedipham | 89.1 | 96.9 | 76.2 | 82.1 | 67.9 | 68.7 |
| 174 | Phenthoate | 89.1 | 107.7 | 84.0 | 93.0 | 69.2 | 80.3 |

| No. | Compound name | Method A | | Method B | | Method C | |
|---|---|---|---|---|---|---|---|
| | | 100 ng/mL | 250 ng/mL | 100 ng/mL | 250 ng/mL | 100 ng/mL | 250 ng/mL |
| 175 | Phosalone | 110.0 | 123.1 | 120.9 | 110.0 | 101.9 | 111.5 |
| 176 | Phosmet | 83.8 | 106.0 | 84.3 | 92.8 | 65.5 | 72.1 |
| 177 | Phosphamidon | 77.3 | 81.1 | 60.4 | 64.3 | 48.2 | 52.2 |
| 178 | Phoxim | 106.0 | 98.6 | 93.8 | 97.8 | 95.3 | 106.8 |
| 179 | Picolinafen | 111.6 | 119.2 | 113.5 | 113.5 | 121.4 | 111.5 |
| 180 | Picoxystrobin | 86.0 | 92.3 | 96.9 | 84.3 | 90.7 | 88.2 |
| 181 | Piperophos | 107.3 | 111.9 | 101.4 | 102.1 | 112.0 | 104.9 |
| 182 | Pirimicarb | 81.1 | 88.7 | 64.8 | 69.8 | 52.6 | 56.8 |
| 183 | Pirimiphos-ethyl | 103.3 | 100.4 | 101.3 | 102.1 | 111.7 | 103.8 |
| 184 | Pirimiphos-methyl | 103.1 | 106.9 | 92.8 | 113.6 | 95.1 | 106.8 |
| 185 | Pretilachlor | 107.0 | 103.2 | 105.1 | 95.4 | 105.4 | 96.2 |
| 186 | Probenazole | 54.1 | 51.2 | 39.6 | 40.0 | 26.9 | 28.8 |
| 187 | Prochloraz | 104.6 | 110.1 | 111.5 | 100.0 | 110.5 | 95.0 |
| 188 | Promecarb | 89.5 | 90.0 | 81.5 | 72.4 | 69.4 | 67.8 |
| 189 | Prometryn | 100.2 | 103.5 | 86.3 | 82.8 | 72.9 | 82.3 |
| 190 | Propachlor | 84.5 | 83.8 | 71.5 | 70.1 | 66.9 | 73.0 |
| 191 | Propazine | 84.7 | 90.6 | 82.6 | 83.0 | 63.3 | 68.0 |
| 192 | Propiconazole | 108.1 | 121.1 | 115.2 | 108.2 | 125.7 | 117.1 |
| 193 | Propisochlor | 101.7 | 107.6 | 95.4 | 93.8 | 94.4 | 97.2 |
| 194 | Propoxur | 59.1 | 65.8 | 56.0 | 53.9 | 38.2 | 40.3 |
| 195 | Prothiofos | 106.3 | 106.2 | 106.6 | 115.2 | 105.7 | 99.3 |
| 196 | Pyraclofos | 93.6 | 84.4 | 89.2 | 90.7 | 83.9 | 97.9 |
| 197 | Pyraclostrobin | 114.0 | 105.7 | 107.3 | 96.7 | 99.6 | 90.8 |
| 198 | Pyrazolynate | 95.8 | 85.7 | 87.3 | 69.2 | 86.5 | 75.8 |
| 199 | Pyrazophos | 103.6 | 98.1 | 99.6 | 91.5 | 103.6 | 93.8 |
| 200 | Pyrazoxyfen | 98.7 | 95.9 | 101.9 | 81.6 | 93.2 | 84.1 |
| 201 | Pyribenzoxim | 105.7 | 99.1 | 85.2 | 79.5 | 82.7 | 92.9 |
| 202 | Pyributicarb | 85.5 | 94.5 | 93.5 | 95.1 | 99.3 | 93.6 |
| 203 | Pyridaben | 90.4 | 90.6 | 88.6 | 90.5 | 81.9 | 80.9 |
| 204 | Pyridalyl | 124.1 | 116.4 | 110.5 | 109.6 | 102.1 | 103.3 |
| 205 | Pyridaphenthion | 95.5 | 83.0 | 77.7 | 74.5 | 64.0 | 71.9 |
| 206 | Pyridate | 103.3 | 103.9 | 104.8 | 100.1 | 110.4 | 97.5 |
| 207 | Pyrifenox | 86.6 | 80.6 | 75.3 | 83.8 | 74.8 | 74.2 |
| 208 | Pyriminobac-methyl E | 98.2 | 91.5 | 84.9 | 88.8 | 94.5 | 81.0 |
| 209 | Pyriminobac-methyl Z | 93.9 | 86.2 | 86.1 | 74.8 | 83.6 | 79.0 |
| 210 | Pyrimisulfan | 93.2 | 109.9 | 78.2 | 71.2 | 73.6 | 60.5 |
| 211 | Pyriproxyfen | 87.9 | 95.9 | 91.6 | 95.5 | 101.7 | 86.7 |
| 212 | Pyroquilon | 60.2 | 73.7 | 56.5 | 60.1 | 31.6 | 45.5 |
| 213 | Quinoclamine | 50.7 | 48.4 | 40.9 | 43.2 | 38.8 | 34.9 |
| 214 | Rimsulfuron | 104.0 | 109.0 | 93.3 | 99.2 | 87.3 | 78.1 |
| 215 | Saflufenacil | 184.2 | 184.4 | 141.0 | 160.2 | 138.4 | 134.6 |
| 216 | Sethoxydim | 84.5 | 84.6 | 74.1 | 80.2 | 90.3 | 78.6 |
| 217 | Simazine | 64.7 | 57.9 | 48.9 | 50.2 | 39.4 | 40.0 |
| 218 | Simeconazole | 91.9 | 111.4 | 110.7 | 103.8 | 100.6 | 107.4 |

| No. | Compound name | Method A | | Method B | | Method C | |
|---|---|---|---|---|---|---|---|
| | | 100 ng/mL | 250 ng/mL | 100 ng/mL | 250 ng/mL | 100 ng/mL | 250 ng/mL |
| 219 | Simetryn | 76.1 | 83.4 | 67.7 | 75.5 | 63.2 | 67.6 |
| 220 | Spinetoram (XDE-175-J) | 97.9 | 102.0 | 81.0 | 96.8 | 98.3 | 106.5 |
| 221 | Spinosyn A | 97.3 | 86.5 | 91.0 | 84.3 | 93.8 | 91.4 |
| 222 | Spinosyn D | 116.6 | 111.0 | 109.7 | 103.0 | 99.3 | 105.8 |
| 223 | Spirodiclofen | 92.1 | 101.4 | 92.3 | 94.3 | 93.2 | 99.5 |
| 224 | Sulfoxaflor | 38.3 | 50.5 | 38.9 | 42.4 | 19.5 | 20.8 |
| 225 | Sulprofos | 97.4 | 109.2 | 112.4 | 97.9 | 103.0 | 101.5 |
| 226 | TCMTB | 96.9 | 94.2 | 102.7 | 85.8 | 77.8 | 83.2 |
| 227 | Tebuconazole | 106.4 | 98.4 | 92.6 | 103.0 | 93.3 | 105.7 |
| 228 | Tebufenozide | 77.7 | 83.2 | 81.0 | 94.3 | 94.8 | 84.8 |
| 229 | Tebupirimfos | 92.5 | 94.0 | 81.4 | 94.0 | 79.6 | 81.0 |
| 230 | Terbuthylazine | 86.4 | 81.7 | 70.2 | 67.7 | 59.5 | 59.7 |
| 231 | Terbutryn | 90.5 | 81.5 | 82.0 | 82.5 | 85.8 | 76.0 |
| 232 | Tetrachlorvinphos | 100.7 | 103.0 | 101.1 | 102.6 | 92.6 | 96.0 |
| 233 | Tetraconazole | 90.5 | 99.0 | 97.6 | 99.5 | 97.0 | 96.8 |
| 234 | Thenylchlor | 87.7 | 96.7 | 94.0 | 99.5 | 85.8 | 97.6 |
| 235 | Thiabendazole | 46.2 | 50.2 | 42.9 | 38.5 | 24.2 | 20.1 |
| 236 | Thiacloprid | 45.5 | 45.5 | 26.3 | 24.0 | 18.4 | 17.5 |
| 237 | Thiazopyr | 101.8 | 112.0 | 98.5 | 101.1 | 108.4 | 103.1 |
| 238 | Thidiazuron | 114.2 | 111.4 | 83.5 | 89.0 | 74.2 | 75.4 |
| 239 | Thifensulfuron-methyl | 99.9 | 90.7 | 72.0 | 80.3 | 63.4 | 62.9 |
| 240 | Thiobencarb | 119.5 | 116.8 | 105.9 | 99.7 | 115.2 | 93.6 |
| 241 | Thiodicarb | 88.5 | 89.3 | 78.7 | 78.5 | 69.7 | 68.8 |
| 242 | Thiophanate-methyl | 51.7 | 51.6 | 34.4 | 35.8 | 26.7 | 30.6 |
| 243 | Tolfenpyrad | 113.3 | 132.1 | 106.4 | 102.2 | 109.3 | 112.3 |
| 244 | Triadimefon | 124.0 | 109.0 | 116.0 | 93.1 | 92.5 | 91.5 |
| 245 | Triadimenol | 136.5 | 112.7 | 125.9 | 130.7 | 122.7 | 121.3 |
| 246 | Tri-allate | 115.7 | 121.8 | 121.4 | 105.7 | 108.5 | 117.2 |
| 247 | Triazophos | 101.7 | 101.5 | 80.6 | 93.0 | 91.4 | 85.2 |
| 248 | Tribenuron-methyl | 86.7 | 73.3 | 58.9 | 54.5 | 56.5 | 54.3 |
| 249 | Tribufos | 95.6 | 110.0 | 98.3 | 97.5 | 96.1 | 82.4 |
| 250 | Trichlorfon | 67.2 | 71.9 | 58.2 | 60.9 | 32.1 | 36.2 |
| 251 | Tricyclazole | 40.3 | 43.1 | 28.9 | 29.4 | 17.6 | 17.6 |
| 252 | Trifloxystrobin | 99.2 | 91.3 | 86.5 | 87.9 | 102.0 | 88.8 |
| 253 | Triflumizole | 85.6 | 95.1 | 82.7 | 84.1 | 86.6 | 84.2 |
| 254 | Triflumuron | 122.0 | 106.6 | 99.6 | 106.3 | 107.8 | 112.5 |
| 255 | Trimethacarb | 90.6 | 97.5 | 85.2 | 86.0 | 69.2 | 70.4 |
| 256 | Triticonazole | 104.7 | 90.8 | 97.8 | 94.9 | 158.8 | 152.5 |
| 257 | Uniconazole | 100.3 | 97.0 | 97.3 | 93.1 | 140.4 | 155.3 |
| 258 | Vamidothion | 71.5 | 82.4 | 54.6 | 60.7 | 34.9 | 35.2 |
| 259 | XMC | 75.0 | 93.1 | 71.8 | 73.1 | 14.2 | 14.8 |
| 260 | Zoxamide | 115.3 | 108.5 | 107.5 | 106.4 | 44.4 | 44.0 |

[1] Relative peak intensity $= \left(\frac{\text{Peak area of post spiked sample}}{\text{Peak area of solvent standard}}\right) \times 100$.

[2] Not determined.

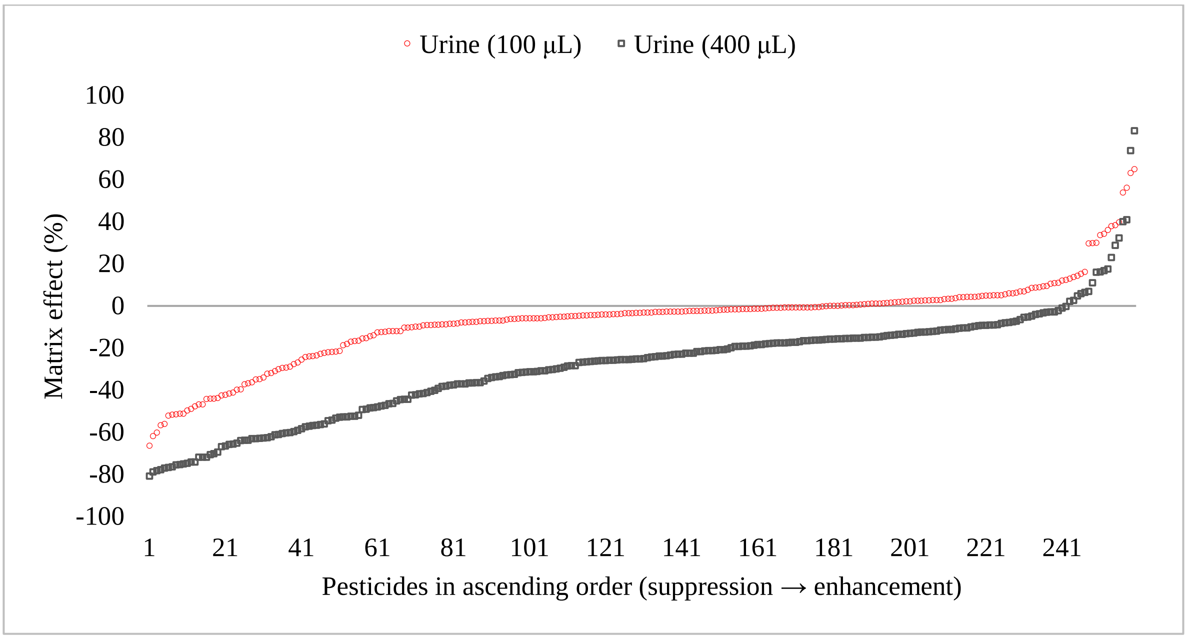


**Figure S1.** Matrix effects of 260 pesticides in 100 µL and 400 µL of urine samples. The samples were extracted with the same volumes of solvent (400 µL). Each graph was plotted against the number of pesticides ranked by matrix effect values (%).

© 2019 by the authors. Submitted for possible open access publication under the terms and conditions of the Creative Commons Attribution (CC BY) license (http://creativecommons.org/licenses/by/4.0/).
